# Improving HIV Prevention Among Heterosexual Men Seeking Sexually Transmitted Infection Services in Malawi: Protocol for a Type I Effectiveness-Implementation Hybrid Randomized Controlled Trial of Systems Navigator–Delivered Integrated Prevention Package (HPTN 112-NJIRA Study)

**DOI:** 10.2196/72981

**Published:** 2025-06-18

**Authors:** Sarah E Rutstein, Laura Limarzi-Klyn, Jane S Chen, Yaw O Agyei, Shahnaz Ahmed, Ian Bell, Myron Cohen, Jessica M Fogel, Vivian Go, Dan Haines, Erica L Hamilton, Irving F Hoffman, Mina C Hosseinipour, Mark A Marzinke, William C Miller, Mathews Mukatipa, Julie Pulerwitz, Hans M L Spiegel, Ting Ye, Mitch Matoga

**Affiliations:** 1 Department of Medicine University of North Carolina at Chapel Hill Chapel Hill, NC United States; 2 Department of Health Behavior Gillings School of Global Public Health University of North Carolina at Chapel Hill Chapel Hill, NC United States; 3 Department of Pathology Johns Hopkins University School of Medicine Baltimore, MD United States; 4 University of Washington Department of Biostatistics HPTN Statistical and Data Management Center Seattle, WA United States; 5 Network and Collaborative Research Division FHI 360 Durham, NC United States; 6 Department of Medicine Johns Hopkins University School of Medicine Baltimore, MD United States; 7 Department of Epidemiology Gillings School of Global Public Health University of North Carolina at Chapel Hill Chapel Hill, NC United States; 8 UNC Project Malawi Lilongwe Malawi; 9 Population Council Washington, DC United States; 10 Kelly Government Solutions Contractor to National Institute of Allergy and Infectious Diseases National Institutes of Health, HHS Rockville, MD United States

**Keywords:** hybrid, long-acting injectable, preexposure prophylaxis, peer, heterosexual, HIV prevention, bacterial STI

## Abstract

**Background:**

Preexposure prophylaxis (PrEP) remains one of the most efficacious interventions for preventing HIV, but its effectiveness is often limited by poor persistence. Although regional efforts have primarily focused on young women and men who have sex with men, heterosexual men in East and Southern Africa represent a crucial group to engage and retain in PrEP care—both to improve health outcomes for men and to interrupt HIV transmission chains. Men seeking sexually transmitted infection (STI) services are particularly vulnerable to HIV acquisition, yet only a few interventions have tested strategies for engaging and retaining these men in PrEP services. Systems navigation, which addresses barriers to health care access and enhances comfort in clinical settings, may offer a promising approach to improving persistent PrEP use among heterosexual men.

**Objective:**

This study will assess the effect of a peer-delivered systems navigator–facilitated HIV prevention package on PrEP persistence at 26 weeks among heterosexual men seeking STI clinical services in Lilongwe, Malawi. It will also evaluate the acceptability of the intervention and barriers to implementation among key stakeholders. Insights will inform the feasibility of a future randomized controlled trial.

**Methods:**

In this single-site pilot type I effectiveness-implementation hybrid randomized controlled trial, 200 heterosexual men seeking STI services and initiated on PrEP in Lilongwe, Malawi, will be randomized (1:2) to standard-of-care PrEP services or systems navigator–assisted PrEP care (intervention). Participants will be followed every 13 weeks for at least 26 and up to 52 weeks. PrEP use and engagement in care will be assessed through medical record review and intraerythrocytic tenofovir diphosphate measurement, using objective biomedical analyses via dried blood spot. Primary effectiveness and implementation outcomes include 26-week PrEP persistence (adapted to accommodate daily oral, event-driven oral, or injectable PrEP) and acceptability, respectively. Additional implementation outcomes include feasibility and cost. Exploratory objectives characterize preferences for PrEP modalities, perceived and experienced stigma, and the influence of gender norms on PrEP persistence. All clinical services, including the provision of PrEP and PrEP safety monitoring, are being conducted by the Malawi Ministry of Health.

**Results:**

HPTN (HIV Prevention Trials Network) 112 was funded in November 2023. Study recruitment began in April 2024 and closed in November 2024. As of February 3, 2025, the study has enrolled 199 participants, with follow-up expected through June 2025. No interim analyses were planned; data analysis for primary end points is expected in the summer of 2025.

**Conclusions:**

Improving PrEP use outcomes among heterosexual men in East and Southern Africa is critical to interrupting HIV transmission. This study offers unique insights into a low-resource, potentially scalable intervention, focusing on a group of men at particularly high risk of HIV acquisition—those with recent STIs. The hybrid RCT design addresses clinically relevant effectiveness questions and explores key determinants that will inform future multisite implementation trials.

**Trial Registration:**

ClinicalTrials.gov NCT06200545; https://clinicaltrials.gov/study/NCT06200545

**International Registered Report Identifier (IRRID):**

DERR1-10.2196/72981

## Introduction

Despite significant reductions in incidence globally, progress toward ending the HIV epidemic has stalled [1]. Biomedical preexposure prophylaxis (PrEP) remains one of the most effective evidence-based interventions for preventing HIV; however, its rollout has been hindered by inconsistent referral, inadequate uptake, poor adherence, and frequent discontinuation [2-7]. In East and Southern Africa, most incident HIV acquisitions occur through heterosexual transmission [8]. Women bear a disproportionate burden of new infections; accordingly, PrEP engagement efforts have historically focused on women. HIV acquisitions among women are frequently driven by male-female age-disparate partnerships and transactional sex, where women may face challenges such as negotiating condom use or adhering to PrEP due to fear of partner disapproval. Engaging heterosexual men in prevention-effective PrEP use—defined as persistent PrEP use before and immediately after potential HIV exposure—is therefore critical to improving HIV prevention outcomes for both men and women.

Despite their relevance in the HIV epidemic, relatively little is known about best practices for effectively engaging and retaining heterosexual men in PrEP care across different global settings. Modifying perceptions of HIV risk and reducing stigma toward PrEP use can improve PrEP persistence among men [9,10]. Longer-acting agents, such as long-acting injectable cabotegravir (CAB-LA), present a promising opportunity to enhance PrEP effectiveness by eliminating barriers associated with daily pill use. Although landmark studies have focused on men and transgender women who have sex with men [11] and on women in high HIV incidence settings [12], CAB-LA is expected to offer similar HIV prevention effectiveness among heterosexual men. Early rollout pilot prevention studies suggest that CAB-LA is a popular choice for men in Africa [13-15]. Despite the promise of newer and longer-acting PrEP agents [16], their effectiveness—and indeed, cost-effectiveness—will only be realized if those most vulnerable to HIV are engaged and remain persistent throughout periods of risk.

In Malawi, men have not historically been the focus of PrEP interventions. Consistent with World Health Organization (WHO) recommendations, individuals with a recent sexually transmitted infection (STI) and those involved in transactional sex are considered priority populations for PrEP in Malawi [17]. Previous research has examined the integration of PrEP and STI services at one of the country’s largest urban STI clinics in Lilongwe [18]. STI clinics are a critical yet often underutilized pathway to PrEP services in low- and middle-income countries [19], and may serve as an especially important entry point for men, who may not access health care services as regularly as their female counterparts. Despite declining population-level HIV incidence, our prior work has shown that individuals presenting with STIs continue to have persistently high rates of acute HIV infection [20-22]. Between March and December 2022, 835 individuals initiated PrEP at the STI clinic in Lilongwe, the majority of whom were male (55%) [23]. Nearly two-thirds of all PrEP initiators had a current or recent (within the past 6 months) STI, and nearly one-third reported transactional sex (ie, exchanging sex for goods, money, or favors); these categories were not mutually exclusive. Persistence across the population of all PrEP users was low—estimated at 3.7% at 6 months. Among male PrEP initiators with an STI at the time of PrEP initiation or within the prior 6 months, only 2.4% were persistent (95% CI 1.4-4.1) at 6 months. As with all PrEP outcome evaluations, persistent use must be contextualized by ongoing HIV risk. Among a subgroup of enrolled men initiating PrEP during STI clinic visits, incident infections in the subsequent 6 months were common—55.8 incident *Chlamydia trachomatis, Neisseria gonorrhoeae,* or *Treponema pallidum* infections per 100 person-years [24]. Improving persistent PrEP use among men with current or recent curable STIs may help direct scarce resources to a group in particular need of enhanced PrEP retention efforts.

Systems navigation, including psychosocial support and assistance in maneuvering through sometimes complex or burdensome health care systems, is a promising approach [25,26], though data specific to heterosexual men and PrEP services remain limited. Systems navigation can be conceptualized broadly—encompassing peer-based interventions, psychosocial counseling, and care navigation— generally focuses on addressing barriers to health care access and enhancing comfort and confidence within clinical settings.

. Peer-based models may improve HIV care and outcomes, and the flexibility of this model may be particularly appealing in more resource-poor settings [27]. Promising evidence has emerged from the United States demonstrating that tailored navigation services can help individuals overcome systems-related barriers to PrEP care [28,29], including when integrated into sexual health clinics [30]. Among heterosexual men in South Africa, health care providers and educational materials that encouraged PrEP uptake influenced PrEP use. Increasing individual support, simplifying access to PrEP, and navigator-delivered sexual health and HIV information were identified as effective intervention strategies [31,32]. In Eswatini, male-friendly services were highlighted as key future priorities to improve PrEP use among men, including integrated or community-based PrEP delivery models [9]. Understanding the acceptability, feasibility, and effectiveness of peer-delivered navigation services as a strategy to improve PrEP persistence specifically for heterosexual men can inform HIV prevention programming for a population that has, thus far, rarely taken the spotlight in prevention efforts in East and Southern Africa. This group may face distinct challenges to PrEP persistence related to perceived gender norms, stigma, access to health care services, and the perceived relevance of PrEP within their broader HIV and STI prevention strategies.

NJIRA (Navigated Journey to Improve Reduction of HIV in African Men) translates to “path” or “way” in Chichewa, the national language of Malawi. The name was developed in coordination with our local Community Advisory Board and reflects the study’s objective of examining how best to support men in navigating the complex path toward sustained and effective HIV prevention. The primary objectives of this study are to assess the effect of a systems navigator–facilitated HIV prevention package on PrEP persistence among heterosexual men seeking STI clinical services in Lilongwe, Malawi at 26 weeks, and to evaluate the acceptability of, and barriers to, implementing a systems navigator–delivered HIV prevention package among key clinic stakeholders and heterosexual men initiating PrEP at STI clinics. Implementation outcomes are feasibility, acceptability, and cost. Exploratory objectives aim to characterize men’s preferences for PrEP modalities, perceived and experienced stigma, and the influence of gender norms on PrEP persistence. The overarching hypothesis is that a peer-delivered systems navigation package will improve PrEP persistence at 26 weeks (defined as adherence to any PrEP modality through 26 weeks) among heterosexual men initiating PrEP at an STI clinic, compared with the current standard of care (SOC).

## Methods

### Study Design

#### Overview

This study is a single-site, pilot type I effectiveness-implementation hybrid randomized controlled trial (RCT). The overall goal is to evaluate the benefits, acceptability, barriers, and associated costs of integrating systems navigation and brief counseling into STI clinic–based PrEP provision for heterosexual men in Lilongwe, Malawi (Textbox 1).

**Figure 1:**
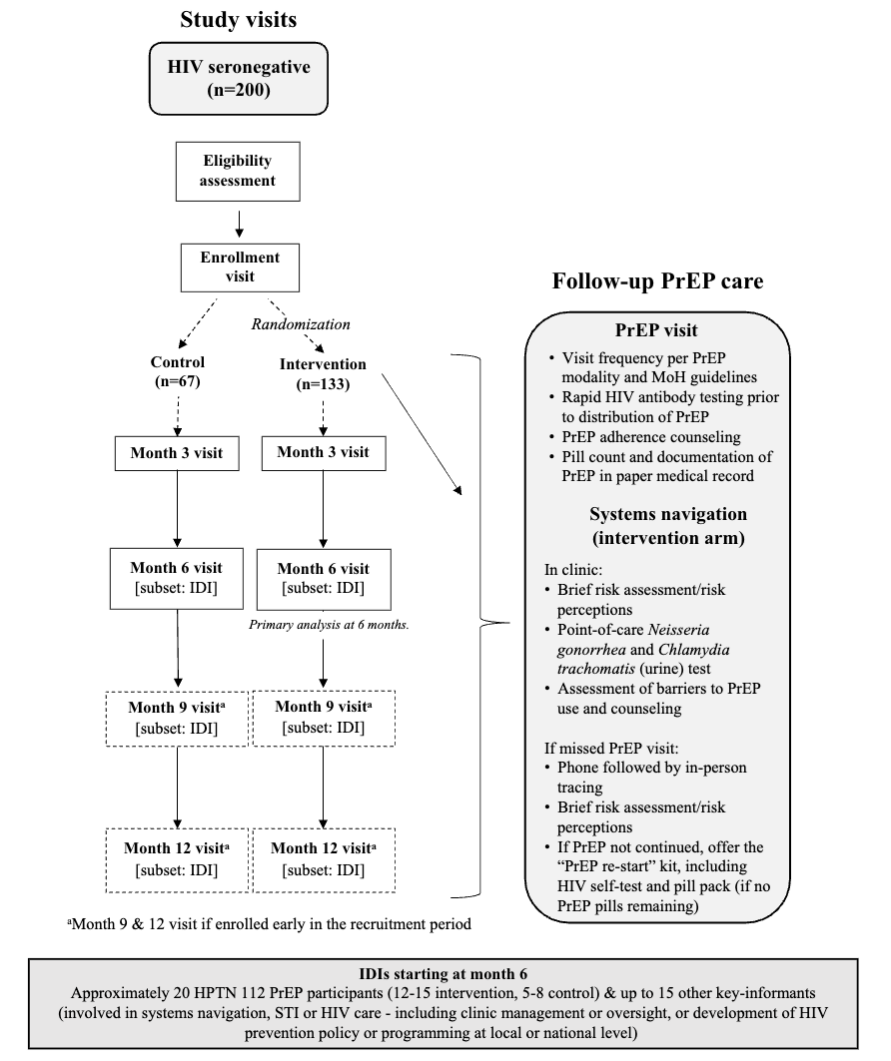
HIV seronegative men are randomized after enrollment to either control or intervention conditions, with quarterly follow-up thereafter. Follow-up preexposure prophylaxis (PrEP) care and exposure to systems navigation are separate from study follow-up and proceed according to the PrEP follow-up schedule per Malawi PrEP guidelines. A subset of enrolled participants (approximately 20), along with additional key informants who are not PrEP-initiator participants (approximately 15), will be interviewed to evaluate implementation outcomes of interest. IDI: in-depth interview; MoH: Ministry of Health.

Two hundred eligible participants will be randomized (1:2) to SOC PrEP services or systems navigator–assisted PrEP care (intervention). Participants will be followed for at least 26 weeks and up to 52 weeks, depending on their enrollment window. All clinical services, including PrEP provision and safety monitoring, are conducted by the Malawi Ministry of Health PrEP nursing staff (Figure 1).

**Primary, secondary, and exploratory objectives.**

**1. Primary objectives**

- To assess the effect of a systems navigator–facilitated HIV prevention package on preexposure prophylaxis (PrEP) persistence among heterosexual men seeking sexually transmitted infection (STI) clinical services in Lilongwe, Malawi at 26 weeks.
- To assess acceptability and barriers to implementing a systems navigator–delivered HIV prevention package among key stakeholders in the clinic and heterosexual men initiating PrEP at STI clinics.

**2. Secondary objective**

- To assess the feasibility of a future randomized controlled trial.

**3. Exploratory objectives**

- To assess prevention-effective PrEP use among heterosexual men initiating PrEP at STI clinics.
- To assess PrEP modality preferences among heterosexual men initiating PrEP at STI clinics.
- To assess the effect of a systems navigator–facilitated HIV prevention package on PrEP persistence among heterosexual men seeking STI clinical services in Lilongwe, Malawi at 39 and 52 weeks.
- To quantify costs and resources necessary to develop and integrate a systems navigator–delivered HIV prevention package into an urban STI clinic, informing future cost-effectiveness model development.
- To perform laboratory assessments that may include evaluation of factors related to HIV acquisition or other STIs; characterization of HIV in participants who acquire HIV; characterization of the host response to antiretroviral drugs; and evaluation of virologic, pharmacologic, or STI-based laboratory assays.
- To evaluate event-driven PrEP drug concentrations in the context of timeline followback–reported PrEP use and sex acts.
- To explore the perceived and experienced PrEP-related stigma and potential influence of perceived gender norms on PrEP persistence among heterosexual men initiating PrEP at the STI clinic.

#### Systems Navigation Intervention

Systems navigation and counseling is a theory-derived, evidence-based intervention that involves identifying persons most likely to benefit from HIV prevention tools and helping them navigate complex, dynamic obstacles to uptake and retention. This intervention draws on a combination of social cognitive theory and social identity theory, the latter helping to explain how individuals conceptualize themselves in relation to social groups, including gender and sexual preferences. In this study, systems navigation uses motivational interviewing to build a person’s capacity and confidence to remain engaged in PrEP care. The systems navigator manual was adapted from prior intervention studies, namely, HPTN 074 [33] (conducted among persons who inject drugs living with HIV) and HPTN 082 [34] (conducted among adolescent girls and young women not living with HIV), in which peer-adjacent persons were deployed to assist with HIV treatment or HIV prevention service delivery. The navigator manual was adapted to align with the local context and the study-specific objectives of engaging heterosexual men who had initiated PrEP. This manual consists of self-guided content specific to the study, as well as modules and activities that serve as the backbone for all participant sessions (Multimedia Appendix 1).

The systems navigation intervention package includes 3 components as described in Textbox 2.

**Components of the systems navigation intervention package.**

**1. Access to a systems navigator**

Intended to engage with participants at each preexposure prophylaxis (PrEP) visit using a menu of “sessions” that examine items such as risk perception and address barriers to PrEP adherence (Multimedia Appendix 1); provide reminders for upcoming PrEP visits; and trace participants who have missed a scheduled PrEP visit via phone or in person.

**2. Point of care sexually transmitted infection testing**

Urogenital testing for *Chlamydia trachomatis* and *Neisseria gonorrhea* via urine sample.

**3. PrEP reengagement or “restart” kit**

Consists of an HIV self-test kit and oral PrEP pills for reinitiation. The “restart” kit is offered to participants who choose to stop PrEP (oral or injectable), alongside counseling regarding safely restarting PrEP as a bridge to reestablish in-person PrEP care at the clinic.

Systems navigators are selected from a cadre of health care workers with some counseling experience but without formal clinical training. Familiarity with HIV testing or prevention services is recommended, though navigators are not required to have personal experience using PrEP. The navigation role provides both behavioral counseling and assistance in navigating the clinical services of the PrEP clinic.

Navigators are men selected from the community with guidance from the Community Advisory Board. They must be knowledgeable about the community’s dynamics and reflect the demographics of the target population. Navigators will undergo extensive training, including but not limited to basic knowledge of STIs, HIV, and PrEP; PrEP modalities, including potential benefits and expected challenges; motivational interviewing; psychosocial counseling; techniques for active listening and rapport building; HPTN 112 procedures, including urine specimen collection and handling (for STI testing); and documentation to track participant engagement. As this protocol is still considered research, all navigators will receive training related to the ethical conduct of clinical research (ie, good clinical practice). Navigators are integrated into STI/PrEP clinic procedures but, for this protocol, are hired study staff within the study team.

Although not required, navigators may have been previously or currently prescribed PrEP. They will also be trained on personal safety, discreet tactics for follow-up tracing, and confidentiality. These components and attributes have been developed in coordination with local Community Advisory Board members. All training materials will be prepared in advance of study implementation and compiled into a separate systems navigation manual, which will include a series of didactics, role-playing, and direct observation. Navigators will receive periodic refresher training and regular evaluations. They engage with participants during PrEP visits and in the field to trace participants who have missed a PrEP visit, activities that are intentionally distinguished from study visits.

All navigators participate in regular (eg, weekly) debrief sessions, during which they are encouraged to reflect on recent engagement with participants—considering what worked well and where they encountered challenges. Feedback from these sessions is shared with study leadership to help inform additional training or updates to available tools for participant engagement. Additionally, coordinators review expected PrEP visits and anticipated navigator contact with participants. Gaps in contact attempts or missed outreach (ie, no tracing for missed PrEP visits) are communicated to navigators, and barriers to completing tasks are discussed. This audit-and-feedback [35,36] approach facilitates rapid intervention to address any knowledge or implementation gaps.

Navigators are intended to be integrated within PrEP service delivery at the STI clinic, working alongside Ministry of Health PrEP nurses to engage male participants randomized to receive the intervention. As such, navigation activities occur primarily at the Bwaila STI Clinic, except for tracing, which takes place in the field. Men in the intervention arm of the study will also be exposed to any SOC activities related to PrEP service delivery (Table 1).

**Table 1:** Standard of care compared with study intervention components.

Component	Standard of care	Intervention^a^
HIV testing (all PrEP^b^ visits)	Serial third-generation rapid antibody	Serial third-generation rapid antibody
STI^c^ testing (PrEP follow-up)	Not standard: symptom-based screening	Point-of-care: *Neisseria gonorrhea* and *Chlamydia trachomatis* (urine)
Visit reminders (PrEP follow-up)	Not standard	Per participant preference
Assessment of barriers to PrEP use (all PrEP visits)	PrEP nurse delivered	Systems navigator via motivational interviewing
Adherence counseling (PrEP follow-up)	PrEP nurse delivered	Systems navigator via motivational interviewing
Tracing for missed PrEP visits (PrEP follow-up)	Not standard	Per participant preference (phone or in-person)
PrEP reengagement counseling (PrEP follow-up)	Not standard	PrEP “restart” kit offered

^a^Participants randomized to the intervention condition *also* receive all standard-of-care PrEP services.

^b^PrEP: preexposure prophylaxis.

^c^STI: sexually transmitted infection.

#### Standard of Care

Men randomized to the SOC condition will receive Malawi’s SOC for PrEP services. SOC includes serial third-generation rapid HIV antibody testing before PrEP initiation or refill, an assessment of PrEP need and risk of HIV acquisition, and nurse-provided counseling regarding PrEP adherence and continuation. There is no standard practice for tracing persons who have missed PrEP visits. PrEP counseling is consistent with contemporary guidelines and encourages persons to remain engaged in PrEP care as long as they remain vulnerable to acquiring HIV.

### Study Setting

#### Bwaila STI Clinic

The Bwaila STI Clinic is located within Bwaila District Hospital, a tertiary care hospital in urban Lilongwe, Malawi. PrEP services have been colocated within the STI clinic since November 2021. Per Malawi guidelines, all persons presenting with symptoms of an STI are managed syndromically, and all persons who are not known to be living with HIV are offered HIV testing with serial third-generation rapid antibody tests. Generally, the STI clinic sees about 50 HIV seronegative patients per day, approximately half of whom are men. Per Malawi guidelines, all persons with a recent STI may be eligible for PrEP.

#### PrEP in Malawi

According to the most recent PrEP guidelines for Malawi, persons are eligible for PrEP if they are HIV seronegative; at substantial risk for HIV, with prioritization of persons who buy or sell sex, are STI clients, or are within sero-different couples, including HIV-negative individuals whose partner living with HIV is not on antiretroviral therapy (ART), has been on ART for less than 6 months, has a high viral load, or is nonadherent to ART; have no concern for acute HIV based on provider screening for signs or symptoms consistent with acute HIV; have expressed willingness to attend scheduled PrEP visits; have no contraindications to PrEP drugs; have a bodyweight ≥30 kg; have no known renal disease; and are not known to have diabetes mellitus.

As of April 2024, Malawi PrEP guidelines included the following biomedical PrEP modalities: daily oral PrEP with tenofovir disoproxil fumarate/lamivudine (TDF/3TC) or TDF/emtricitabine (TDF/FTC) for men and women; event-driven PrEP (TDF/3TC or TDF/FTC) for men only; and injectable PrEP (CAB-LA) for men and women, available under limited-use agreements with implementing partners and external funders. At Bwaila STI Clinic, during the study period, all 3 PrEP modalities are available to men initiating PrEP after presenting with symptoms of an STI.

Study investigators are not providing PrEP services; all PrEP care, including eligibility assessment and clinical monitoring, is conducted by the Malawi Ministry of Health PrEP nurses.

### Participant Eligibility

Two populations are eligible to participate in the study: men initiating PrEP after seeking STI services (n=200), and relevant stakeholders involved in the adoption or implementation of PrEP program delivery in Malawi (approximately 15), who will participate in in-depth interviews (Multimedia Appendix 2).

Eligibility criteria for inclusion in this study as a participant initiating PrEP are (1) men; (2) aged 18 years or older, or 15-17 years (with assent and parent/guardian consent); (3) planning to remain in the study area for at least 26 weeks after enrollment; (4) willing to provide a phone number or address to facilitate tracing in the event of a missed study visit; (5) willing to participate in all study activities, including specimen collection (blood and urine) and intervention if randomized to that condition; (6) have sought STI clinic services and initiated PrEP within 7 days of enrollment; (7) self-identify as heterosexual and report at least one female sex partner in the 6 months before enrollment; and (8) able to provide informed consent. Persons who have used PrEP previously are eligible if at least 13 weeks have elapsed since their last PrEP use (pill or injection; Multimedia Appendix 3).

All participants will have been prescribed PrEP before enrollment. As such, they will have been deemed PrEP-eligible according to contemporary Malawi PrEP guidelines (see above). Key stakeholders relevant to adoption are eligible to participate in in-depth interviews if they are aged 18 years or older; involved in the provision of clinical service navigation, systems navigation, STI or HIV care or prevention (including clinic management or oversight); or involved in the development of HIV prevention policy or programs at the local or national level; and able to provide informed consent.

Participants who appear to have psychological instability or cognitive impairment that would limit their ability to understand study procedures, or any condition that, as determined by the investigators, would make participation unsafe or otherwise interfere with the conduct of the study, will be excluded. For PrEP user participants, current participation in any HIV prevention study or other study considered likely to interfere with the interpretation of study outcomes will also lead to exclusion.

### Study Procedures

#### Data Collection

Data sources and collection methods are described below. The study will utilize electronic data capture tools within the Research Electronic Data Capture (REDCap; Vanderbilt University) database for all primary data collection and participant management purposes. REDCap is a secure, web-based software platform designed to support data capture, auditing, monitoring, and export for research studies. Study-specific assessments will be conducted separately from STI clinic–based PrEP visits. Although study visits and PrEP care can occur on the same day, study visits take place in a separate building. This distinction is intentional, designed to minimize the impact of study-specific compensation, which covers time and transportation expenses, on the primary effectiveness outcome of persistent PrEP use.

Per Malawi PrEP guidelines, persons on daily or event-driven oral PrEP (TDF/3TC or TDF/FTC) have follow-up visits at 1 month, 3 months, and quarterly thereafter. Persons on injectable PrEP (long-acting cabotegravir) have follow-up visits at 1 month, 3 months, and every 2 months thereafter. The study will track how often study visits and PrEP visits occur on the same day. Participants are reminded that remaining on PrEP is not required to stay in the study. Standard pre- and posttest counseling will be provided during HIV testing at each study visit, and all participants will be offered condoms free of charge.

#### Recruitment

**Figure 2:**
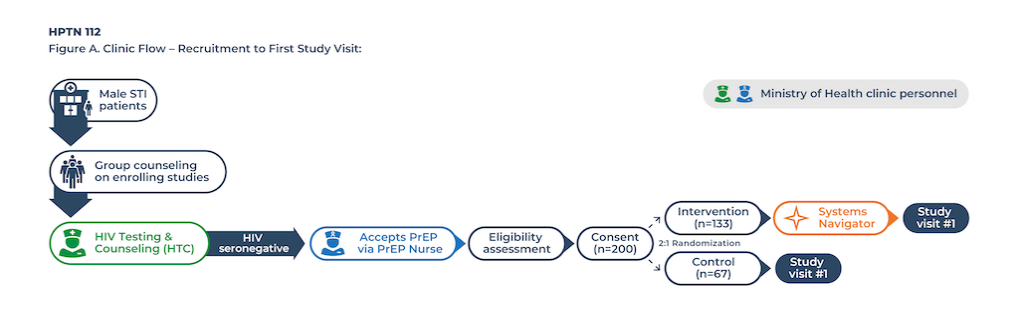
Male sexually transmitted infection (STI) clinic patients proceed through standard clinic procedures, beginning with group counseling and health education talks, which include an introduction to enrolling studies at the clinic. All persons presenting for STI services receive HIV testing and counseling. Individuals who test seronegative on rapid diagnostic tests are referred for further evaluation for preexposure prophylaxis (PrEP) by the Ministry of Health PrEP nurse. Men who accept PrEP are then referred for additional study eligibility assessment. If deemed eligible and interested in participating, they are consented and randomized to 1 of the 2 study arms. Those randomized to the intervention condition will engage with the systems navigator, typically on the same day as enrollment activities.

**Figure 3:**
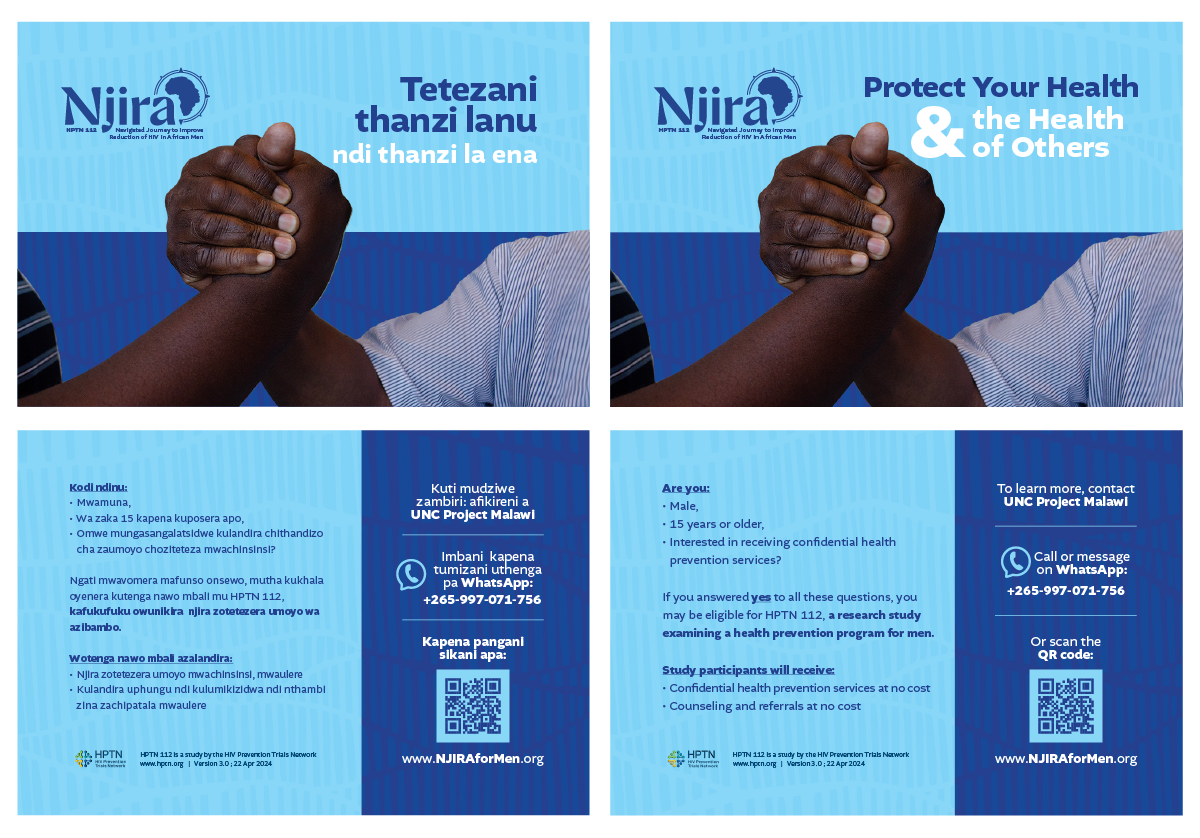
Potential participants (namely, men seeking sexually transmitted infection services at the recruiting site) may be handed palm cards describing the Navigated Journey to Improve Reduction of HIV in African Men (NJIRA) study. The front of the card features an image of 2 hands meeting, while the back provides basic eligibility information for study participation, including contact details for those interested in learning more, such as a study-dedicated phone number and a study-specific web page. Panel (A) presents this information in English, and (B) in the local language, Chichewa, which is the version that is distributed. The same images and content are also printed on larger-format posters displayed throughout clinic waiting areas and preexposure prophylaxis rooms.

PrEP user participants are recruited from the Bwaila STI Clinic (Figure 2). All individuals seeking STI clinic services receive a group health talk during which the HPTN 112 study is introduced. Ministry of Health PrEP nurses are also briefed about the study and can refer any male initiating PrEP for further enrollment consideration. Palm cards and posters (see Figure 3 [37]) are available for distribution and displayed prominently throughout the STI clinic.

#### Randomization

Randomization will be stratified by age groups (15-25 and >25 years), using a permuted block design to ensure balanced treatment assignments within each stratum. Age-based stratification was chosen due to anticipated differences in the intervention’s effect and expected PrEP persistence between younger and older men [24,38]. A 2:1 randomization ratio is used to facilitate the evaluation of acceptability outcomes, which are assessed only among participants assigned to the intervention arm. Neither participants nor study personnel will be blinded to treatment assignment, as masking is not feasible given the nature of the intervention and the arm-specific data collection procedures.

#### Enrollment Visit

Participants may enroll up to 7 days after initiating PrEP. Enrollment is completed once all informed consent procedures are finalized, including randomization. All enrolled participants will have undergone HIV antibody testing as part of the local PrEP eligibility assessment; however, specimens for antibody testing will also be collected on the day of enrollment (see Table 2). Additionally, all participants will be screened for acute HIV infection using HIV RNA testing. A detectable HIV RNA result combined with negative or discordant serial HIV antibody tests will be considered indicative of acute HIV infection. Participants with any detectable HIV RNA at enrollment will be promptly linked to appropriate HIV care.

**Table 2:** Schedule of evaluations for participants not living with HIV.

Schedule		Eligibility determination	Enrollment	Weeks					Early termination^a^		
				13	26	39^b^	52^b^				
**Administrative and Behavioral Evaluations/procedures^c^**											
	Informed consent	N/A^d^	✓	N/A	N/A	N/A	N/A	N/A			
	Randomization	N/A	✓	N/A	N/A	N/A	N/A	N/A			
	Demographics	✓	✓	✓	✓	✓	✓	✓			
	HIV risk factors	✓	✓	✓	✓	✓	✓	✓			
	Locator information	N/A	✓	✓	✓	✓	✓	N/A			
	Behavioral risk assessment	N/A	✓	✓	✓	✓	✓	✓			
	Preexposure prophylaxis use assessment	N/A	✓	✓	✓	✓	✓	✓			
	Mental health status	N/A	✓	✓	✓	✓	✓	✓			
	Substance use	N/A	✓	✓	✓	✓	✓	✓			
	Perceived HIV and preexposure prophylaxis stigma	N/A	✓	✓	✓	✓	✓	✓			
	Gender norms	N/A	✓	✓	✓	✓	✓	✓			
	HIV risk reduction counseling	N/A	✓	✓	✓	✓	✓	✓			
	In-depth interviews^e^	N/A	N/A	N/A	✓	✓	✓	N/A			
**Clinical evaluations/procedures**											
	Symptom-driven physical examination	N/A	✓	✓	✓	✓	✓	N/A			
	Venous blood draw	N/A	✓	✓	✓	✓	✓	✓			
	Urine collection	N/A	✓	✓	✓	✓	✓	✓			
	Hair collection	N/A	N/A	✓	✓	✓	✓	✓			
	Sexually transmitted infection management referral^f^	N/A	✓	✓	✓	✓	✓	✓			
**Laboratory evaluations/procedures**											
	Rapid HIV testing	✓^g^	✓^g^	✓	✓	✓	✓	✓			
	HIV RNA^h^	N/A	✓	N/A	N/A	N/A	N/A	N/A			
	Sexually transmitted infection testing^i^	N/A	✓	✓	✓	✓	✓	✓			
	Plasma (storage)	N/A	✓	✓	✓	✓	✓	✓			
	Dried blood spot (storage)	N/A	✓	✓	✓	✓	✓	✓			
	Urine (storage)	N/A	✓	✓	✓	✓	✓	✓			
	Hair (storage)	N/A	N/A	✓	✓	✓	✓	✓			

^a^Extent of procedures, including surveys and specimen collection, at early termination visit will be determined depending on reasons for termination and ongoing safety/ability to obtain the consent of the participant.

^b^All participants will be followed at least 26 weeks; persons enrolled in the first 13 weeks will be followed up to 52 weeks.

^c^Systems navigation occurs at the participant’s clinic preexposure prophylaxis visits, and not at the study visits for this study. Therefore, systems navigation provided by the study is not included in this schedule of evaluations.

^d^N/A: not applicable.

^e^In-depth interviews will be conducted with a subset of participants at their final visit (week 26, week 39, or week 52).

^f^Referral for management in the event of new or persistent sexually transmitted infection symptoms/complaints.

^g^HIV testing for preexposure prophylaxis eligibility determination will be conducted by Malawi Ministry of Health personnel.

^h^HIV RNA will be tested at the local laboratory at enrollment; HIV RNA testing may be performed on stored plasma collected at follow-up visits at the HPTN Laboratory Center.

^i^Sexually transmitted infection testing, including *Neisseria gonorrhea* nucleic acid amplification test, *Chlamydia trachomatis* (nucleic acid amplification test), and syphilis (rapid plasma reagin) will be collected at study visits *unless* results of these tests are documented within 2 weeks in the review of record. This would occur in the intervention arm only, but there will not be any syphilis testing done by systems navigators (they only collect urine for *N. gonorrhea* and *C. trachomatis*).

All participants will complete study staff-administered surveys using validated scales whenever possible, enabling exploration of predictors of PrEP persistence and informing targeted objectives and strategies to enhance systems navigation and counseling interventions. To gather more detailed information on sexual activity—particularly important for men opting for event-driven oral PrEP—we will use a timeline followback calendar to capture self-reported sexual acts and PrEP use over the preceding 30 days.

After providing informed consent (by signature or fingerprint), all participants are asked to provide locator information to facilitate tracing in the event of a missed study visit. Staff-administered surveys assess sexual behaviors, perceived vulnerability to HIV, and baseline health factors including substance use [39], mental health [40-42], perceived gender norms [43-45], anticipated or experienced HIV [46], and PrEP-related stigma [47].

Participants reporting symptoms of an STI will undergo a symptom-directed physical examination. All participants will provide blood and urine samples for protocol-specified testing, which includes rapid HIV antibody testing, HIV RNA testing, urogenital *C. trachomatis*/*Neisseria gonorrhoeae* testing, and syphilis screening using rapid plasma reagin with reflex *Treponema* pallidum particle agglutination assay if indicated. Plasma, urine, and dried blood spots (DBS) will be collected and stored for batch shipment to a central laboratory for additional testing (see Table 2).

#### Follow-Up Visits

Participants will be followed quarterly (every 13 weeks) for a minimum of 26 weeks. Those enrolled within the first 13 weeks of recruitment will be followed for 52 weeks, while participants enrolled during the first 26 weeks will be followed for 39 weeks. All other participants will have a total follow-up duration of 26 weeks. The 26-week follow-up period was selected to assess PrEP persistence during the window of presumed indication, specifically reflecting the recommendation that PrEP is appropriate for individuals presenting with STI symptoms or who had an STI in the prior 6 months. This timeline aligns with prior studies evaluating PrEP persistence in the region [34], facilitating comparison with other persistence-focused studies globally, which serve as important benchmarks for clinical outcomes [38]. Following a subset of participants for up to 52 weeks captures anticipated fluctuations in perceived PrEP need and usage, providing valuable insights into PrEP service delivery among individuals with ongoing HIV vulnerability. Each follow-up visit includes a study nurse–administered survey, mirroring the enrollment survey but with additional questions specific to PrEP use during the preceding interval. Participants randomized to the intervention arm will complete additional survey questions focused on their engagement with the systems navigators, including frequency of contact, preferences, and acceptability measures [48-50]. A subset of these participants will be invited to participate in an in-depth interview during their study visits.

As at the enrollment visit, participants will undergo a symptom-directed physical examination and provide blood and urine samples for protocol-specified testing. Per protocol version 2.2 (letter of amendment #2, dated June 28, 2024), participants will also be asked to provide a scalp hair sample at follow-up visits. HIV RNA testing will not be performed routinely at each follow-up but will be reflexively conducted if HIV antibody testing is reactive. Plasma, urine, DBS, and hair specimens will be stored for batch shipment to a central laboratory for further analyses (see Table 2).

#### Procedures for Participants With Suspected or Confirmed HIV

Any participant with a reactive or positive HIV test during follow-up will undergo additional testing to confirm their HIV status. Confirmation of HIV infection requires positive test results from samples collected on 2 separate occasions. All confirmed cases will be referred to HIV care. Participants confirmed to be living with HIV will remain in the study for up to 26 weeks following seroconversion to allow for further testing and to confirm viral suppression on ART (see Table 3).

**Table 3:** Schedule of evaluations for participants who seroconvert/are identified as living with HIV.

Schedule		Weeks after seroconversion^a^			Early termination		
		13 weeks	26 weeks				
**Administrative and behavioral evaluations/procedures**							
	Locator information	✓	✓	N/A^b^			
	Antiretroviral therapy use	✓	✓	✓			
	Perceived HIV stigma	✓	✓	✓			
**Clinical evaluations/procedures**							
	Venous blood draw	✓	✓	✓			
	HIV management referral^c^	✓	✓	✓			
**Laboratory evaluations/procedures**							
	HIV RNA	✓	✓	✓			
	Plasma (storage)	✓	✓	✓			
	Dried blood spot (storage)	✓	✓	✓			

^a^Persons who are identified as acutely infected (ie, negative HIV rapid antibody test but detectable HIV RNA) at enrollment visit will be followed along the same schedule as those persons who seroconvert during study follow-up.

^b^N/A: not applicable.

^c^HIV management referral will only occur if the participant is not already in care.

Participants with detectable HIV RNA at the enrollment visit (indicating acute HIV infection) will be linked to appropriate HIV care and followed at weeks 13 and 26 to assess viral suppression.

#### Visit Windows

For each required study visit, an allowable visit window specifies the study days (after enrollment) during which the visit may be completed. These allowable windows are contiguous between visits and do not overlap. Within each allowable visit window, there is a target visit window during which study visits should ideally be conducted. If all visit procedures cannot be completed in a single day, they may be spread across multiple days within the allowable visit window.

#### Qualitative Data Collection

**Figure 4:**
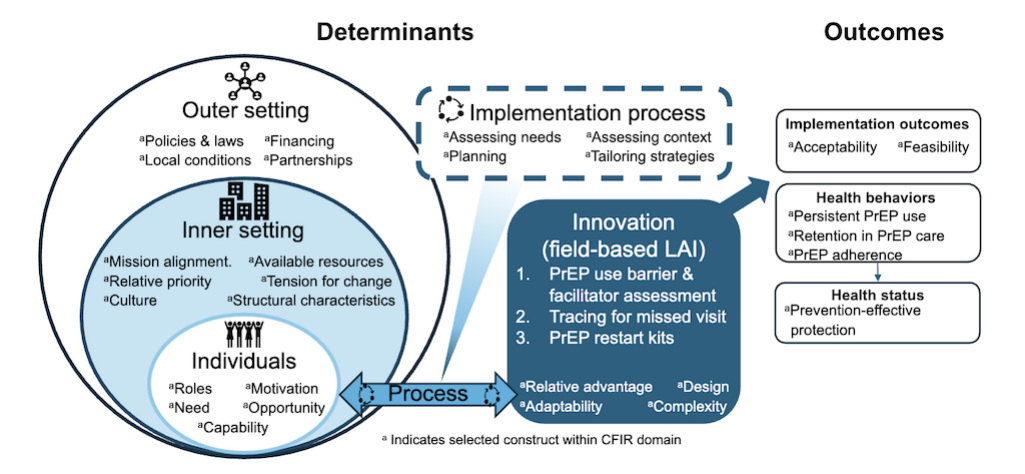
In-depth interview guides are developed using the Consolidated Framework for Implementation Research (CFIR) to examine determinants within the outer setting, inner setting, individual, process, and innovation domains that may influence our implementation, behavioral, and health outcomes of interest. Selected constructs within the CFIR domains are highlighted based on their anticipated relevance for key informant interviews. PrEP: preexposure prophylaxis.

To assess the acceptability of the intervention, individual semistructured interviews will be conducted with stakeholders involved in PrEP program implementation and scale-up, as well as with enrolled PrEP user participants. Interview guides have been developed to capture key implementation outcomes, using the Consolidated Framework for Implementation Research (CFIR) as a foundation [51,52]. Specifically, the study examines determinants and their relationships to implementation, behavioral, and health outcomes, guided by CFIR domains and selected constructs (Figure 4).

For PrEP users, willingness to participate in in-depth interviews is documented during enrollment via consent forms. We will use a purposive sampling strategy designed to capture a broad range of perspectives related to the phenomenon under study. This approach facilitates careful consideration of range, saturation (redundancy), and stratification within the sampling frame.

Additional key stakeholder participants will be identified based on their roles and responsibilities within PrEP programming at the Bwaila clinic and more broadly across Malawi, as outlined previously (see the discussion on eligibility criteria above).

Semistructured interviews will follow a standardized guide, incorporating probes tailored to the perspectives of stakeholders. These interviews will explore participants’ experiences with the intervention, perceived barriers to sustained PrEP engagement among the target population, and potential obstacles or facilitators to scaling and sustaining the intervention. Specifically, they will examine how the intervention addresses barriers or enhances facilitators of PrEP uptake and continued effective use. Conducted by a trained study interviewer, who is a native Chichewa speaker with extensive experience conducting interviews at Bwaila Hospital, each session will last approximately 60 minutes and take place in a setting that ensures participant privacy and confidentiality. During data collection with both key stakeholders and participants, a subset of interview recordings will be translated and transcribed for review by the qualitative analysis team. The team will discuss the transcripts and suggest revisions to the interview guide. This iterative process will continue until a consensus is reached on the guide’s structure.

All interviews will be audio-recorded, then translated and transcribed by qualified personnel. Identifiable information will be redacted from transcripts before they are shared for analysis.

#### Cost and Resource Data Collection

We will embed empirical costing into the study procedures, collecting cost data prospectively through 2 methods: micro-costing and time-and-motion logs. Micro-costing uses a direct enumeration, ingredients-based approach to quantify all resources consumed in developing and implementing the intervention. Additionally, we will extract data from project expenditure and management records, including purchase logs and human resource documentation. Time-and-motion assessments will capture how navigators allocate their time across navigation-related tasks, such as training, counseling, tracing, and other activities, enabling precise attribution of effort toward the intervention’s implementation. No formal economic modeling is planned as part of this protocol.

### Statistical Considerations

#### Sample Size

For the approximately 200 heterosexual men we expect to enroll, we anticipate a 10% loss to follow-up by 26 weeks. As a pilot study, the sample size is selected to provide reasonable precision in assessing the primary end points and to inform future research. We chose a 2:1 randomization ratio favoring the intervention arm over SOC to maximize our ability to evaluate the intervention’s acceptability. Based on prior studies, we expect persistent PrEP use under SOC to be approximately 40% at 26 weeks. We aim to detect a difference in persistent PrEP use between the intervention and SOC arms ranging from 15% to 35% at 26 weeks. Our sample size, chosen to enable rapid enrollment and address this important and timely knowledge gap, will be sufficient to detect a minimum regression discontinuity of 0.22 with 80% power at a 5% alpha level, and a regression discontinuity of 0.25 with 90% power at a 5% alpha level.

#### Statistical Analysis

##### Effect of Intervention on Persistent PrEP Use

A comparison of persistent PrEP use will be conducted for all enrolled participants during the first 26 weeks of study follow-up. Participants may choose to switch PrEP modalities during follow-up; persistence with each modality will be assessed during the period in which the participant reports using that modality, based on a combination of self-report, measured drug levels (for oral PrEP), and clinic records. Specifically, persistent PrEP use is defined as adherence to any PrEP modality through 26 weeks, with adherence defined as follows: for long-acting injectable PrEP, receiving on-time injections (within 7 days before or after the target injection date for the first injection and within 14 days before or after the target injection date thereafter); for daily oral PrEP, having protective PrEP concentrations detected at designated study follow-up visits, based on intraerythrocytic tenofovir diphosphate (TFV-DP) levels collected via DBS, with TFV-DP concentrations corresponding to 4 or more doses per week classified as adherent; for event-driven PrEP, self-reported adherence to the 2+1+1 dosing regimen in the past 30 days, assessed through self-reports of PrEP use and sex acts at study follow-up visits, along with intraerythrocytic TFV-DP concentrations. Only participants retained in the study at the end of 26 weeks will be considered to have reached the persistence end point. Data will be presented in a 2-by-2 table, classifying each participant as either persistent or nonpersistent to PrEP. A *Z*-test based on asymptotic normality will be used to compare persistent PrEP use between the intervention and SOC arms.

##### Acceptability and Barriers

For binary end points, frequencies will be tabulated, and proportions will be calculated along with variances estimated under the binomial distribution. For continuous end points, the mean, median, SD, quantiles, and range will be reported.

##### Feasibility of Future RCT

Secondary data analysis will tabulate the number of each end point observed during the study. For binary end points, the percentage of total enrolled participants will be calculated, with variance estimated under the binomial distribution. For continuous end points, the mean, median, SD, quantiles, and range will be reported. *Z*-tests will be used to compare end points between study arms.

#### Qualitative Analysis

After transcription, all qualitative interviews will be translated into English as needed and uploaded into a qualitative data analysis software program. The team will follow a structured process of reading, coding, data display, and data reduction to explore participants’ attitudes toward and experiences with the intervention in greater depth. Data coding and analysis will be iterative and interactive, conducted by a team of both US- and Malawi-based qualitative researchers. All US-based team members have lived or worked in Malawi for many years, including conducting trials at the Bwaila STI Clinic. The team will begin by reading all interview transcripts to increase familiarity with the data. Next, they will apply a priori codes and generate emergent codes, using a mixed inductive and deductive approach to coding and analysis. Transcripts will then be reread to develop pattern codes that group related concepts under broader themes. Through iterative discussion and consensus among the qualitative analysis team, consistent patterns in meaning, concepts, and themes across all interviews will be identified to inform the development of the final codebook. A subset of transcripts will be coded by multiple team members to ensure intercoder reliability. Discrepancies will be discussed and resolved through consensus. Detailed memos will support the development of additional codes as needed and guide the construction of data matrices. These matrices will be organized using a combination of parent codes and emergent themes to examine how participants’ perceptions converge and diverge with respect to intervention acceptability (eg, ease of use, interactions with systems navigators, perceived efficacy), risk perception (eg, motivations for participation, impact on partners or other social relationships), and interest in the future use of a systems navigation component embedded within the clinic. Efforts will be made to elevate meaning-based, interpretive themes for matrix analysis, consistent with a reflexive thematic analysis approach [53]. Comparative analyses will also be conducted to explore potential differences across participant subgroups. Illustrative quotes will be documented within the data matrix under relevant themes to guide further discussion of response patterns.

#### Laboratory Procedures

HIV and STI testing will be performed as outlined in the schedule of events (see Tables 1 and 2). HIV antibody and RNA, urogenital *C. trachomatis*/*Neisseria gonorrhea*, and syphilis tests will be conducted locally, with results returned to participants and referrals provided for treatment as appropriate. Blood (plasma and DBS), hair, and urine specimens will be stored and batch-shipped to the HPTN Laboratory Center for additional testing. Stored specimens may be used to measure antiretroviral drugs, characterize HIV in participants who acquire HIV during study follow-up, facilitate additional testing of STI pathogens, and evaluate laboratory assays related to study objectives. Specifically, these tests are conducted to characterize factors associated with HIV and STI infection, as well as virologic, pharmacologic, and immunologic responses among participants initiating PrEP at the Bwaila STI Clinic.

### Ethics Considerations

All study materials, including the protocol, informed consent forms (ICFs), and any participant-facing materials or data collection tools, have been reviewed and approved by local institutional review boards (IRBs) and US-based institutional IRBs before study implementation. The protocol, ICFs, participant education and recruitment materials, other required documents, and any subsequent modifications will also be reviewed and approved by the ethical review bodies responsible for oversight of research conducted at the study site. Following initial review and approval, the responsible IRBs/ethics committees will review the protocol at least annually. The investigator will submit safety and progress reports to the IRBs/ethics committees at least once per year and within 3 months of study termination or completion. These reports will include the total number of participants enrolled, the number who completed the study, any changes to the research activity, and all unanticipated problems involving risks to human participants or others.

All study materials have been approved by the University of North Carolina IRB (IRB approval number 433885) and the Malawi National Health Sciences Research Committee (IRB approval number 23/10/4208).

### Confidentiality

All study-related information will be stored securely at the study site. Participant information will be kept in locked file cabinets located in areas with access restricted to study staff. All laboratory specimens, reports, study data collection forms, process documents, and administrative records will be identified by coded numbers only, to ensure participant confidentiality. Local databases will be secured with password-protected access. Forms, lists, logbooks, appointment books, and any other documents that link participant ID numbers to identifying information will be stored separately in a locked file in an area with restricted access.

### Informed Consent

Written informed consent will be obtained from each study participant by trained study staff. Study ICFs describe the purpose of the study, the procedures to be followed, and the risks and benefits of participation, in accordance with all applicable regulations. ICFs have been translated into the local language, and the accuracy of the translation has been verified through independent back-translation. Literate participants will document their consent by signing the ICF(s). Nonliterate participants will document their informed consent by marking their ICF(s) (eg, with an X, thumbprint, or other mark) in the presence of a literate third-party witness. Participants will be provided with a copy of their ICF if they are willing to receive it.

All minors (under the age of 18) will be required to provide assent along with parental consent. According to Malawi guidelines, individuals aged 15 years and above may initiate PrEP if they meet other eligibility criteria.

### Risks, Benefits, and Compensation

This trial is not expected to expose participants to unreasonable risk. Blood draws may cause discomfort, dizziness, or faintness, and may result in bruising, swelling, or infection. Participants may feel embarrassed, worried, or anxious when completing their HIV risk assessment or receiving risk reduction and PrEP counseling. They may also experience anxiety while awaiting their HIV test results. Trained counselors will be available to support participants in managing these feelings.

Study procedures also address the potential risks associated with field-based tracing for missed study or PrEP visits, conducted by study staff and systems navigators, respectively. All tracing efforts are carried out in accordance with participants’ stated preferences and locator information, which is provided and regularly updated by participants or their parents/guardians. Community sensitization and engagement with the Community Advisory Board are key components in ensuring the safety of both participants and those conducting tracing activities. Additional measures to mitigate risk to individuals conducting tracing activities are working in pairs or teams, carrying phones to facilitate communication, using flexible transportation (ie, not relying on public transport availability), and receiving extensive training on the importance of immediate withdrawal if any hostility is perceived. Any such incidents are generally reported back to the community representative.

There may be no direct benefits to participants in this study; however, participants and others may benefit in the future from information gained through this study. Specifically, the information obtained may lead to improved HIV prevention services for heterosexual men. In addition, participants will receive HIV and STI counseling and testing as part of the study process.

Participants will be compensated for their time and effort in this study, to help offset costs related to transportation for study visits and time taken off work. Site-specific reimbursement amounts will be detailed in the study ICFs and approved by all ethical review committees.

### Adverse Event Reporting

As this study involves only low-risk activities and does not include any biomedical intervention or clinical care, standard adverse event reporting and monitoring will not be undertaken. No product related to this study is being offered or administered directly by the study staff. All drugs used by participants in this study for the prevention of HIV or management of STIs have regulatory approval for these purposes in Malawi and have well-established safety profiles. All safety monitoring of these approved drugs will be at the discretion of the Malawi Ministry of Health. Confidential HIV and STI surveillance reporting will be conducted in accordance with local regulations.

### Monitoring and Clinical Data Review

The study site investigators are responsible for the initial evaluation and reporting of safety information at the participant level, as well as for alerting the protocol team if unexpected concerns arise. Study participants will be provided with a 24-hour telephone number and contact information and will be instructed to contact the study clinician to report any social harm they may experience. For life-threatening events, they will also be instructed to seek immediate emergency care. Where feasible and medically appropriate, participants will be encouraged to seek evaluation at the location where the study clinician is based and to request that the clinician be contacted upon their arrival.

The study will establish a Study Monitoring Committee (SMC), which will meet via conference call approximately every 6 months. More frequent or ad hoc reviews may be conducted by the SMC as needed. The SMC may recommend stopping the trial if warranted; however, given the minimal risk of the study, no predefined stopping criteria will be developed.

### Social Impact Reporting

It is possible that participants’ involvement in the study could become known to others, potentially resulting in social harm (eg, if participants are perceived as living with HIV or at increased risk of acquiring HIV). For example, they may be treated unfairly or face challenges being accepted by their families or communities. Although self-identification as heterosexual is an inclusion criterion, it is possible that some male participants may disclose having sex with other men or transgender individuals, including naming men as recent partners. Given that homosexuality is illegal in Malawi, extensive care will be taken to preserve the confidentiality and safety of all participants. All staff, including systems navigators, will be informed of the sensitive nature of disclosed sexual activities and trained in the appropriate conduct of clinical research to ensure that all participants are treated fairly and professionally. Reporting of any participant-disclosed homosexual activity is not required in research studies.

Research staff will be trained to recognize, document, and report social impacts, and to provide referrals for counseling and social service support if necessary. Any social harm reported by a participant and judged by the Investigator of Record to be serious or unexpected will be reported to the site’s IRBs at least annually, or in accordance with their specific requirements. Participants may also benefit from the study in various ways. Therefore, both social harms and benefits related to study participation will be collected and recorded during study visits. If a participant reports social harm, study staff will make every effort to provide appropriate care and counseling, or refer the participant to suitable resources to ensure their safety. The site will provide such care and counseling in accordance with locally available resources. While maintaining participant confidentiality, study staff may engage the Community Advisory Board to explore the social context surrounding instances of social impact, to help minimize the likelihood of such occurrences.

### Dissemination

The results of the research will be disseminated through publication in peer-reviewed journals, conference presentations, and direct presentations to other interested stakeholders involved in HIV prevention, STI care provision, programming, and policy development. The protocol is available for public review on ClinicalTrials.gov (NCT06200545).

Written publication guidelines for authorship eligibility will follow the criteria recommended by the International Committee of Medical Journal Editors, including substantial contribution, participation in writing, approval of the final version, and accountability.

Lay summaries and infographics of the study findings and their implications will also be created for a general audience, including individuals receiving STI care or HIV prevention services at the recruiting sites. No participant names or other identifying information will be used in any dissemination materials, whether published or otherwise. The final deidentified data set will be maintained by the study’s primary investigators and may be shared with other researchers for secondary data analysis, following the establishment of acceptable data sharing and data use agreements, in accordance with the requirements of the US National Institutes of Health (funder/sponsor) and the University of North Carolina at Chapel Hill (grantee).

## Results

Enrollment opened on April 2, 2024, and was completed in November 2024. As of February 3, 2025, 199 participants were enrolled: 133 were randomized to the intervention arm and 66 to the standard arm. A total of 4 participants were found to be living with HIV at enrollment (3 with presumed acute HIV infection and 1 with false-negative serologic tests at the time of PrEP initiation). Qualitative interviews are ongoing; as of February 3, 2025, 4 interviews have been conducted with PrEP user participants and 9 with other key stakeholders relevant to HIV prevention programming in Malawi. Data collection and study follow-up are expected to continue through June 2025, with results to be published thereafter. Given the low-risk nature of this study, no interim analyses are planned. The NJIRA study is currently enrolling under protocol version 2.2 (June 28, 2024).

## Discussion

Efficiently and effectively addressing barriers to persistent PrEP use is critical to improving HIV prevention outcomes. Although obstacles to PrEP persistence have been extensively studied among certain priority populations, including men who have sex with men globally and young women in East and Southern Africa [38], heterosexual men in East and Southern Africa have been largely excluded from PrEP implementation trials and persistence-focused interventions. The barriers to PrEP use in this population, where health care engagement is often sporadic and health care services may not be perceived as male-centered, are not well understood. In this study, we will test the effects of a peer-delivered systems navigation package, which we hypothesize will improve PrEP persistence at 26 weeks (effectiveness) among heterosexual men initiating PrEP at an STI clinic, compared with the current SOC. We will also evaluate the acceptability of the navigation package among key stakeholders and male PrEP user participants.

Our STI clinic–based recruitment approach leverages the synergistic co-STI/HIV epidemics globally and within the region and population of interest. Prior work demonstrates the importance of engaging persons with STIs in HIV prevention [19], and the WHO identifies persons with STIs as a priority population for PrEP [54]. However, very little is known about how best to engage and retain men seeking STI services who are initiated on PrEP. Our prior work in Malawi suggests this is an important population to retain, given the frequent early discontinuation of PrEP despite persistent HIV vulnerability [24]. Peer and systems navigation emerge as appealing, evidence-based strategies to improve PrEP use in some populations but have not been tested among heterosexual men in East or Southern Africa [25,26,31].

Our single-site pilot RCT responds to the HPTN call for concepts (Fall 2022), which requested proposals focused on understanding prevention opportunities for heterosexual men living in East and Southern Africa, an important gap identified within network research in this region. We have further narrowed the population to men at increased risk of acquiring HIV, specifically those presenting to an STI clinic with symptoms consistent with an STI or recent exposure to a person with an STI.

HPTN 112 will also provide important insights into PrEP choice and persistence in the context of multiple PrEP modality options. Specifically, this study will describe the utilization of CAB-LA among heterosexual men in Malawi and use in-depth interviews to examine patient, provider, and programmer/policy maker perspectives relevant to access to CAB-LA for this population. Although CAB-LA PrEP removes the need for daily dosing, timely injections are necessary to achieve and maintain target drug concentrations. CAB-LA is anticipated to be a desirable option for men in the region [14], but this will be among the first studies to examine the persistent use of event-driven oral PrEP among heterosexual men in the region, a prevention strategy that requires the ability to predict sexual encounters [15,55,56]. Depending on the frequency of sex acts, event-driven PrEP could increase the interval between doses compared with daily oral PrEP. However, data on the use of this strategy have been mixed, and little is known about its acceptability and effective use among heterosexual men [57-62]. By triangulating the timing of self-reported sex acts, self-reported event-driven PrEP use, and PrEP drug concentrations measured via DBS at quarterly intervals (along with an exploratory evaluation of PrEP exposure via hair samples), this study will explore how these measures can be combined to better evaluate prevention-effective event-driven PrEP use among men.

The need for PrEP, whether real or perceived, fluctuates, and our prior work in this context demonstrates frequent PrEP discontinuations within the first 6 months of initiation [23,24]. This study will be the first to evaluate the acceptability and feasibility of a “return-to-PrEP” kit as a potential strategy to mitigate HIV acquisition risk for persons who choose to stop PrEP. Participants who no longer feel they need PrEP are encouraged to return to the clinic and are offered this “restart” kit, along with instructions for self-testing and use of the pill pack, to facilitate immediate protection if they are unable to plan ahead to visit the clinic before reengaging in activities that may put them at risk of acquiring HIV. Navigators also serve as a direct entry point to help reengage men in HIV prevention services.

This protocol is among the first studies to test systems navigation as a strategy to improve persistent PrEP use among heterosexual men in the region. The strengths of this study are its focus on an epidemiologically and socially important subpopulation of heterosexual men, who account for approximately half of all persons seeking STI services at the recruitment site but are rarely engaged in PrEP research. We are also uniquely positioned to examine the persistence and acceptability of multiple PrEP modalities, including injectable and event-driven oral PrEP, among heterosexual men. Finally, we have structured our data collection strategy to minimize overlap with PrEP service delivery, that is, study retention is distinct from retention in PrEP services, so compensation for study visits should not affect our assessment of PrEP persistence. However, there are important limitations. For example, due to the limited sample size, short recruitment period, and heterogeneity of determinants of persistent PrEP use across different populations, transgender persons and women are not included in this initial randomized pilot. Based on our experience in Malawi, persons who identify as gender nonbinary typically seek sexual health services at other local drop-in centers rather than at the STI clinic where this study will be conducted. As such, our effectiveness and some acceptability outcomes may not be generalizable to other populations who remain vulnerable to HIV and may also be prioritized for HIV prevention. However, robust implementation outcomes collected through study-specific case report forms and in-depth interviews will provide valuable insights into the determinants of scaling this intervention to other settings and populations.

Study results could inform program development and policies for PrEP delivery in Malawi and elsewhere in the region, with dissemination prioritized to reach key decision makers. Findings could guide a multisite, multicountry implementation science study integrating peer-delivered systems navigation into STI clinics as a strategy to recruit and retain men vulnerable to HIV acquisition in effective HIV prevention tools, including PrEP. Recognizing increasingly cyclical PrEP use patterns, navigators serve as a direct entry point to retain or reengage men in HIV prevention services.

## Appendix

Multimedia Appendix 1 Intervention manual for systems navigators.

Multimedia Appendix 2 In-depth interview guides.

Multimedia Appendix 3 Informed consent forms for all participants.

## Abbreviations

ART: antiretroviral therapy
CAB-LA: long-acting injectable cabotegravir
CFIR: Consolidated Framework for Implementation Research
DBS: dried blood spots
HPTN: HIV Prevention Trials Network
ICF: informed consent form
IRB: institutional review board
NJIRA: Navigated Journey to Improve Reduction of HIV in African Men
PrEP: preexposure prophylaxis
RCT: randomized controlled trial
REDCap: Research Electronic Data Capture
SMC: Study Monitoring Committee
SOC: standard of care
STI: sexually transmitted infection
TDF/3TC: tenofovir disoproxil fumarate/lamivudine
TFV-DP: tenofovir diphosphate
WHO: World Health Organization

## Data Availability

No data are presented herein. The fully approved study protocol is available upon request as well as posted on the HPTN website. The data sets generated during or analyzed during this study are available from the corresponding author upon reasonable request.
