# Supplementary material for: Improving HIV Prevention Among Heterosexual Men Seeking Sexually Transmitted Infection Services in Malawi: Protocol for a Type I Effectiveness-Implementation Hybrid Randomized Controlled Trial of Systems Navigator–Delivered Integrated Prevention Package (HPTN 112-NJIRA Study)
Source: JMIR Res Protoc. 2025 Jun 18;14:e72981. doi: 10.2196/72981 (PMC12223453; doi:10.2196/72981)
Supplement: Multimedia Appendix 1 [file resprot_v14i1e72981_app1.docx]

**SUPPLEMENTAL FILE S1: INTERVENTION MANUAL**

**
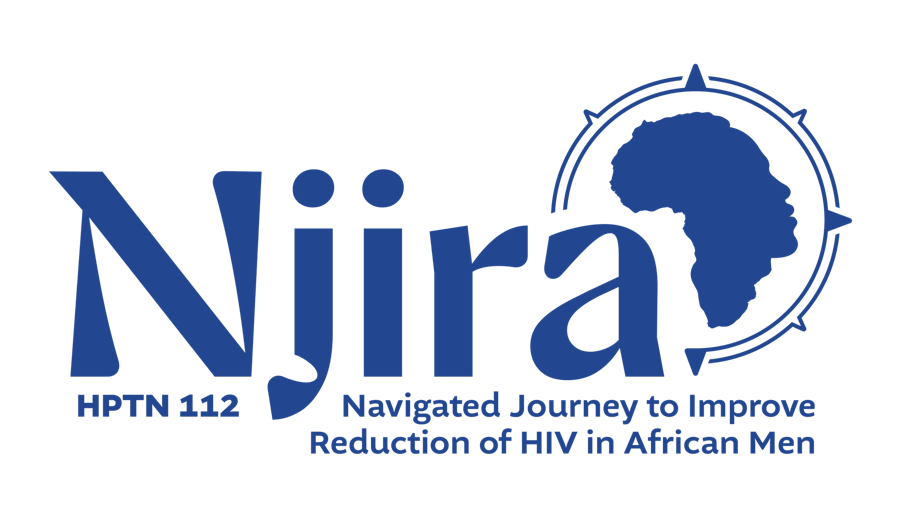
**

**Intervention Manual**

A research guide for system navigators conducting the intervention for HIV Prevention Trials Network 112

 HPTN 112

Improving HIV prevention among heterosexual cisgender men seeking STI services in Malawi: examining the benefits, acceptability, and associated costs of a systems-navigator-delivered integrated prevention package

**A Study of the HIV Prevention Trials Network**

Sponsored by:

Division of AIDS, US National Institute of Allergy and Infectious Diseases

US National Institutes of Health

The HPTN 112 Intervention Manual was largely adapted from the HPTN 074 manual, with additional modifications made by members of the HPTN 112 study team.

The content of this manual and materials used to generate this manual include:

- [The HPTN 074 Intervention Manual](https://urldefense.com/v3/__https:/www.hptn.org/sites/default/files/inline-files/HPTN074SSPAppA_Intervention*20Manual_28Sep2017_V2.3.pdf__;JQ!!ELf_LxN3sEQ!f__6witchMTeBQEN1tE-lOQpF5vZ7l2IeU7u6NxUJO1lY6oQcJBmIretA1HI54phcglhee0cxn-2h0NGde5zKXivMSJB-mdc$)
- [The HPTN 082 Intervention Manual](https://www.ncbi.nlm.nih.gov/pmc/articles/PMC8253429/)
- Resources on Motivational Interviewing provided by the UNC Center for AIDS Research

**Acknowledgements**:

The content of this manual was adapted from HPTN 074 and incorporates elements of both the HPTN 082 and SnAP study manuals. We are greatly appreciative of feedback from Hong Linh Thi Dang and Kim Ngan Nguyen, as well as the provision of Motivational Interviewing materials and training from Steven Bradley-Bull. Laura Limarzi-Klyn adapted materials from relevant sources, with additional feedback and modifications from Sarah Rutstein, erica hamilton, Mitch Matoga, and Mathews Mukatipa.


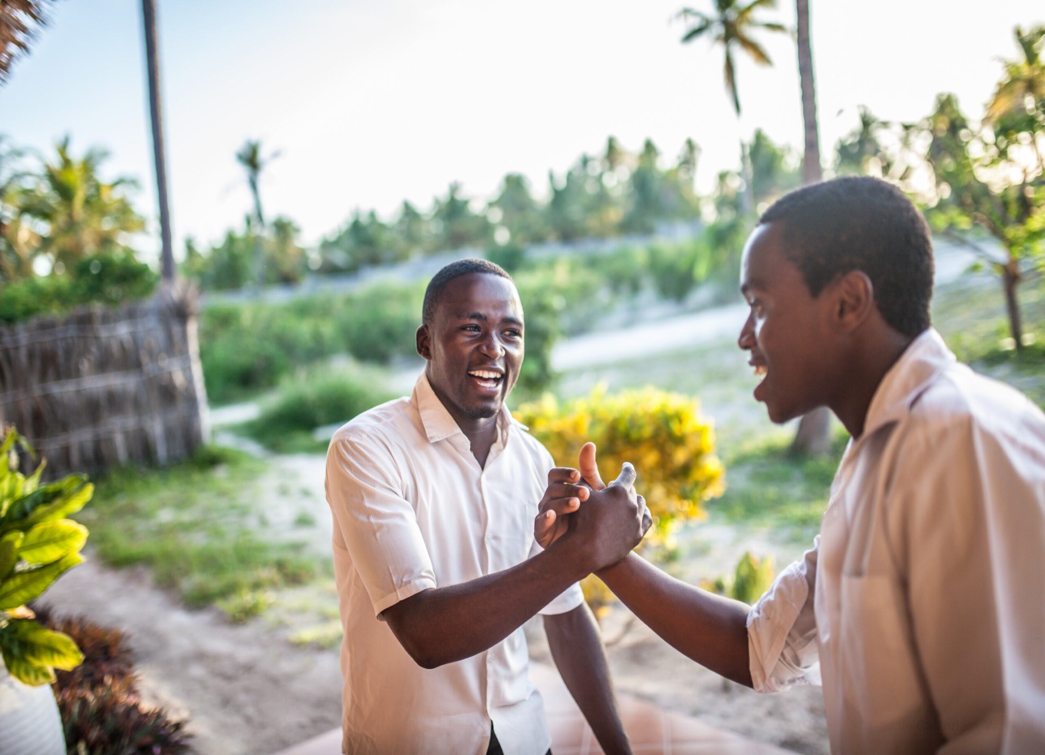


Table of Contents

[CHAPTER 1: HPTN 112 STUDY OVERVIEW AND INTERVENTION DESCRIPTION 4](#_Toc1783870723)

[A. Study design, objectives, and target population 4](#_Toc589243902)

[B. Background and science 4](#_Toc219884439)

[C. Core elements of the HPTN 112 intervention 5](#_Toc1940275076)

[D. Key elements the HPTN 112 intervention does not provide: 5](#_Toc1777392899)

[CHAPTER 2: PHILOSOPHY OF HPTN 112 INTERVENTION PROGRAM 7](#_Toc173684239)

[A. Active listening 10](#_Toc712390439)

[B. Motivational interviewing 12](#_Toc1870576598)

[C. Role-playing 19](#_Toc720164709)

[D. Cognitive behavior therapy 20](#_Toc1668553832)

[CHAPTER 4: TRAINING AND COMMUNICATION FOR IMPLEMENTATION 23](#_Toc1116930266)

[CHAPTER 5: OVERVIEW OF THE BARRIERS TO PrEP ADHERENCE 24](#_Toc1027437993)

[CHAPTER 6: STRUCTURE OF NAVIGATION SESSIONS AND MODULES 26](#_Toc485801215)

[A. Overview of sessions and modules 26](#_Toc1187525078)

[CHAPTER 7: RESPONSIBILITIES OF THE SYSTEMS NAVIGATOR 28](#_Toc828809567)

[CHAPTER 8: MODULES 32](#_Toc617199445)

[MODULE A: INTRODUCTION TO NJIRA AND GETTING TO KNOW THE PARTICIPANT 33](#_Toc1003822263)

[MODULE B: PREP OVERVIEW 38](#_Toc484811199)

[MODULE C: BARRIERS AND MOTIVATORS TO PREP ADHERENCE AND PROBLEM SOLVING 48](#_Toc633217865)

[MODULE D: PREP DISCLOSURE 54](#_Toc471025925)

[MODULE E: SEXUAL HEALTH (STI PREVENTION) 59](#_Toc962493717)

[MODULE F: CIRCUMCISION 64](#_Toc195608811)

[MODULE H: ALCOHOL USE 74](#_Toc990854511)

[MODULE I: PSYCHOSOCIAL DISTRESS AND SUPPORT 83](#_Toc1826836141)

[Chapter 9: DOCUMENTATION 90](#_Toc161195014)

[Overview 90](#_Toc537739948)

[Navigation Session Checklist 90](#_Toc331328215)

[Navigation Session Notes 91](#_Toc758441617)

[Systems Navigator Contact Case Report Form 94](#_Toc2033649336)

[Weekly Intervention Debrief Form 101](#_Toc1908695039)

# **CHAPTER 1: HPTN 112 STUDY OVERVIEW AND INTERVENTION DESCRIPTION**

HPTN 112 (NJIRA) is a pilot effectiveness-implementation hybrid type 1 trial designed to assess the feasibility and acceptability of a novel STI-clinic based intervention to facilitate rapid and continued engagement in effective HIV prevention services (PrEP), among heterosexual cisgender men. Although cisgender women in Malawi account for the majority of incident HIV, largely due to effective antenatal HIV screening, cisgender men make up a growing proportion of new infections. Failure to prevent infections among cisgender men, or rapidly diagnose and link cisgender men with prevalent infection to antiretroviral therapy (ART), fuels incident infections among cisgender women and contributes to the stagnating HIV incidence in Malawi. The STI clinic represents a unique clinical setting in which cisgender men with documented HIV risk are rarely directly engaged in effective prevention tools.

### **Study design, objectives, and target population**

The overall goal of this study is to evaluate the benefit(s), acceptability, and associated costs of integrating systems navigation and brief counseling into STI clinic-based PrEP provision. Eligible participants will be randomized (like a flip-of-a-coin) to either the intervention or standard of care groups. Only men randomized to *intervention* will receive the navigation and point-of-care STI testing services.

The primary objectives are to:

1. Assess the effect of a systems-navigator facilitated HIV prevention package on PrEP persistence among cisgender heterosexual men seeking STI clinical services in Lilongwe, Malawi at 26 weeks.
2. Assess acceptability and barriers of implementing a systems-navigator delivered HIV prevention package among key stakeholders in the clinic and heterosexual cisgender men initiating PrEP at STI clinics

Our study population includes men who are accessing STI services at the Bwaila STI clinic, aged 15 and above, who self-identify as heterosexual and who have had a female sexual partner in the last 6 months.

### **Background and science**

Despite dramatic reductions in HIV incidence globally, progress towards ending the epidemic has stalled. A vaccine has not yet been generated; evidence-based strategies include integrated biomedical and behavioral strategies. HIV status neutral interventions include effective treatment of HIV infection and provision of PrEP to people who are at high risk of acquiring HIV. The rollout of PrEP has been hampered by inconsistent referral, inadequate uptake, and poor persistence despite ongoing HIV risk. It is necessary to identify patients at high risk of acquiring HIV, efficiently link them to appropriate prevention services, while maintaining access to those services throughout the HIV risk period.

Persons seeking STI services are a compelling population on which to focus HIV prevention resources; an incident STI is an objective indicator of unprotected sex and, in high HIV incidence settings, a reasonable proxy for HIV risk. In Malawi, persons with STIs are prioritized for HIV prevention, including PrEP. Fluctuations in HIV risk and perceived HIV risk influence both the initiation of and consistent effective use of PrEP. Historically, these dynamics have been evaluated only in sero-different couples or among heterosexual cisgender women. Tailored counseling may facilitate improved PrEP uptake and use, and even brief risk-reduction counseling sessions may be effective, particularly when delivered in so-called “teachable moments”, which may include the presentation with an STI.

Systems navigation and counseling is a theory-derived evidence-based intervention that could improve referral to, uptake of, and persistence of effective HIV prevention interventions including PrEP. Systems navigation includes identifying persons most likely to benefit from HIV prevention tools and helping them navigate complex, dynamic obstacles to uptake and retention. This intervention draws from a combination of social cognitive theory and social identity theory, using counseling and engagement to develop a person’s capacity and confidence to remain engaged in PrEP care.

### **Core elements of the HPTN 112 intervention**

The intervention package, received only by participants in the intervention arm, is integrated into PrEP visits and includes:

1. Evaluation of barriers and facilitators to ongoing PrEP use.
2. Point-of-care (POC) STI testing to inform counseling regarding ongoing PrEP care engagement.
3. Tracing for any missed PrEP visits.
4. Offer of a PrEP “restart” kit for cisgender men who choose to discontinue PrEP during the follow-up period.

Recognizing increasingly cyclical PrEP use patterns, navigators serve as a direct entry point to retain or re-engage participants in HIV prevention care.

Motivational interviewing: is a key skill meant to inform the evaluation of barriers and facilitators and counseling regarding ongoing PrEP care engagement. Details of this philosophy are outlined elsewhere. Navigators are tasked with helping to engage, retain, and facilitate skills to promote adherence to PrEP care. They will use motivational interviewing, problem solving, skill building, and goal setting to assist with persistent PrEP use. Prior studies have demonstrated frequent incident STIs among person on PrEP. POC STI testing for chlamydia and gonorrhea is meant to help inform risk-reduction counseling – this testing is not the current standard of care for persons on PrEP in Malawi.

PrEP discontinuation, even with ongoing HIV risk, is common globally, including in Malawi. For persons who miss a scheduled PrEP visit, navigators are tasked with attempting to contact men using their preferred means of tracing (phone or in-person). For those who state they do not wish to re-engage in PrEP care, this intervention extends the offer of PrEP pills (if the man does not have already) and an HIV self-test, with additional instructions regarding how to safely restart PrEP and, ideally, re-engage with PrEP care prior to resumption of HIV risk behavior.

### **Key elements the HPTN 112 intervention does not provide:**

PrEP: HPTN 112 does not provide PrEP. The objective is to retain persons with ongoing HIV risk on PrEP in a manner that will protect them from acquiring HIV. HPTN 112 will not be providing PrEP, however. All PrEP care and clinical monitoring are provided by the Malawi Ministry of Health.

Personalized medical care: navigators should be integrated into the PrEP service-delivery model such that, if a side effect is noted, the participant should be referred to clinical care. Medical advice is beyond the scope of navigators and should be deferred to appropriate PrEP clinicians.

Support groups: we encourage participants to share experiences and we do offer support, but we do not organize support groups as part of this intervention. There may be situations in which participants are encouraged to engage individuals in their support network.

# **CHAPTER 2: PHILOSOPHY OF HPTN 112 INTERVENTION PROGRAM**

HPTN 112 focuses on the pathways that may lead to behavior change such as skills building, problem solving, and goal setting that address specific behaviors relevant to the persistent use of PrEP. All participants should be listened to and treated with **empathy, dignity, and respect.** As part of this philosophy, navigators should work to develop rapport with the participant, displaying active listening skills and expressing empathy throughout their engagements.

For this approach to be successful, participants much feel that that the program and its objectives have their best interests in mind, which also means they must feel respected. It is the role of all study staff to make sure any person with participant-facing activities treats participants with respect and support – staff should be encouraged and empowered to communicate any concerns if these expectations are perceived as not being met.

This is also an opportunity to engage in listening with the participants – ask if they are feeling respected in how they interact with people involved in their PrEP care. Notably, the HPTN 112 *study staff* are not involved in any counseling or provision of PrEP services, so these questions may surround how the participant feels when interacting with nurses or other clinic staff involved in PrEP care but not involved in the study. Identifying and empathizing with any instances in which a participant does not feel supported or respected is an important part of building and maintaining the therapeutic alliance.

One objective of this study is to understand how or if stigma influences the desired behavior of continued PrEP use. If persons have or currently feel stigmatized based on their sexual behaviors or other life events, it is particularly important to increase the sense of trust. If the relationship can quickly move to one of mutual trust between participants and navigators, navigators will be able to transition the focus of interactions more quickly to the stated topics and modules, while also increasing the likelihood that participants provide accurate information.

It is expected that participants may have unique barriers to remaining engaged with HIV prevention services, including barriers to adhering to PrEP as prescribed. These barriers exist at multiple different levels – at the individual level they could improve depression, alcohol or substance use, lack of knowledge or inadequate understanding regarding the mechanism of PrEP for HIV prevention, lack of perceived risk of acquiring HIV, lack of resources, or just not perceiving themselves as needing PrEP as a means of protecting against HIV. **Social barriers** could include lack of social support from family, friends, or sexual partners, fear, misunderstanding by family, friends, or sexual partners, and inability to communicate with health care providers. **Structural barriers** could include things such as long wait times for PrEP care (and the associated lost wages because of PrEP service hours), and time and resources to travel to PrEP clinics.

The HPTN 112 intervention is designed to be **flexible and tailored to meet different needs of participants.** There are no required topics, but there are recommended topics to help orient participants to PrEP use, adherence, and disclosure, all with associated skill building exercises and activities. Other modules can and should be directed based on the observed/elicited barriers as described by participants or, if no barriers are identified, selected by the navigator. For example, even if a participant does not identify alcohol use as a barrier, but does report that they drink alcohol, the navigator my offer this as a module to discuss – it is possible the participant does not recognize alcohol as a barrier to effective PrEP use, even if their alcohol use patterns could interfere with their PrEP care. Remember, if a participant reports the same barrier or an ongoing barrier at different sessions, it may be preferable to revisit that barrier and provide the appropriate support and skill building to address on multiple occasions.

The primary study goal of HPTN 112 is to improve persistent use of PrEP – which includes adherence to medications as prescribed as well as attendance to PrEP clinic visits as required based on PrEP modality – compared to persons who do not receive this navigator intervention. Any factors that interfere with PrEP adherence (to daily, event-driven, or injectable PrEP) should be addressed. Systems Navigators occupy two major roles in this intervention – one focusing on addressing interpersonal **(individual level)** and intrapersonal **(social level)** drivers and barriers**,** and the other focused on helping the participant work within the health care system (structural level) to engage in continuous PrEP services.

It is expected that participants will have a wide range of psychosocial issues, not all of which will impede medication adherence. The focus should be on addressing those issues that are felt to interfere with persistent PrEP use, using a risk-centric approach. **Risk-centric** suggests that navigators should engage with and elicit information from participants regarding their ongoing HIV risk. This is further informed by the **point-of-care** (POC) **sexually transmitted infection** (STI) **testing** that is to be done at all in-person PrEP visits. In doing so, the goal is to identify potential behaviors that may put participants at ongoing risk of HIV, understand their perceived risk of acquiring HIV, and, as appropriate, maintain or motivate PrEP use as aligned with their ongoing HIV risk. Navigators collect urine from the participants during their PrEP visit to perform this POC STI testing and return results as soon as they are ready (either during the navigation session, or soon after by the confidential tracing mechanism selected by the participant).

Each Navigation Session will provide information about health and wellbeing as well as include skills building, problem solving, and goal setting. All subsequent sessions should review any goals from the prior session and discuss participant’s efforts to reach these goals and barriers encountered, or what helped them. It is important to encourage participants to set goals but it may not be feasible for a participant to set a goal at each session. Suggesting problem-solving methods to overcome barriers when reviewing goals can be an effective strategy.

There are several different types of goals – some may be shorter term (i.e., use a condom when having sex while traveling next week), and others may be longer term (i.e., stop using alcohol, improve relationships with families or friends, or improve adherence to PrEP medications). With goal setting exercises, major or “longer term” goals can be broken down into smaller, more easily accomplished goals. Attempt to problem solve each smaller goal, thinking about how the participant may help to achieve this goal as a team or partnership. For stopping alcohol use, it may be thinking about the situations that often result in their drinking, the persons, or places, and then setting goals or problem solving regarding how they could avoid these high-risk situations that often trigger alcohol consumption for the participant.

If a goal is set, the navigator should follow-up by asking about what the potential barriers to achieving that goal is and how the participant wants to address them. Role playing with the participant for difficult conversations (such as disclosing PrEP use or inquiring about a partner’s HIV status and testing history/ART use) can help solidify some of the skills from a given session. It may also be helpful if the navigator teaches the participants about how to reframe “failure” to achieve stated goals. Navigators can provide statements that encourage, such as: “it is great that you set goals, even if you don’t achieve each goal! The act of setting a goal lets you learn a lot about what kind of things you can do and even working towards those goals is something to be proud of.” Throughout, it is important that the navigator offers encouragement and acknowledges the success of the participants.

With permission from the participant, navigators are also responsible for reminding participants about upcoming PrEP visits, using the preferred method of the participant, and based upon the frequency and timeline perceived to be most helpful by the participant. Navigators will also trace participants for missed PrEP visits and encourage them to return to the clinic for their missed visit. Navigators will complete this tracing in a way that maintains the confidentiality of participants and does not jeopardize their privacy. Participants are asked to indicate how they would like to be traced, including what name the navigator should ask for, places that are ok to be traced in person if needed, and how the navigator should identify themself. If a participant decides to stop their PrEP use, the navigator will also offer the participant a PrEP “re-start kit” so that the participant may reinitiate as/if desired. The re-start kit includes: a supply of oral PrEP, an HIV-self test, and brief instructions to facilitate safe immediate restart if risk recurs without their first re-engaging in clinic-based PrEP care.

**CHAPTER 3: COUNSELING TECHNIQUES**

Behavior change can be achieved through a psychotherapeutic approach and then skills building, problem solving, and goal setting. This approach to helping facilitate behavior changes utilizes numerous counseling techniques. All these techniques are tools that systems navigators can use during encounters.  It is not required that systems navigators are highly trained in these tools. Each technique may or may not be useful for addressing barriers faced by participants, it is up to the system navigator to determine if these techniques are beneficial.  It is important to remember that the overall goal for this intervention is to improve persistent PrEP use to interrupt HIV acquisition.

Navigators should learn all the techniques and **ensure that the sessions are interactive and not didactic.** Some approaches that can make sessions more engaging as outlined in this chapter include:

1. Active listening
2. Motivational interviewing
3. Role-playing
4. Cognitive Behavior Therapy

### **Active listening**

Active listening is a critical approach to build rapport with participants, in addition to developing and maintaining understanding and trust.  Active listening helps participants feel that they are being heard that their opinions are being seen, and that their feelings are being understood, thereby helping to facilitate the sense that the navigator and the participants are “in it together.” This strategy allows for the navigator to establish acceptance and impartial reflection of the participant’s experience. **It is important that the counselor does not provide their own opinion** but attempts to understand the participant.

There are 4 main techniques of active listening:

- 1. Paraphrase
  2. Summarize
  3. Clarify
  4. Reflect

1. **Paraphrase**:

This involves restating the information provided by the participant using different words. This technique allows the participant to focus on the content of what they are saying, to hear it said back to them. And, if appropriate, to correct or modify. Paraphrasing must be done without making judgments about the participant’s discussion.

Tips for using paraphrasing:

- Use phrases such as “I’m hearing you saying…” or “It sounds like you are saying…”
- Repeat key words but do not repeat the exact statement
- Avoid phrases like “I know what you mean.” You can build this rapport and trust without this form of agreement

Sample dialogue:

*Participant: I* am feeling tired these days and I feel like my PrEP medications are making me even more tired.

*Navigator:* It sounds like you are having problems with your PrEP medications.

1. **Summarize**

To concisely reiterate several of the highlights or key points from the participant’s discussion.  This technique allows the navigator to review overall progress or barriers and recognize any common themes or overtones that may occur during discussions.

Tips for summarizing:

- Pull together major ideas, facts, or feelings
- Avoid phrases such as, “Do you have any questions?” or “Do you understand?”
- Avoid adding new ideas during summarization

1. **Clarify**:

To ask the participant to explain or possibly re-state an element of the discussion that was vague or not clear to the navigator.  This technique allows the participant to expand on their thoughts or feelings and allows the navigator to check the accuracy of the participant’s statements and their own understanding.

Tips for clarifying:

- Do more than just asking “why” as this may sound threatening
- Use open, neutral questions that further draw out the participant’s opinion
- Use phrases such as, “Can you tell me a little more about…?” or “Can you help me understand why you feel that way?”

1. **Reflect**

To rephrase the participant’s emotions or feelings. This technique is “a way of checking rather than assuming that you *know* what is meant”.

Much of what we say is said without words – so-called “nonverbal communication.” Nonverbal communication plays an important role in active listening.  Facial expressions, appropriate eye contact, posture, gestures, and movements are all examples of nonverbal communication.  A navigator can maintain steady eye contact (without staring!), look attentive, lean forward, and nod their head as ways to nonverbally communicate to a participant that they are listening to what is being discussed and are engaged and interested in the feelings and thoughts of the participant.

Just as navigators should be aware of nonverbal communication, participants may also express themselves using nonverbal communication. These nonverbal communications are important cues for a navigator to pay attention to, clueing them into places where the words a participant may say could disagree with how they feel.  Below are examples of some nonverbal communications and their *possible* meanings:

| Nonverbal cues | Possible meaning |
| --- | --- |
| Wavering eye contact | Boredom or fatigue |
| Intense eye contact | Fear, confrontation, or anger |
| Rocking | Fear or nervousness |
| Elevated voice | Discomfort or nervousness |
| Prolonged and frequent periods of silence | Disinterest, loss of train of thought, or fatigue |
| Fidgeting | Discomfort, disinterest, or nervousness |

### **Motivational interviewing**

Motivation is one approach for addressing PrEP adherence.  Motivational interviewing is a therapeutic style intended to help navigators work with participants to address their ambivalence about initiating and adhering to PrEP and the associated part of PrEP care (visits, testing, etc).  Participants may be ambivalent to initiating PrEP or eager to begin. Some participants may be ambivalent about PrEP adherence, while others may want to adhere closely to PrEP guidelines, but face challenges in doing so.  As part of the HPTN 112 intervention, we will use **motivational interviewing** tools to address ambivalence.  Navigators will need to understand and accept ambivalence because ambivalence can often be the main problem—and lack of motivation can be part of this ambivalence. Remember that ambivalence to initiating or adhering to PrEP may not always be stated verbally by the participant, it may also be apparent in their actions. If they continuously agree to a plan of action but do not act on it, motivational interviewing may also be useful to address the underlying ambivalence to making changes to one’s life or behaviors.

Motivational interviewing is practiced with 5 main principals:

1. Express empathy through reflective listening.
2. Develop discrepancy between participants’ goals or values and their current behavior.
3. Avoid argument and direct confrontation.
4. Adjust to participant resistance rather than opposing it directly, also known as “rolling with resistance”.
5. Support self-efficacy and optimism.

**Principal 1: Express empathy through reflective listening.**

Expressing empathy towards a participant shows acceptance and increases the chance of the navigator and participant developing a rapport.

- Acceptance enhances self-esteem and helps to facilitate change.
- Skillful reflective listening is fundamental.
- Participant ambivalence is normal and expected.

What is reflective listening?

Reflective listening is the foundation of expressing empathy.  This approach establishes a safe and open space that is beneficial for exploring issues and stimulating personal reasons and methods for change. It is important for navigators to understand each participant’s unique perspectives, feelings, and values, which includes getting to know the participant. The success of motivational interviewing relies on the development of a trusting relationship between a navigator and participant.

Reflective listening is NOT:

- *Ordering or directing*. Direction is given with a voice of authority. The speaker may be in a position of power (e.g., parent, employer) or the words may simply be phrased and spoken in an authoritarian manner. This is particularly tempting in healthcare and related interactions, such as those between a prescriber and a patient. *Take care to establish the relationship of navigator as not one in which there is a power differential*.
- *Warning or threatening.* These messages are similar to ordering, but they carry an overt or covert threat of impending negative consequences if the advice or direction is not followed. The threat may be one the clinician will carry out or simply a prediction of a negative outcome if the participant doesn’t comply—for example, “If you don’t listen to me, you’ll be sorry.”
- *Giving advice, making suggestions, or providing solutions prematurely or unsolicited.* The message recommends a course of action based on the clinician’s knowledge and personal experience. These recommendations often begin with phrases such as, “What I would do is….” Make sure to take cues from the participant.
- *Persuading with arguing, logic, or lecturing.* The underlying assumption of these messages is that the participant has not reasoned through the problem adequately and needs help to do so. This interferes with the development and maintenance of the therapeutic alliance.
- *Moralizing, preaching, or telling the participant their duty.* These statements contain such words as “should” or “ought” to convey moral instructions. This suggests a judgement (discussed below) that again interferes with the objective of the intervention.
- *Judging, criticizing, disagreeing, or blaming.* These messages imply that something is wrong with the participant or with what the participant has said. Even simple disagreement may be interpreted as critical, whether it is perceived by the navigator to be related to PrEP use.
- *Agreeing, approving, or praising.* Surprisingly, praise or approval also can be an obstacle if the message sanctions or implies agreement with whatever the participant has said. Unsolicited approval can interrupt the communication process and can imply an uneven relationship between the speaker and the listener. Reflective listening does not require agreement. This is also an important consideration in nonverbal communication – excessive nodding or other cues that may be perceived as direct approval.
- *Shaming, ridiculing, labeling, or name calling.* These messages express overt disapproval and intent to correct a specific behavior or attitude. To correct is to imply there is a wrong that needs to be undone and is contrary to the objectives of motivational interviewing.
- *Interpreting or analyzing.* Clinicians (or navigators, in this case) may be frequently or easily tempted to impose their own interpretations on a participant’s statement and to find some hidden, analytical meaning. Interpretive statements might imply that the clinician knows what the participant’s problem is. The goal is to have the participant state and verbalize the problem with gentle probing and assistance.
- *Reassuring, sympathizing, or consoling.* Clinicians often want to make the participant feel better by offering consolation. Such reassurance can interrupt the flow of communication and interfere with careful listening.
- *Questioning or probing.* Clinicians often mistake questioning for good listening. Although the clinician may ask questions to learn more about the participant, the underlying message is that the clinician might find the right answer to all the participant’s problems if enough questions are asked. In fact, intensive questioning can interfere with the spontaneous flow of communication and divert it in directions of interest to the clinician rather than the participant. This does not mean to avoid ALL questions, just that questions should be asked to help probe or uncover with restraint.
- *Withdrawing, distracting, humoring, or changing the subject.* Although humor may represent an attempt to take the participant’s mind off emotional subjects or threatening problems, it also can be a distraction that diverts communication and implies that the participant’s statements are unimportant. Again, this does not mean humor cannot be an effective strategy for rapport and relationship building but needs to be used cautiously and in a manner that does not distract or override the objective of a participant. It is not always necessary to “lighten the mood.”

What does reflective listening look like?

In this strategy, the navigator listens carefully to what a participant is saying, then reflects it back to the participant in an often slightly modified or reframed form.  The counselor also acknowledges the participant’s expressed or implicit feeling state.

This strategy offers several advantages:

1. It is unlikely to prompt participant resistance.
2. It encourages the participant to keep talking and exploring the topic.
3. It communicates respect and caring, while building a working therapeutic alliance.
4. It clarifies for the navigator exactly what the participant means.
5. It can be used to reinforce ideas expressed by the participant.

Below is a sample dialogue of a navigator (N) and a participant (P), where the counselor uses the reflective listening strategy:

- Navigator: What else concerns you about adhering to PrEP?
- Participant: Well, I’m not sure I’m concerned about it, but I do wonder sometimes if I might forget.
- Navigator: You think you might forget . . .
- Participant: Yes, sometimes I have too much to do in the morning and I can leave without taking my pill.
- Navigator: You have too much to do in the morning and can forget.
- Participant: Yes, and sometimes I have trouble remembering things, so I forget to take it when I get back home.
- Navigator: And you sometimes forget to take it when you get home.
- Participant: Yes, I know I should remember. But it is difficult.
- Navigator: I can see why that would be difficult. Are there other reasons you think you might forget to take your pill?

**Principal 2: Develop discrepancy between participants’ goals or values and their current behavior.**

Developing discrepancy enables the participant to see that his present situation does not necessarily fit into his values and what he would like in the future. A participant rather than the navigator should present the arguments for change. Change is motivated by a perceived discrepancy between present behavior and important personal goals and values.

Examples of developing discrepancy:

**Example 1**: “Hmm. Help me figure this out. You’ve told me that keeping your daughter at your house and being a good parent are the most important things to you now. How does adhering to your PrEP fit in with that?”

**Example 2**: “You mention that you have a wife/parent(s), and you don’t want these family members to be disappointed in you or worry about you. How does your PrEP adherence fit in with that?”

**Example 3:** “You mentioned that you have children, and you want them to study and have a good future. How do you think your PrEP adherence will affect this?

| *Note: be careful not to increase feelings of stigma and discrimination. The point is not to shame or humiliate but to get the participants to start thinking about how some behaviors may be preventing them from reaching their goals, and therefore motivate positive change. If people already know* ***why*** *their PrEP adherence is a problem this will not be needed.* |
| --- |
| **Example 1**: “Hmm. Help me figure this out. You’ve told me that keeping your daughter at your house and being a good parent are the most important things to you now. How does adhering to your PrEP fit in with that?”  **Example 2**: “You mention that you have a wife/parent(s), and you don’t want these family members to be disappointed in you or worry about you. How does your PrEP adherence fit in with that?”  **Example 3:** “You mentioned that you have children, and you want them to study and have a good future. How do you think your PrEP adherence will affect this?  **Example 4:** “You mentioned that you would like to live a healthy live. How do you think adhering to your PrEP will affect this?” |

**Principal 3: Avoid argument and direct confrontation.**

Arguments with a participant can quickly develop into a power struggle and do not enhance motivation for beneficial change.  The goal is to “walk” with participants, like accompanying them through sessions, not “drag” them along or direct their sessions.

- Arguments are counterproductive.
- Defending breeds defensiveness.
- Resistance is a signal to change strategies.
- Labeling or diagnosis is unnecessary. Avoid telling a participant they are “useless”. Then avoid diagnoses such as “you suffer from depression”.

**Principal 4: Adjust to participant resistance rather than opposing it directly, also known as “rolling with resistance”.**

Rolling with resistance prevents a breakdown in communication between participant and counselor and allows the participant to explore her views.

- Avoid arguing for change.
- Do not directly oppose resistance.
- New perspectives are offered but not imposed.
- The participant is a primary resource in finding answers and solutions.
- Resistance is a signal for the counselor to respond differently.

| Rolling with Resistance Strategies | |
| --- | --- |
| Strategy | Example |
| **Simple reflection**: repeating participant’s statement in a neutral form | **Participant (P)**: I don’t plan to stay adherent to daily, oral PrEP anytime soon.    **Navigator (N)**: You don’t think that daily, oral PrEP would work for you right now. |
| **Amplified reflection:** reflect the participant’s statement in a more extreme form | **P:** I don’t know why my wife is worried about this. I only miss PrEP every once in a while.    **N:** So you think that your wife is worrying unnecessarily. |
| **Double-sided reflection:** recognizing what the participant has said but then also stating contrary things she has said in the past | **P:** I know you want me to take PrEP everyday, but I’m not going to do that!    **N:** You can see that there are some real problems here, but you’re not willing to think about adhering to daily, oral PrEP. |
| **Shifting focus:** defuse resistance by helping the participant shift focus away from obstacles and barriers | **P:** I can’t take PrEP because I don’t want my friends to see it.    **N:** You’re way ahead of me. We’re still exploring your concerns about PrEP. We’re not ready yet to decide what might be the best way for you to take it. |
| **Agreement with a twist:** agree with the participant, but with a slight twist or change of direction that propels the discussion forward | **P:** Why are you so stuck on PrEP? What about all my other problems? I am worried about school fees and how to feed my family.    **N:** You’ve got a good point there, and that’s important. There is a bigger picture here, and maybe I haven’t been paying enough attention to that. |
| **Reframing:** offering a new and positive interpretation of negative  information provided by the participant | **P:** My wife is always nagging me about PrEP because she knows that I have other partners. It really annoys me.    **N:** It sounds like she cares about you and is concerned for you both, although she expresses it in a way that makes you angry. Maybe we can help her support your PrEP use in a way that is more acceptable to you. |
| **Siding with the negative:** to take up the negative voice in the discussion | **P:** Well, I know some people think I need to adhere to PrEP, but I still don’t really think I need it.    **N:** We’ve spent considerable time now going over your positive feelings and concerns about PrEP, but you still don’t think you are ready or want to change your adherence. Maybe changing would be too difficult for you, especially if you really want to stay the same. Anyway, I’m not sure you believe you could change even if you wanted to. |

**Principal 5: Support self-efficacy and optimism.**

Self-efficacy is a crucial component to facilitating change. If a participant believes that he can change, the likelihood of change occurring is greatly increased.  This is known as “self-motivation”. A person's belief in the possibility of change is an important motivator. The participant, not the navigator, is responsible for choosing and carrying out change. The navigator’s own belief in the participant's ability to change becomes a self-fulfilling prophecy.

| Sample questions to elicit self-motivational statements |
| --- |
| **Problem recognition**   - What things make you think that this is a problem? - What difficulties have you had in relation to your PrEP use? - In what ways has this been a problem for you? |
| **Concern**   - What worries you about PrEP? What can you imagine happening to you? - How much does this concern you? - In what ways does this concern you? - What do you think will happen if you don’t make a change? |
| **Intention to change**   - The fact that you’re here indicates that at least part of you thinks it’s time to do something. - What are the reasons you see for making a change? - What makes you think that you may need to make a change? - If things worked out exactly as you would like, what would be different? - I can see that you’re feeling stuck at the moment. What initial steps do you think are needed to make a change? |
| ***Optimism***   - What encourages you that you can change if you want to? - What do you think would work for you, if you needed to change? |

**Core Motivational Interviewing Skills (OARS):**

**Open questions:** to explore concerns, promote collaboration, and understand the client’s perspective.

**Affirmations:** to support strengths, acknowledge effort, and convey respect.

**Reflective listening:** to explore deeper, convey understanding, and gather information.

**Summarize:** to organize discussion, focus the session, and move to other topics.

Use variety in your reflections: *Sounds like…What I’m hearing is…So you’re saying that…You’re feeling like…For you, it’s a matter of…From your point of view…You are…I would imagine you…Must be…Through your eyes…Your belief is that…Your concern is that…It seems to you that…You’re not terribly excited about…You’re not much concerned about…The thing that bothers you is…The important thing as you see it is…*

When working with people in a helping capacity it is easy to fall into certain traps that are less helpful.

**Do:**

- **Be curious and be the learner**
- **Listen for a client’s insights and ideas**
- **Collaborate and partner**
- **Acknowledge the client’s efforts**
- **Be conversational and be yourself**

**Don’t:**

- **Pressure client to make certain choices**
- **Try to “fix” things for the client**
- **Communicate you have all the answers**
- **Use scare tactics or fear as an approach**
- **Be judgmental.**

**Values and goals:**

- Always a good place to start
- Can always come back to them if needed
- Using a menu of options can be helpful
- Doing it early can also build rapport

**Asking permission respects the client’s ability to choose:**

- Always good to encourage and reinforce autonomy
- Counselor is only with client for short time, so client will need to make own choices
- Equals “playing field” between counselor and client by giving some options to client
- Increases trusting relationship between counselor and client

**Use Elicit-Provide-Elicit (Ask-Offer-Ask)**

- ASK: the client what they already know about the topic (do not assume they do not have any perspectives or information on a topic).
- OFFER: ask permission to offer additional information as appropriate (to share information, correct misinformation, share what works for others).
- ASK: ask for their reaction and how the information shared may fit into their thinking/situation.

**Importance (and Confidence) to learn about strengths and challenges**

To help me understand how important this is to you, on a scale from 0 to 10, with 0 being not at all important and 10 being very important, how important is it to you to (take your pill every evening before bed right now)?

0     |    1     |    2    |     3     |     4     |      5      |     6      |    7     |     8     |     9     |    10

*Why are* *you a __ and not a [slightly lower number]? (learn about strengths)*

*Why are* *you a ___ and not a [slightly higher number]? (learn about challenges)*

*What would it take for you to move from a __ to a _ [slightly higher number**] ? (explore change)*

### **Role-playing**

To help the participant become aware of their automatic thoughts and resulting emotions the navigator may role play different situations with the participant, pausing at points to identify what automatic thoughts are occurring.  This exercise can also be useful to allow participants to practice skills they have been building to address identified problems.

**Here are a few tips to help with introducing role playing with a participant:**

- Pick a concrete situation that occurred recently for the participant or a brief scenario that they think might happen to them.
- Ask the participant to provide some background on the target person for the role play.
- Have participants play the target person, so they can convey a clear picture of the style of the person and the counselor can model effective strategies for the interaction. Then reverse the roles for subsequent role-plays. (Note, if the role play is simple and brief, the participant can play themselves. Ex: asking about side effects in the clinic).
- Role-plays should be thoroughly discussed afterward.
- Navigators should praise any effective behaviors shown by participants and offer clear, constructive criticism (example below).

**Examples scenarios:**

- A partner who finds the participant’s pills and confronts them.
- A family member who doesn’t understand the importance of PrEP adherence.
- A clinic staff member who is rude to the participant.
- A family member or friend who doesn’t approve of the participant’s PrEP use.
- Asking the health care provider about side-effects.

**Example for defaulting from PrEP and reinitiating:**

Round 1:

**Participant:** “You have stopped taking these pills! We instructed you to take these every day or you will get HIV. You are careless.”

**Navigator:** “I am beginning to take these pills again. I was having trouble taking them before because I couldn’t remember. Now I have a plan to remember to take them. I am proud of myself for starting again because I know this is important.”

Round 2:

**Navigator:** “You have waited too long to refill these pills, which means you aren’t taking them currently. Didn’t they explain to you, you need to take them every day? Do you not understand? (shakes head)”

**Participant:** “Yes, I know I need to take them every day. I am working on it…”

**Navigator:** "That was good; how did it feel to you? I noticed that you looked me right in the eye and spoke right up; that was great. I also noticed that you said, “I am trying” and then removed your eye contact. Let's do this once again, but this time, try to be specific to the nurse about why you are feeling better about your PrEP use (so that they don’t continue to reprimand you) and continue to hold eye contact the whole time."

### **Cognitive behavior therapy**

Cognitive behavioral therapy (CBT) is a focused approach to help people reach their treatment goals.  CBT strategies are based on the theory that in the development of maladaptive behavior patterns, learning processes play a critical role.  Individuals in CBT learn to identify and correct problematic behaviors by applying a range of different skills that can be used to stop substance abuse and to address a range of other problems that often co-occur with it. A central component of CBT is anticipating likely problems and enhancing participants’ self-control by helping them develop effective coping strategies.

Specific techniques for CBT to promote PrEP use might include:

- exploring the positive and negative consequences of PrEP adherence
- self-monitoring to recognize challenges to adherence early and identify situations that might put one at risk for a missed dose
- developing strategies for coping with challenges and avoiding high-risk situations.

**Problem solving**

Problem-solving techniques generally involve a process through which a person attempts to identify effective means of coping with problems of everyday living. This often involves a set of steps for analyzing a problem, identifying options for coping, evaluating the options, deciding upon a plan, and developing strategies for implementing the plan. Think back to the problems and goal setting we examined earlier!

Problem-solving strategies can be used with a wide range of problems. Problem-solving techniques teach skills that aid the participant in feeling increased control over life issues that previously felt overwhelming or unmanageable. This could be alcohol use, condom use, or even engaging with preventive health services like PrEP. In this way, problem solving can help with practical problem resolution and emotion-focused coping (e.g., increasing control, decreasing stress, and increasing hopefulness).

**Strategies for Effective Problem Solving**

The SOLVED technique guides us through the steps to effectively identify and solve problems that may be present in a participant’s life.

S (Selecting a Problem) … the participant would like to solve.

Ask the participant to think about situations when he feels distress or difficulty problem solving. If planning does not seem to be possible, suggest a different therapeutic technique. The decision to remain with problem solving or move to a different skill is largely dependent on the navigator to direct.

O (Opening Your Mind to All Solutions)

Here, it is important to be as broad as possible. You are encouraged to work with participants to “brainstorm” all possible solutions. Writing may be particularly helpful for some participants.  Even ideas that seem ridiculous at first may eventually generate realistic solutions.

L (Listing the Potential Pros and Cons of Each Potential Solution)

Often, writing options, along with listing pros and cons, can be helpful in considering potential options. Writing allows additional thought, as well as a visual image of options.  Recommend that participants consider solutions in a logical manner, thus reducing the time spent ruminating.  It may also help to identify additional thoughts that might benefit from changes using techniques, such as changing thoughts.

V (Verifying the Best Solution)

Examine the pros and cons of the solutions listed. Participants may wish to “rank order” the solutions based on which solutions are most practical and/or desirable.

E (Enacting the Plan)

Identify the steps needed to carry out the solution selected. Participants may need to break actions down into steps small enough to facilitate achievement of goals. Once you and the participants finish formulating a specific plan, encourage the patient to carry it out.

D (Deciding if the Plan Worked)

Follow-up with the participants to see how well the chosen solution worked. If the solution was effective, give positive reinforcement. If the solution was not effective, return to the first step in the SOLVED technique to specify a new problem or move to “O” or “L” to identify other goals or potential solutions for the same problem. The decision to move back and to which step is largely up to you, who might now have additional information about pros and cons and possible solutions.

**Example 1:** Select a specific problem: Minimizing Effect of Symptoms

1. Talk to your doctor
2. Change or modify medications
3. Engage in healthy life choices, including proper diet and exercise
4. Educate yourself by talking to others and by reading about your illness.
5. Explore alternative treatments.

**Example 2:** Select a specific problem: Minimizing Effect of Symptoms

1. Turn several alarm clocks on to remind you.
2. Put your medication in a place you will notice it at the time you are supposed to take it.
3. Have a friend or family member remind you.
4. Buy a medication dispenser to help you remember whether you have taken the medication.
5. Take it at the same time every day.

***Goal setting***

Goal setting is the process of collaboratively identifying specific therapeutic outcomes for treatment. Goals must be observable, measurable, and achievable and relate to cognitive or behavioral changes relevant to the participants’ PrEP use. Goals are tied to specific skills to be addressed in treatment.  Tips for goal setting include:

- Provide rationale for setting goals.
  - This helps participants understand why you are asking them to identify goals and how they will be involved in the process.
  - Example: “If you can identify what you want to change about your situation, we can then take the steps to help you make these changes.”
- Elicit desired outcomes.
  - This involves the counselor’s assisting the participant in defining goals and specifying reasons for starting or staying on PrEP.
  - Example: “List a few things you would like to get out of our session today. (or perhaps out of taking PrEP more generally?)”
- State goals in a positive light.
  - This clarifies what the participant wants to do instead of highlighting what he doesn’t want to do.
  - Example: “List some things that you want, instead of things that you don’t want.  For example, instead of ‘I don’t want to avoid taking PrEP anymore, you could list ‘I want to be proactive about my preventing HIV.’”
- Weigh advantages and disadvantages of a goal.
  - This aids in understanding the costs and benefits of the participants’ achieving a specified goal.
  - It may be used to motivate an ambivalent participant or identify salient goals for a passive participant or one seeking to please the navigator.
  - Example: “What would be the benefits if you accomplished this goal?   What might be some of the costs to you?”
- Define behaviors related to goal.
  - This tells the patient what actions to perform in relation to the set goals.
  - Break the goal down into smaller steps.  For example, if the participant wants to remember to take their PrEP at the same time every day, what are all the steps necessary to do that, e.g. attending scheduled PrEP visits on time to make sure their pills are refilled, asking friends and family to support the decision (which may include disclosing their PrEP use to these persons) finding transportation, having food to take with pills to avoid nausea, etc.
- Define a level of change.
  - This determines how much a participant should do a particular behavior. To increase the participant’s chance of success, set achievable goals. In other words, it is usually not reasonable to try to do something every day, and setting a goal like this will result in failure if the participant misses just 1 day.  Alternatively, discuss the goal with the participant; and start small. If the participant succeeds, he is more likely to remain actively engaged.
  - Example: “How often do you think it is reasonable to do something pleasant? Once a week?”

***3. Skills building with participant***

One of the main components of CBT is to provide participants an individualized training program that helps them address their PrEP adherence.  These skills can help participants unlearn old habits associated with inconsistent PrEP use (if they have experienced this in the past) and learn or relearn healthier skills and habits. It is likely participants may present a wide range of problems and therefore, skills building in CBT is made to be as broad as possible to address these problems. Initial sessions should include the focus of skills needed for initial PrEP adherence, such as identification of a high-risk situation (ex. planning ahead for travel to bring pills). Then the skills can be broadened to include a range of other problems in which the participant may have a difficult time coping, such as social isolation and unemployment. It is important that the skills taught not only help a participant improve their PrEP adherence, but also to teach skills that can benefit the participant in other aspects of their life to promote health.

# **CHAPTER 4: TRAINING AND COMMUNICATION FOR IMPLEMENTATION**

All systems navigators are expected to participate in study-specific training prior to implementing the intervention. All systems navigators should thoroughly read the manual and practice a wide range of role plays based on different scenarios.

**Suggested roleplay scenarios:**

- Participant has defaulted from PrEP but is reinitiating now.
- Participant is on PrEP but wants family support for adherence.
- Participant has stopped PrEP completely due to side effects.
- Participant is “yessing” or telling navigator what they think the navigator wants to hear.
- Participant is not taking PrEP consistently or taking PrEP but missing visits.

During role playing activities, navigators should practice documenting the session using all appropriate forms and CRFs. In addition to role playing, mock participant folders can be developed to have navigator discuss recommended next sessions and/or modules for the participant.

Modules and specific content are meant to be driven by a combination of participant and navigator direction. Many of the challenges are expected to be common for participants and thus it is important that the systems navigators learn from each other and share their experiences with each other and with the rest of the study team. It is anticipated that weekly debriefs will be held with the systems navigators and study team to discuss:

- Specific cases/experiences with participants.
- Materials used by counselors.
- Successful counseling tools or techniques.
- Challenges encountered in implementing the intervention materials.
- New barriers identified.
- Novel and successful approaches attempted to address barriers.

These discussions should be guided by the navigator notes/reflections, documented following participant encounters, and a summary of these debriefs documented in the Intervention Debrief Form and sent to the intervention committee.

# **CHAPTER 5: OVERVIEW OF THE BARRIERS TO PrEP ADHERENCE**

Some of the men who are offered and choose to initiate PrEP will be able to quickly engage in and adhere to dosing as prescribed. Some may have even taken PrEP in the past and are re-starting PrEP, others may have just heard about PrEP for the first time the day they are enrolled in the study. Some may have taken medications before, others may not. Some may have also been prescribed treatment for STIs at the same time they were prescribed PrEP, others may not. Understanding these unique contexts even at this first visit is important not only to build rapport but also to establish some expectations.

Adhering to any medication can be difficult, particularly one that people are asked to take when they feel *well*, as is often the case with PrEP. Adherence is made even more difficult when there are other competing life challenges. The scope of barriers to PrEP initiation and adherence are complex and numerous – we present some of these known barriers below, but this is not an exhaustive list.

The barriers have been organized into three main categories: Structural, Social, and Individual (see table below):

- **Systems/Structural Barriers** are limitations of the resources (physical or personnel) or systems (e.g., hours of clinic operation) that make it hard for a person to adhere to PrEP.
- **Social Barriers** are aspects of an individual’s life such as their family dynamics and their relationships with their friends and sexual partner that may make an individual feel less supported to adhere to PrEP.
- **Individual Barriers** are related to the individuals own mental and physical health, perceptions, and knowledge, competing life priorities, substance use, etc. which may make it challenging to adhere to PrEP.

| Systems/Structural Barriers | Social Barriers | Individual Barriers |
| --- | --- | --- |
| **Health systems**   - Medication or testing stock-outs - Preferred PrEP modality (i.e. injectable) not available - Clinic only open during day (i.e. no evening or weekend hours) - Excessive wait-time for PrEP nurse   **Infrastructure/ Systems**   - Transport/travel to clinic is difficult or expensive - Travelling for work or holidays - No time to go to clinic - Been in jail or incarcerated | **Family network**   - Insufficient support from family or spouse - Responsibility to take care of family members   **Friends/ Sexual partner network**   - Insufficient support from friends or partners   **Social stigma**   - Don’t want others to know I am taking PrEP - Fear disclosure of PrEP will lead to rejection or violence   **Housing/ Social stability**   - Homeless - Moving houses frequently     **Healthcare Discrimination**   - Anticipated or experienced mal-treatment by health-care workers - Previously shamed for failing to follow “prescribed” prevention | **Mental health**   - Depression - Stigma - Felt too depressed to go to clinic - Suicidal ideation and past suicide attempts   **Scheduling**   - Forgot appointment or pills, was traveling away from home, experienced a change in routine - Missed appointments and embarrassed/ashamed to go back   **Physical Health**   - Too sick to go to clinic. - Other illness   **Avoidance /Motivation**   - I don’t want to have to take it forever - I do not feel sick so shouldn’t need medicine - I do not think I am at risk of acquiring HIV - I do not think it is important for me to avoid acquiring HIV   **Medication related**   - I prefer to take traditional/nonwestern medicine - I do not think PrEP will help me - The pills are too big to carry with me - I don’t always have food or water to take medicine - I don’t understand when I am supposed to take each pill - Side effects such as nausea, headaches, disruptive sleep, GI problems   **Substance use and risk behaviors**   - Alcohol or drug use prevented me from getting to clinic - Alcohol or drug use prevented me from taking pills as prescribed     **Information**   - I don’t know how to get my PrEP refilled - Inadequate understanding of how HIV is transmitted and how PrEP prevents acquisition |

# **CHAPTER 6: STRUCTURE OF NAVIGATION SESSIONS AND MODULES**

### **Overview of sessions and modules**

All navigation sessions are conducted by the Systems Navigator. Except for the very first visit (the enrollment visit) and the last visit, each session will follow a similar structure. Recall that the HPTN 112 systems navigation intervention is an **extremely flexible and highly personalized intervention**. The objective is to engage participants any time they are coming in for a PrEP visit while they are enrolled in the study – which could last up to 12 months for some persons enrolled early in the study enrollment periods. In general, sessions may take between 15-45 minutes – though it will depend on the content and needs of a given participant. Although there are no required sessions, in general, each session should follow the same general structure. The structure involves: introduction of the module, exercises relevant to the content of the module, goal setting and homework for the next session, a final recap of what was discussed and confirmation of the next scheduled PrEP visit.

There are 10 modules for navigators to choose from when beginning a navigation session. Deciding which modules to conduct can be a process that involves the participant (via discussing the module options to them) or the navigator can suggest a particular module based upon their interactions with the participant. Ultimately, the module chosen depends on the assessment of the participant’s needs and priorities. It is very helpful for navigators to take note of which module they anticipate being most useful for the participant at the next visit in their session notes, so that the navigator can quickly select the appropriate module when the participant presents for their next navigation session. Modules can be revisited to address PrEP adherence when appropriate for participant.

As a reminder, module activities are meant to be suggestions. It is up to the navigator to decide which activities may be most helpful for any given participant, when to conduct them in the session, and in which order. The scripts provided are also meant to offer a guide to the navigator, but the navigator should feel comfortable enough to discuss the content with the participant in a way that feels natural and does not make it seem as if the navigator is reading directly from the paper provided.

Documentation of which module(s) were conducted and how they were received by the participant are crucial for both the success of the individual participant as well as the navigator team. If navigators find that a particular activity is not resonating with multiple participants, it is helpful to document this in the navigator’s journal in order to discuss the finding during the weekly de-brief meeting.

**Introduction module:** This introduction will help the navigator get to know the participant and assess their needs and priorities for HIV prevention. At the first encounter, the navigators should take this as a time to build rapport, understand any prior PrEP use, and start to explore some of the participant’s unique context that may influence their ability or motivation to remain engaged in PrEP care. Recall that most persons will have also just been identified as having symptoms of an STI and treated for said infection (or sent home with additional treatment for management of that syndrome), so assessing understanding of the various syndromes and medications may be important at this early visit.

The introduction (for non-enrollment visit sessions) can also be a time for the counselor to review the homework from a previous encounter. The introduction will be followed by information and/or activities related to the objectives for the encounter.

**Final module:** The final module is meant to help provide some closure and next steps/guidance for participants as they proceed on their PrEP journey, without your presence as a navigator. You can use this to summarize some of the skills gained and accomplishments achieved.

**Navigation session documentation**

The navigator is responsible for completing several key documents during and after navigation sessions. A detailed overview of the documentation and the timing with which they should be completed can be found in Chapter 9. The key documents for a navigator to have with him during the session are:

- Session checklist (to be completed during the navigation session)
- Session notes (to be completed during the navigation session)
- Navigator Contact Case Report Form (to be completed after the navigation session with information contained within the session notes).

# **CHAPTER 7: RESPONSIBILITIES OF THE SYSTEMS NAVIGATOR**

The primary responsibility of the systems navigator is to identify and help address barriers for adhering to PrEP. Examples of barriers may include:

- Lack of perceived need for/importance of PrEP.
- Challenges getting to the clinic to refill their PrEP.
- Side effects from the medication.
- Stigma, threat of violence, or experienced violence from their family, friends, sex partners, or themselves, that prevents them from taking PrEP freely.
- Travel for work or pleasure that interferes with PrEP dosage (oral PrEP), refills, or receipt of on-time injections.
- Forgetting to take their pills every day or in accordance with event-driven PrEP adherence guidelines.
- Trouble with family, friends, colleagues, or partners that impacts their mood such that it interferes with their ability to adhere to or refill their PrEP.
- Other mental health challenges (i.e. depression) interfering with mood.
- Lack of confidence that they can adhere to their PrEP as prescribed.

The systems navigators also need to have detailed knowledge of PrEP care, related clinical services, and tools to address or at least attempt to minimize or workaround system-level barriers to consistent engagement in PrEP care. Systems navigators can utilize psychosocial counseling tools, such as goal setting, CBT, and MI, but do not need a high level of training in these tools. Rather, these tools can be used as a theoretical underpinning or guide to help facilitate encounters with a participant.  For example, the system navigator could help a participant plan to ensure they can attend their next PrEP visit and then review the necessary steps to get to the appointment and identify challenges they may encounter (making sure not to schedule work, planning for transport, etc.).

 There are several “tasks” systems navigators are responsible for completing:

| Task | Details | Notes |
| --- | --- | --- |
| **Task 1:** Prepare for your week | Each week, you should look ahead on the calendar to identify the expected PrEP visits that may occur, and provide necessary reminders per participant-stated preference | Make sure to keep clear documentation of participant needs and timeline for visits (and reminders) on the central calendar, including indicating when a task is complete. |
| **Task 2:** Meet participant & prepare for navigation session | After you are notified by clinic/ 112 study staff that a participant has arrived, review the participant folder, collect the participant, and determine visit flow based upon clinic line. | If the PrEP visit occurs on the same day as a study visit, you may have time to review the participant folder while the 112 nurse completes the study visit. |
| **Task 3:** Specimen collection | Collect a urine sample from the participant for STI testing. | It is ideal to complete this task as soon as you are aware that a participant has arrived for PrEP care. It takes 45-60 minutes to run this test so earlier collection and transport to lab will make it more likely that you can return the STI test result the same day. |
| **Task 4:** Confirm participant information | At the beginning of the visit, confirm participant locator information & PrEP modality are correct. | Use the enrolment CRF to confirm. |
| **Task 5:** Select and conduct modules | Based on participant folder review and stated preference or interest with the participant, together choose 1-2 modules from the list for review and discussion. | You can determine the number of modules to conduct based upon participant desire, time constraints and module length. |
| **Task 6:** Wrap-up navigation session | Return STI results to the participants, recap session & remind them of their next PrEP visit. *Trace participant for STI results if they have left the clinic.* | Refer for STI care, if necessary, confirm next appointment details are correct. |
| **Task 7:** Complete visit paperwork | After the participant has left the clinic, complete all required CRFs and visit diary. | Complete any additional tasks if required (i.e. social harm, referral, etc.) |

**Description of the role of systems navigator:**

**Systems navigators are to discuss and address barriers to PrEP use with participants**. This should be contextualized by ongoing HIV risk – both the perceived risk as described by the participant, and other markers/indicators of risk as elicited during completion of the risk screener and the STI test results.

Required knowledge:

Familiarity with basic PrEP modalities and relevance of on-time dosing for effectiveness, barriers to PrEP use, knowledge of possible solutions, and counseling techniques to help participant identify possible solutions and take steps toward achieving them. Systems navigators are not intended to replace medical care or counseling/knowledge that would be expected of a medical professional, including a PrEP provider.

Examples:

- Some of the barriers the system navigator may need to address by talking to health care providers or other clinic staff (systems/structural barriers, see chapter 5).
- Some of the barriers may be due to individual factors such as money for transport or social factors such as relationships with family (see chapter 5).
- If the systems navigator identifies systems/structural barriers that they will address, such as talking to a health care provider about an issue, they should first inform the participant of their intended action and confirm the participant has no objection, and then contact the participants later to inform them about the outcome.

**Systems navigators are to address communication issues between health care staff and participants.**

Required knowledge:

Systems navigators will need to have an in-depth understanding of PrEP care services and how to address any barriers within the bounds of the existing scope of services available.

Examples:

- A participant may inform a navigator of a negative interaction that they had with a healthcare provide or staff member, and this may impact their willingness to remain engaged in PrEP care. The systems navigator can work with the participant to identify solutions and communication with this provider to help mitigate this impact.

**The systems navigators may meet with PrEP nurses to answer questions about the study.**

Required knowledge: Systems navigators should learn enough about PrEP to be a source of knowledge. They should also have sufficient information to help participants avoid typical problems and barriers to PrEP (i.e., be familiar with typical side effects and how long it is expected for an individual to experience these effects).  It can be useful for the systems navigators to learn from the PrEP providers about their clinic and the typical issues that the health care providers address, and what a typical PrEP visit may entail.  The more the systems navigators know about the health care system the more they can be proactive and assist participants as well as enhance relationships with providers.

**Systems navigators are to reinforce counseling.**

The systems navigators need to know all of the counseling techniques covered in the intervention. They will use these techniques to help set goals with the participants and help identify barriers and methods to achieve these goals. The system navigators should provide positive feedback to participants in order to increase their self-efficacy and help them set realistic goals and identify barrier to those goals.

**System navigation session structure:**

Goal: the goal of navigation sessions is to assess the participants’ PrEP use, perceived and more objective indicators of HIV risk, and identify any barriers to PrEP persistence – including attendance to scheduled visits and adherence to medications as prescribed.

Prior to the session:

Prior to the session the system navigator should review the participants chart (containing the counseling summary forms and counseling diary, see chapter 11) to determine what goals have been made and then ask about achieving the goals. For example, if the participant had a goal of disclosing their PrEP use to their partner, then the system navigator will ask if they were able to do so.  If they were unable to disclose, then the navigator will ask about barriers that were encountered. This may serve as the motivator for the selection of modules to address, if disclosure to partners remains a priority for the participant.

During the session:

| Enrolment visit | Subsequent visits |
| --- | --- |
| - Enrolment introduction module. - Set goal for the session (related to PrEP goal). - Confirm locator information and preference. - Assign homework. | - Subsequent introduction module. - Review previous session’s homework. - Discuss any interim contact that may be relevant to PrEP use - Set goal for the session (related to either the current goal or a new goal). - Review and update locator information and preference. - Assign homework for the next session. |

After the session:

Complete the Systems Navigator Contact CRF, document the session notes and make a note of which modules are likely to be most useful for the participant at their next navigation session. Remember, in the event that one navigator is out, another will need to step in in his place, so thorough and detailed documentation can help facilitate this handoff.

**Tracing visits for missed PrEP follow-up visit or return of STI test results:**

If needed the systems navigator may conduct tracing visits via phone and/or in-person at the site and tracing modality specified by the participant (ex. home or work). The preference for text, phone call, or in-person should be established and confirmed at each visit. Details regarding when they can or cannot call, if there are contacts or other persons they are able to make inquiry with in the event of an in-person tracing, and other details should all be documented in the updated locator form. It is important to remind the participant that the navigators’ role is only to trace in the event of missed PrEP visits or for the return of STI test results– there are other procedures in place for study visit adherence to HPTN 112.

Any communication by text must be entirely confidential – that is, no sensitive information about the name, the nature of the contact/reason for contact, or personal health information (i.e. STI test result) should be made by text. It is important that the navigator clarify this expectation with the participants ahead of time to avoid confusion.

If the conversation is by phone, the navigator will first assess whether it is an appropriate time for the participant to discuss their health care needs. The navigator should ask the participants if it is a good time to talk or whether it would better to talk another time. If the participant indicates that it is not a good time to talk then arrange for another call. If the system navigator is unsure, then ask about the participant’s setting and potential for interference by others.

With the exception of the enrollment visit, in addition to provision of systems navigation, the navigators are responsible for conducting point-of-care STI testing and providing the results to participants. Ideally, results will be returned to participants before the navigation session has ended. However, if participants choose to leave before their results have been returned, the navigator is required to attempt follow up with the participant and deliver their test results.

If a participant is traced for a missed PrEP follow-up visit and decides that they would like to discontinue PrEP (and/or the navigator learns of this through interaction with the participant at the clinic) the navigators will offer the participant a PrEP “re-start kit”, explain the contents of the kit as well as intended use of the contents. The re-start kits include an HIV self-test and oral PrEP tablets so that the participant can reinitiate PrEP without needing to come back to the clinic.

Forms to be completed:

Systems Navigator Contact CRF

**Other interim contacts:**

The systems navigator is encouraged to use text messaging if it is deemed useful and helpful by the participants. Messaging can be used to remind participants about appointments. Specific messages should be developed that do not lead to disclosure regarding their use of PrEP, the identity of the navigator, or the nature of the connection. Discuss the content of these texts in advance with each participant.

# **CHAPTER 8: MODULES**

| Module | Suggested or optional |
| --- | --- |
| A. Introduction to study and preliminary needs assessment | Suggested module (enrollment visit) |
| B. PrEP Overview and Adherence | Optional module |
| C. Barriers to PrEP Adherence and Problem Solving | Optional module |
| D. PrEP Disclosure | Optional module |
| E. Sexual Health | Optional module |
| F. Circumcision | Optional module |
| G. Substance use (non-alcohol) | Optional module |
| H. Alcohol use | Optional module |
| I. Psychosocial distress | Optional module |
| J.  Wrap-up | Suggested module (participant’s last visit) |

### **MODULE A: INTRODUCTION TO NJIRA AND GETTING TO KNOW THE PARTICIPANT**

***[NOTES FOR SYSTEMS NAVIGATOR]***

*[It is important to spend time building rapport with the participant at this first visit. We want participants to enjoy sessions, so they are motivated to come back.]*

*[It will be helpful to take good notes of what the participant tells you in the participant’s session notes so that you can remember what they said in the next session. This will help with continuity and give the participant confidence that you are listening and that you care.*

*Take note in this module as well of which modules you think might be most helpful for the participant. For example, if they say they take alcohol often and worry that can get in the way of their PrEP, you might make a note that the “alcohol” module could be helpful.]*

*[Assess the amount of time the participant has already spent that day for the enrollment activities. If it was a long time, and the participant appears tired, keep the introductory section short. As with all modules, you can always come back to any part of this module if you think it could be helpful at a future navigation session.]*

*[The goals and objectives for the Introduction module are]:*

- *Introductions and rapport building between the navigator and the participant.*
- *Explain the purpose of the navigation sessions.*
- *Get baseline information on needs of the participant.*
- *Identify possible PrEP barriers and prioritize modules accordingly that might be helpful for the participant.*
- *Set PrEP or health/STI risk reduction goal.*

*[As a reminder, here is a list of all of the other PrEP modules to refer to (when taking notes in the session notes document).]*

| **Module title** |
| --- |
| PrEP overview and adherence |
| PrEP barriers to adherence and problem solving |
| PrEP disclosure |
| Sexual Health |
| Circumcision |
| Substance use |
| Alcohol |
| Psychosocial |

**PART 1: INTRODUCTION**

Welcome to our first counseling session for the NJIRA Study (HPTN 112). My name is [*counselor*] and I will be your systems navigator. My role in is to work with you towards the goal of adhering to PrEP and any other goals you might have to improve your sexual and overall health. I am part of the PrEP team, and will meet with you when you come to the clinic to refill your PrEP.

The NJIRA study is designed to assist men to feel confident about using PrEP. There will be a counseling session at each of your PrEP visits. The number of sessions we want you to come to is dependent on which PrEP method you choose and could be either every two or three months. The sessions are expected to take anywhere from 10 minutes, depending on how you would like me to help you and what you would like to talk about.

I will spend some time today first getting to know you and your goals related to PrEP, and a bit about what motivated you to start PrEP. We will be developing skills that may be helpful for you in sticking to taking your PrEP and other things that are important for you in your life. We appreciate that people have different priorities and goals in their lives and want to ensure that this activity is useful to you.  22

**PART 2: EXPECTATIONS AND ORIENTATION**

[ACTIVITY A: DISCUSS AND AGREE TO EXPECTATIONS]

First, I want to review some important guidelines with you about our approach to these sessions.

**What should you expect from me?**

- Confidentiality
- Collaboration with you during our sessions
- Focus on your health
- Respect for your ideas and opinions
- Open to feedback and willingness to adapt to your needs

**What should you not expect from me:**

- Your PrEP pills or injection: though I am here to help you with navigating your PrEP appointments, adhering to PrEP and working with you to improve your health overall, I cannot provide you your PrEP directly.
- Other medical care: I am not a healthcare provider, so I cannot give you medical advice or treat you for any medical needs. I can however, work with you to make any referrals you might need to healthcare providers at Bwaila.
- Compensation: as reminder, I am part of your PrEP visits, not your study visits, so I will not be compensating you for our time together. You will receive compensation for meeting with a study nurse, but not for your time with me or your time at the PrEP clinic. It is possible that your PrEP refill appointment will align with your study visit, but they are indeed separate.

**What do I expect from you?**

- Meet with me whenever you are at the clinic for a PrEP visit (we can keep it short if needed!)
- Be open and honest
- Willing to give feedback and tell me how I can help you achieve your goals

*[Ask the participant]*

Are we together on these expectations? Are there any other expectations that you would like me to set?

[ACTIVITY B: WHAT IS A NAVIGATOR]

My role here is to work with you over the course of the next few months to understand why PrEP is important to you, and what we can do together to help you achieve your health goals, including staying on PrEP. I am not a medical provider and will not offer medical advice. I am here to help you navigate both the clinic systems, and coach you through some of the challenges that many face when they start PrEP. I can help remind you about appointments, can help follow-up if you miss appointments, and hopefully work with you to develop tricks or strategies to help you stay on your PrEP and keep you protected from HIV.

*[Ask the participant]*

Do you have any questions about my role here in the clinic or with you?

[ACTIVITY C: GET TO KNOW THE PARTICIPANT AND THEIR PRIORITIES]

Now that we have talked a bit about the navigation, I would like to learn a little more about you, so that I may get to know you a bit, and be able to know how to best support you.

*[Use these questions as prompts]*

- Who you live with?
- What sorts of things you like to do for fun?
- What you do for work? Do you travel for work?
- What are the things most important to you?
  - Why are these things important to you?
- Is it important to you to stay healthy?
  - Why or why not?

*[thank the participant for sharing with you when they are finished]*

[ACTIVITY D: PREP KNOWLEDGE AND POTENTIAL BARRIERS]

Now I would like you I know you have just provided some of this information to the study team at your visit that may seem similar to the following questions I am going to ask you now. This is because we keep the information you provided to the study nurse confidential, so that I do not see the answers you provided her. I will not ask you as many questions as the study nurse, but just a few to better understand some things about you so that I can tailor my support to your needs. Please give me the most accurate information you can. I am going to take some notes so that I can assist you. The notes will be confidential.

**Discuss motivations for PrEP use, PrEP knowledge, and potential barriers to adhering to PrEP**

*[In this section, explore their knowledge about PrEP. Start to engage in what is motivating them to take PrEP, and what they foresee as barriers to PrEP. Note that this is likely a topic that will be revisited at future sessions, so don’t feel that you need to explore or explain all topics at once. The purpose of the below probes is to help you identify areas to explore in future sessions.]*

*[Especially when persons are initiating PrEP after being diagnosed for an STI, it is important that they understand the difference between their PrEP and their STI treatment. Ask some questions about their understanding of PrEP specifically as it differs from their STI treatment.]*

*[Questions you might ask the participant to help with understanding of PrEP]*

- I know you may have been treated for an STI today, and possibly sent out with additional medications to take for that treatment. In what ways is your PrEP different from the STI treatment? [offer explanation or clarification]
- Do you anticipate wanting to continue to take PrEP even after your STI treatment is complete? Why or why not?

*[Focusing on why PrEP is important to the person is a good lead-in to why it may be difficult for them to continue PrEP. Remember some men will have been started on injections, and thus the main focus is going to be on remembering their next visit, whereas others will be needing to remember a pill either every day or around the times they have sex.]*

*[Questions you might ask about why PrEP is important to them:]*

- Why is PrEP important to you?
- Tell me about any concerns you have about taking PrEP?
- What are some of the things that may make it difficult to take your PrEP as it was prescribed?
- What are some of the things that you think could make it easier?
- What do you think about telling other people that you’re starting PrEP?

*[Questions you might ask about any history or familiarity with PrEP:]*

- Have you ever taken PrEP before?
- Do you know anyone who has taken or is currently taking PrEP?

*[Take note on which modules may be appropriate to consider at your next visit and continue to revisit these concepts of PrEP. You may also want to start to explore other means/modes of preventing HIV, as these can be discussions or modules to explore in the future].*

*[Questions you might ask about HIV prevention behaviors]:*

- Are you circumcised? If not, what have you heard about circumcision?
- When you have sex, what are some of the reasons you find it difficult to use a condom?
- What are some things that make it difficult to talk to your partners about their HIV status? What are some things that have worked for you when trying to have this conversation in the past?

*[Oftentimes, substance use can interfere with achieving healthy goals, including PrEP use. Explore how or if substance use may be something that the participant wants to explore further in the future to improve their persistent PrEP use.]*

*[Questions you might ask about a participant’s substance use]:*

- How often do you use alcohol or other drugs? Are there certain times of the day or days of the week that you are more likely to use these things?
- Do you often have sex on the days that you also drink or use other drugs?
- Have you noted any difference when you have sex after drinking (i.e. more difficult to remember to use a condom, more likely to have sex with people you don’t know well, etc.)?

[ACTIVITY C: REVIEW PRIORITIZED MODULES WITH PARTICIPANT AND ASK HOW TO PROCEED]

Thank you for sharing all of that information with me. It is very helpful for me to understand how I can best support you during our time together. As I mentioned, I was taking notes while we were talking, and came up with some possible topics for us to discuss during our next sessions. Would you like to hear about those?

*[Review list with participant: ask if they would like any others to be included].*

**PART 3: SET PREP GOAL**

*[As you are setting a goal, try and make sure that you are using some of the counseling techniques to make sure that it is the participant’s goal, not you dictating it.]*

One of the approaches that can be helpful when we are trying to do a new thing, like taking PrEP, is to set goals. I would like you to think of what is important to you, why you decided to take PrEP, and, more broadly, what some of your priorities and/ or goals are for your life at the moment? **What are some things that you would like to accomplish?**

When thinking about this, we would like you to focus on your health, especially staying on PrEP. What is a good goal we can work toward together?

*[Document goal in the session notes]*

**PART 4: SUMMARY OF MODULE**

Thank you for taking time to talk with me today. I know it has been a long visit. As a reminder, we discussed today:

- Our expectations for working together, including how you would like me to contact you in the future for reminders or if you miss a scheduled visit.
- The purpose of our time together.
- I got to learn a little bit about you and how I might best be able to help you.
- We set your PrEP goal for working together.

Thank you again for your time today. Do you have any questions for me?

[*Document: all possibly helpful clinic notes* modules that may be helpful in the participant’s notes]

### **MODULE B: PREP OVERVIEW**

*[NOTES FOR SYSTEMS NAVIGATOR]*

*[Before you begin, remind yourself which type of PrEP the participant is using. This will inform some of the information you provide the participant.]*

*[Feel free to adjust some of the language that is future-facing (for example, asking about PrEP barriers in the future, if they have already discussed barriers to PrEP adherence with you in prior interactions). Try to remind them of things they have already said so that they know you are taking good care to listen to them.]*

*[The goals and objectives for the PrEP Overview module are]:*

- *[Encourage participant to be an active part of discussion around their PrEP use, including dispelling myths and rumors.]*
- *[Conduct activities which help the participant plan for taking their PrEP.]*
- *[Set homework for next session.]*

**PART 1: INTRODUCTION**

We are going to focus on understanding what PrEP is and discussing any rumors you have heard about PrEP. I’m also going to ask you some questions to make sure we are on the same page when discussing PrEP. Don’t worry if you don’t know the answer. That is why we are here today. Remember knowledge helps us become more empowered.

**PART 2: ACTIVITIES**

[ACTIVITY A: PREP 101]

What does PrEP stand for? *[PAUSE]*

PrEP stands for “pre-exposure prophylaxis”. PrEP is one way for people to protect themselves against HIV.

You told me you are taking [PREP TYPE]. Do you know how that kind of PrEP works? Would you like to learn more about that kind of PrEP?

Do you know about the other kinds of PrEP available? Would you like to learn more about other kinds of PrEP?

*[PROVIDE INFORMATION IN TABLE BELOW, DEPENDING ON THE TYPE OF PREP THE PARTICIPANT IS TAKING. YOU CAN ALSO ASK IF THE PARTICIPANT WOULD LIKE TO LEARN ABOUT THE OTHER KINDS OF PREP]*

| **Modality** | **Daily, oral PrEP** | **Event-driven PrEP** | **Long-acting injectable (CAB-LA)** |
| --- | --- | --- | --- |
| What is it? | Daily, oral PrEP involves taking one pill a day. The pill contains two medicines that are also used to treat HIV. It is important to remember though that even though it has some of the medicines used to treat HIV, PrEP does not treat HIV. | Event-driven PrEP is sometimes called “on-demand” PrEP. To protect against HIV, you have totake 2 pills at least 2 and up to 24 hours before you have sex. Then 1 pill 24 hours after your first dose of 2 pills, and 1 more pill 24 hours after that. That means for every time you have sex, you would take4 pills in total.  The pill contains two medicines that are also used to treat HIV. It is important to remember though that even though it has some of the medicines used to treat HIV, PrEP does not treat HIV. | Long-acting injectable PrEP (or CAB-LA) is an injection that protects you from HIV. This medication is injected into your bottom. You would receive an injection when you start, another injection 1 month later, and then would need to come for injections every two months after that. Like the pill option, he injection also contains one of the medicines that is also used to treat HIV. It is important to remember though that even though it has some of the medicine used to treat HIV, injectable PrEP does not treat HIV. |
| How does it work? | By having these medicines in your body BEFORE you are exposed to HIV, they can work to keep the virus from taking hold in your body. | | |
| How well does PrEP work? | When taken every day, daily oral PrEP can reduce the risk for HIV infection by more than 90%. However, daily, oral PrEP may not work as well or at all if it is not taken consistently. | When the 4 pills are taken as directed for event-driven PrEP, it can reduce the risk for HIV infection by more than 90%. However, event-driven oral PrEP may not work as well or at all if it is not taken consistently. | When the injections are taken as directed, injectable PrEP can reduce the risk for HIV infection by more than 90%. However, injectable PrEP may not work as well or at all if the injections are not received on time. |

Do you feel that PrEP is safe? *[PAUSE]*

PrEP is very safe. A few people may experience some start-up symptoms such as upset stomach, diarrhea, or headaches but these are usually mild and go away within the first few weeks. Other side effects are very rare.

Why is it important to get tested regularly for HIV before and while taking PrEP?

*[PAUSE ]*

Testing regularly while taking PrEP is important to make sure that you are still HIV negative. If you become HIV positive, then you need to take ART to keep you and your partners healthy. Remember, even though PrEP has some of the same medications we use to treat HIV, PrEP DOES NOT treat HIV.

Do you know about for how long someone should stay on PrEP?   *[PAUSE]*

PrEP is not lifelong. It is only for as long as you need it to help you stay HIV negative.

[ACTIVITY B: PREP ADHERENCE OVERVIEW

Now let’s talk a little about adherence to PrEP. What do you think of when I say adherence? What does it mean to you? *[PAUSE]*

When I say adherence, I mean taking the PrEP pill as it is prescribed, or if taking injectable PrEP, continuing with receiving your injections on time.

[PROCEED WITH TYPE OF PREP PARTICIPANT IS TAKING]

*[DAILY ORAL]* In this case, perfect adherence would be taking the pill every single day. Studies have shown that PrEP works very well for preventing HIV if it is taken every day.

*[EVENT-DRIVEN]* In this case, perfect adherence would be 2 pills 2-24 hours before you have sex. Then 1 pill 24 hours after your first dose of 2 pills, and 1 more pill 24 hours after that (4 pills in total).

*[LONG-ACTING INJECTABLE]* In this case, perfect adherence would be receiving 2 injections four weeks apart, and then receiving injections every 8 weeks after that, to continue your protection.

PrEP may not work as well or at all if a person does not adhere to it as directed.

What thoughts do you have about adhering to your PrEP as I just described? Can you think of any reason why it might be difficult to adhere to your PrEP?

[PAUSE; IF THE PARTICIPANT IS STRUGGLING, YOU CAN PROVIDE THE PROMPTS BELOW]

Sometimes people can struggle to adhere to PrEP because of:

- Not feeling they still need PrEP: HIV risk changes over time, they may not think they are at risk of acquiring HIV because they are in a steady partnership with someone they know doesn’t have other partners and has tested HIV negative.
- Travel: for work or pleasure; they can forget to bring their PrEP if they are taking PrEP pills or forget to take the pills because they are busy with other things. They may also forget to attend their clinic visit if they are traveling and fail to come another day.
- Forgetting: for a reason other than travel, such as a hectic schedule or normal pressures of life.
- Possible side effects: those side effects are rare, sometimes people can stop taking their PrEP because they are experiencing side effects.
- Partner/ family conflicts: sometimes pressure from family can make it very difficult to remember to PrEP or someone may not feel like taking it.
- School or work demands: sometimes someone can feel too busy because of school or work pressure and forget to take it, or stop taking it without noticing.
- Privacy concerns: sometimes people may not want others to know that they are taking PrEP so it can be difficult to find time to take PrEP.
- Lack of social support: sometimes it can be difficult to take PrEP if it feels like someone is alone and there is not anyone there to help them.

*[DOCUMENT POSSIBLE BARRIERS IN THE SESSION NOTES; NOTE MODULES THAT MAY BE HELPFUL FOR THE PARTICIPANT, EX: PSYCHOSOCIAL]*

[*IF PARTICIPANT REPORTS BARRIERS TO PREP]*

Thank you for sharing that with me. I want to remind you that I will be here to help you if any of these challenges occur. We will talk a little bit today about how to address some of those barriers.

[ACTIVITY C: PREP REMINDER PLAN]

***DAILY ORAL CALENDAR: APPENDIX 1***

Now that we have reviewed the importance of taking the PrEP medication daily, let’s start talking about how you will take PrEP. Think about an average week in your life.

Please fill out this calendar with the typical weekly schedule that you keep.

*[PROVIDE TIME FOR THE PARTICIPANT TO COMPLETE THE PLANNER.]*

Thank you for filling out the planner. The idea of having you complete this planner is to get you to start thinking about things that you do on a daily basis. This way we can review your daily schedule for cues that may remind you when it is time to take your pill. Is there anything that you do most every day that could serve as a cue or signal for you to take your pill?

*[PROVIDE TIME FOR THE PARTICIPANT TO DISCUSS THEIR DAILY PLANNER. IF PARTICIPANT HAS TROUBLE IDENTIFYING DAILY ACTIVITIES. PROBE ONLY IF NECESSARY]*

- Are there other activities that happen with some regularity throughout your day (e.g., do you have a morning or nightly routine)? These might be major activities (going to work and/or school, picking up a child from school), or minor activities (watching a certain TV show, listening to radio program, or brushing your teeth before bed)?
- Does your typical weekend day differ from your typical week day? How?

*[Help participant identify cues for daily dosing e.g., can the participant set an alarm on their phone or otherwise to remind them to take it?]*

***EVENT-DRIVEN***

Now that we have reviewed the importance of taking the pills as described for event-driven PrEP, let’s start talking about how you will take PrEP. Remember, with ED PrEP, the idea is that you are taking PrEP around the times that you are having sex. Think about an average week in your life. Are there certain days of the week that you are more likely to have sex? Or activities you do around the time you have sex (maybe go to a bar or a party)? If possible, try to indicate when you most often have sex, and what, if anything, you commonly do on those days. We know that it can vary from week to week, but this is just to help us get an idea.

*[Provide time for the participant to complete the planner.]*

Thank you for filling out the planner. The idea of having you complete this planner is to get you to start thinking about things that you  do around the times you have sex and when you might need to take PrEP for sexual activity. This way we can review your schedule for cues that may remind you when it is time to take your pill.

Is there anything that you do most before you have sex, that could serve as a cue or signal for you to take your pill? Do you find you can typically predict when you are going to have sex?

*[PROVIDE TIME FOR THE PARTICIPANT TO DISCUSS THEIR DAILY PLANNER. IF PARTICIPANT HAS TROUBLE IDENTIFYING DAILY ACTIVITIES, PROBE IF NECESSARY]*

- Are there other activities that happen with some regularity throughout your week  ? Does your typical weekend day differ from your typical week day? How?
- Do days you have sex typically differ from days you don’t have sex? How?

Help to participant identify cues for event-driven  dosing (e.g., can the participant set an alarm on their phone or otherwise to remind them to take it on days before they think they are likely to have sex and for the two days after sex?).

***CAB-LA [APPENDIX 2]:***

Now that we have reviewed the importance of receiving your PrEP injection on time, let’s start talking about how you will remember to come to the clinic for your injections. Think about an average month in your life. Are there some days of a week or month that you know you will be out of town or unable to come to the clinic? Are there other days that are typically more flexible and would work better for you to come to clinic? Let’s focus on a typical month.

Please fill out this calendar with any common or recurring events (pay rent, receiving a paycheck, visiting family)?

*[PROVIDE TIME FOR THE PARTICIPANT TO COMPLETE THE PLANNER.]*

Thank you for filling out the planner. The idea of having you complete this planner is to get you to start thinking about things that you do on a monthly basis. This way we can review the schedule for cues that may remind you when it is time to come for your injection.

Is there anything that you do most every month that could serve as a cue or signal for you to come for your injection? Remember that, after the second injection, you only have to come every OTHER month, but just thinking now about cues that may help you.

*[PROVIDE TIME FOR THE PARTICIPANT TO DISCUSS THEIR  PLANNER.]*

[ACTIVITY D: Potential Barriers to PrEP Adherence ]

Now that we have discussed your plan for taking PrEP and what you can do to remind yourself, to take it, I also want to talk a bit about whether or not you anticipate any barriers to adhering to your PrEP as I described earlier. What may be some things that could make it harder for you to take your PrEP the way you planned?

[Probes if needed]:

- Do you ever stay overnight somewhere other than your home?
- What might disrupt your schedule (e.g., kids staying home sick from school, socializing, travel)?
- Have you told anyone, including roommates, family, or partners, about your PrEP use? Do you try and hide your pills or are they out in the open?
- What about if you stopped having sex, do you think you would still want to come in for PrEP care/continue to take your PrEP?
- What are some things you could do to try and plan for disruptions to your schedule?
- What are some of the reasons you haven’t told others about your PrEP use?
- What are some of the reasons you may choose to stop taking PrEP?

Now, I want you to think about some of the things that make it EASIER to take PrEP. What are some of the things you do to help yourself remember?

[Probes if needed]:

- What motivates you to take the pill (or come in for the injection)?
- Is there anyone you’ve told that helps to remind you?
- Any tools you’ve used or come up with, like an alarm on a phone or linking PrEP to a daily activity like tooth brushing or a meal?

[ACTIVITY E: ROLE PLAY]

*[To help the participant become aware of their automatic thoughts and resulting emotions the navigator may role play different situations with the participant, pausing at points to identify what automatic thoughts are occurring.  This exercise can also be useful to allow participants to practice skills they have been building to address identified problems.]*

From working at the clinic for a long time, I have some examples from other patients about what can work to help resolve some of the PrEP challenges. Would it be ok if I shared a strategy with you?

Sometimes at the clinic after someone has identified a barrier to PrEP, we work through the scenario together and role-play so that the participant can practice. Do you think this could be helpful for you?

*[Here are a few tips to help with introducing role playing with a participant]:*

- *[Pick a concrete situation that occurred recently for the participant or a brief scenario that they think might happen to them.]*
- *[Ask the participant to provide some background on the target person for the role play.]*
- *[Have participants play the target person, so they can convey a clear picture of the style of the person and the counselor can model effective strategies for the interaction. Then reverse the roles for subsequent role-plays. (Note, if the role play is simple and brief, the participant can play themselves. Ex: asking about side effects in the clinic).]*
- *[Role-plays should be thoroughly discussed afterward.]*
- *[Navigators should praise any effective behaviors shown by participants and offer clear, constructive criticism (example below).]*

**Examples scenarios:**

- A partner who finds the participant’s pills and confronts them.
- A family member who doesn’t understand the important of PrEP adherence.
- A clinic staff member who is rude to the participant.
- A family member or friend who doesn’t approve of the participant’s PrEP use.
- Asking the health care provider about side-effects.

**PART 3: GOALS / HOMEWORK FOR NEXT SESSION**

- (if oral PrEP) Try and keep track of your PrEP use for a full week – I want you to think about (and maybe write down) a time where you noticed that it was easy to take PrEP – maybe you linked it to something in your routine. Then, think about a time where you forgot (or almost forgot) a dose – think about what was happening (perhaps you had gone out for the weekend, or you had gone to a party).
- (if injectable PrEP) think about how you are going to remember the date of your next appointment – perhaps you can write it down and post it in your home somewhere you look frequently? Or you link to another activity that you know would be happening around the same time? I want you to come back and tell me next time what your strategy was.

- (if injectable PrEP) think about how you are going to remember the date of your next injection – perhaps you can write it down and post in your home somewhere you look frequently? Or you link to another activity that you know would be happening around the same time? I want you to come back and tell me next time what your strategy was.

**PART 3: SUMMARY**

Today we talked more about PrEP, how you can remember to take your PrEP and what you can do in case you come across any barriers to taking your PrEP. Next time that you come in for your PrEP visit we will discuss how you were able to accomplish the goal we set together. Don’t worry if you come across challenges, challenges happen. Remember I am here to support you in your PrEP journey.

**PrEP Planner Example Weekly Planner**



Week of:  N/A

| Monday | Tuesday | Wednesday | Thursday |
| --- | --- | --- | --- |
| Wake up 6      Take tea    Arrives to Work  8:00 am        Knocks at 5    Reaches home 7  Dinner | Wake up 6 | Wake up 6 | Wake up 6        Arrives to work at 2 pm |
| Friday | Saturday | Sunday | Other notes |
| Wake up 6                      Social football | Wake up 7                      Drinking with friends  Sleeping out | Wake up 7      Church morning        Social football |  |

**Appendix 1: Weekly Planner** (use for daily, oral PrEP  and event-driven PrEP).



Week of:

| Monday | Tuesday | Wednesday | Thursday |
| --- | --- | --- | --- |
|  |  |  |  |
| Friday | Saturday | Sunday | Other notes |
|  |  |  |  |

**Appendix 2:** Monthly PrEP Planner (use for CAB-LA/long-acting injectable)

| Sunday | Monday | Tuesday | Wednesday | Thursday | Friday | Saturday |
| --- | --- | --- | --- | --- | --- | --- |
| Sunday | Monday | Tuesday | Wednesday | Thursday | Friday | Saturday |
| Sunday | Monday | Tuesday | Wednesday | Thursday | Friday | Saturday |
| Sunday | Monday | Tuesday | Wednesday | Thursday | Friday | Saturday |
| Sunday | Monday | Tuesday | Wednesday | Thursday | Friday | Saturday |

### **MODULE C: BARRIERS AND MOTIVATORS TO PREP ADHERENCE AND PROBLEM SOLVING**

*[NOTES FOR SYSTEMS NAVIGATOR]*

*[As always, it may be helpful to review the participant’s notes from the introductory session before you begin. There you can find information about potential barriers to PrEP adherence that the participant may face. For example, if the participant travels often for work, you may want to note that before you begin this module today. It may also be helpful to note if the participant has already gone through the “PrEP 101 and Adherence” module and if you wrote in your session notes what adherence meant to the participant and/or if they had good understanding of adherence.]*

*[The goals and objectives for the PrEP Barriers and Problem Solving module are:]*

- *[Discuss potential barriers to adherence.]*
- *[Brainstorm possible solutions to these potential barriers.]*
- *[Set homework for next session.]*

*[Like with all modules it will be good to practice your motivational interviewing, reflective listening, empathy and other tools to discuss these topics.]*

**PART 1: INTRODUCTION**

Today we will focus on learning more about adherence to PrEP and problem solving in case you have any difficulties adhering to PrEP.  Adherence to PrEP means taking PrEP in a way that will keep someone healthy and protected from HIV.

**PART 2: ACTIVITIES**

[ACTIVITY A: MOTIVATIONS FOR TAKING PREP]

[*Note: you may not need to complete this activity if you have a good knowledge of the participant’s life and what motivates them, either from Module A or from your previous interactions with them. Or you can paraphrase this part that instead of learning about the them you want to show that you’ve been listening to their motivations by referencing your session notes here.]*

I want to start by learning a bit more about you and your goals for your health. Can you please share with me a bit about why your health is important to you?

What kind of activities do you do to maintain your health? How often do you do these activities?

Why is taking PrEP important for you? How often are you taking PrEP?

[ACTIVITY B: ADHERENCE REFRESHER]

*[Note: you may not need to complete this if the participant has completed PrEP 101 Overview and Adherence or seems to have a good understanding of adherence.]*

Can you tell me how you think about adherence to PrEP? What it is and what does it mean to you?

*[PAUSE to allow participant to respond. If adherence information is incorrect, remind the participant of what adherence looks like for their PrEP modality. If they are correct, encourage them for their knowledge and ask if they have any questions about adherence. Alternatively, you could explore their health and health goals more broadly, particularly if someone reports that remaining healthy is important to them]*

- Why is your health important? What activities do you do to maintain your health?
- Is taking PrEP important for you? Why? How often are you taking PrEP?

[ACTIVITY C: POTENTIAL BARRIERS TO PREP ADHERENCE]

***Appendix 1: Potential barriers to PrEP***

[*Note: all the prompts are meant as a suggestion to help the participant tell you about how they take their PrEP, they are not “required” questions that we want the participant to answer. Feel free to ask the participant these questions in your own way, however you can understand the information. You can also use the checklist in the appendix if that will be easier for the participant than discussing these questions. Change questions based upon when the participant started taking PrEP.]*

- There are lots of things that can make it difficult to coming to the clinic on time or taking your pills on time. What types of things might get in your way of going to the clinic?
- Once you make an appointment are there ways you can remind yourself to go or have some else remind you?
- Some other clients have shared barriers with me that they have experienced in the past. Can I share some of those examples with you, so that we can talk about what challenges you might face as well? *[If yes, go to appendix 1]*

*[Prompts for PrEP appointments]*

1. How often are your PrEP appointments?
2. How do you plan to get to your appointments?
3. Does anyone remind you to go to your appointments?
4. Do you know where to go for your PrEP visit? Is there anything that isn’t clear about the clinic and how might get to your appointment?
5. Do you experience anything difficult when you get to the clinic?
6. Do you feel free to ask any questions you might have to the nurse?
7. Do you feel comfortable to move around the clinic?

*[Use MI prompts as seems relevant here, particularly if they say they don’t feel comfortable in the clinic.]*

1. Think about your next PrEP visit. What questions do you want to ask about the medication? About side effects?

[*Anticipating challenges/prompting consideration]*

1. How do you carry the PrEP?
2. Where will you keep your PrEP?  If you leave home, will you take it with you?
3. What happens if you run out of PrEP?
4. When do you want to take your PrEP?  How will you remember to take it?
5. How would you feel if you forgot to take your PrEP one day?
6. What would you do if you slept through a dose? What if you were traveling and forgot your PrEP?
7. What if you side effects were so bad you didn’t feel like taking it?

[ACTIVITY D: PROS AND CONS TO STAYING ON PREP]

***Appendix 2: Pros and Cons***

*[You could use this activity for a participant that doesn’t seem very motivated to stay on PrEP or seems like they might not be able to overcome the challenges of taking PrEP that they are facing. It could also be a helpful way to demonstrate and explore ambivalence if it comes up in your time together].*

I can see that you have a lot of things that are important to you, and it might not be clear why you want to stay on PrEP, especially if you have many challenges to adhering to PrEP. Can we spend a bit of time talking about the good things and the bad things about staying on PrEP?

[*IF YES, go to Appendix 2*].

[ACIVITY E: MAKING A PLAN FOR THEIR NEXT APPOINTMENT]

We have talked about adherence being specific to taking your PrEP as prescribed. However, adherence also includes anticipating issues that may not be directly related to taking [a pill/receiving an injection] but that can have a great influence on being able to take [the pill/receive the injection].

For example, if I have work to do on the day I have scheduled my appointment, I may not be able to get my PrEP medication refilled on time and so I may miss a pill and not be adherent [that day].

I’d like for us to make a plan for your next PrEP appointment. How do you keep track of when your next appointment is? What would you do if you were not able to make that appointment? Do you know where you need to go?

*[once you have made a plan, affirm the participant and document their plan in the session notes.]*

It is great that you are making a plan and thinking about how you can be consistent in achieving that goal. What positive things can you say to yourself to encourage you to go to the clinic?

[ACTIVITY F: USING THE DECIDE MODEL TO FIND SOLUTIONS]

[*You might want to use this activity to work through a barrier to PrEP adherence a participant has identified in the earlier activities. You can talk through the approach outlined below with them.]*

Can I share with you a strategy that helps sometimes when we face a problem? It is called “decide” and it helps to make problems seem more manageable. Let’s pick a challenge to adherence that you identified earlier.

**D=Define the problem:** you might say “I need to figure out a way to keep my PrEP appointments”

**E=Explain ways to change it:** You might say “I need to think about is keeping me from keeping my appointments.”

**C=Choose the best option:** you might say “I need to pick the best option for me”

**I=It’s time to act:** you might say “I need to try out my choice”

**D=Did it work?** You might say “Am I OK with the results?”

**E=Explore other solutions to my problem:** you might say “If the results are not OK with me, I need to try another option.”

**PART 3: GOALS / HOMEWORK FOR NEXT SESSION**

[*Since this is a goal-heavy module, you may not need to set a specific goal for the next session with the navigator. You can just refer to the goals they have stated previously. If they haven’t set a new goal, work with them here to do so.]*

I would like to learn more about any specific goals you might have for PrEP adherence. For example, a goal for getting appointments can be “I want to make it to all my scheduled appointments!” or maybe it could be “I want to make it to my ***next*** scheduled appointment!”

Do you have any PrEP goals? If so, what are they? [*you can ask or remind the participant here why taking PrEP is important for them*].

Let’s consider 3 steps you can do to improve your adherence and minimize barriers. It may take some work at first, but it can become automatic and part of your routine.

- **A: A**dherence goal – state it!
- **I: I**dentify problems with reaching the goal
- **M: M**ake a plan to overcome the problems and develop a back-up plan.

*[Alternative goals you can set might include]*

- Document barriers that come up (if any) to take your PrEP and/or get to the clinic for a PrEP refill. Document any strategies you used to mitigate them.
- Make a SMART adherence goal with the participant.

**PART 4: SUMMARY OF MODULE**

- Barriers to PrEP adherence
- Pros and cons to staying on PrEP
- Problem-solving techniques the barriers (role-playing, decide)

**Appendix 1: BARRIERS TO PREP**

| Potential Barrier | Does this sound like a challenge you might experience? | |
| --- | --- | --- |
|  | Yes | No |
| I don’t have time to go to the clinic. |  |  |
| I don’t have money for transport to go to the clinic. |  |  |
| I don’t have the energy or interest to go to the clinic |  |  |
| If I miss an appointment, I will be too embarrassed/ ashamed to go back |  |  |
| I am worried about the unwanted side effects or complications of taking PrEP |  |  |
| My drinking will make/has made it difficult to remember to take my PrEP. |  |  |
| I don’t have family or friends to help me remember to take my PrEP or to support me. |  |  |
| I don’t want anyone to know I am taking PrEP |  |  |
| I travel often (for work) so it can be difficult to remember to take my PrEP or to get to the clinic in good time. |  |  |
| Doctors will treat me poorly / Doctors have treated me poorly in the past |  |  |

**Appendix 2: Pros and cons to staying on PrEP**

|  | **Pros/Benefits** | **Cons/Costs** |
| --- | --- | --- |
| **Taking PrEP** |  |  |
| **Not taking PrEP** |  |  |

### **MODULE D: PREP DISCLOSURE**

*[NOTES FOR SYSTEMS NAVIGATOR]*

*[Remember that it is not required for a participant to disclose to others that they are on PrEP. This module is designed for participants who have decided that they want to disclose that they are taking PrEP to someone in their life, or who are interested in disclosing their PrEP use but want to learn more about what is involved in disclosure. Remember that, for some people, disclosing PrEP use can be an enormous help in remaining engaged in PrEP care and adherent to PrEP medications – the person they disclose to may help to remind them or encourage them to keep appointments or to take medications. If someone is not sure about disclosing PrEP, you may consider offering how or why disclosing could be helpful to keep the participant safe and protected from HIV.]*

*[The goals and objectives for the PrEP Disclosure module are:]*

- *[Help the participant understand strategies for disclosing PrEP use.]*
- *[Help the participant feel comfortable about the option to disclose their PrEP use, as a potential strategy to help keep them adherent to PrEP care.]*
- *[Set goal for next session (can be about disclosure but it doesn’t have to be).]*

*[It may be helpful to review your notes about role-playing in the manual before you begin this session.]*

**PART 1: INTRODUCTION**

Disclosure, or telling someone you are taking PrEP in this case, is sometimes difficult. You may be afraid that someone might misunderstand and think that you have HIV, for example. Or perhaps you would fear they would judge you or think differently about you as a person. There are many other reasons why you may not – or may – want to tell someone you are taking PrEP. Sometimes, having someone else know about your taking PrEP can help you to keep appointments or to remind you to take medications on time. We will talk through disclosure situations in this session.

A few questions to get us started:

- What is your first reaction to telling others you are on PrEP?
- Have you already told anyone you are on PrEP? How did that go? Would you do something differently?
- Is there someone you would like to tell you are on PrEP?

**PART 2: ACTIVITIES**

[ACTIVITY A: PROS AND CONS TO DISCLOSURE]

**Refer to Appendix 1** “Pros and Cons to PrEP Disclosure”

[*Note: this activity could be helpful for someone who has discussed that someone finding their PrEP would pose a challenge, or that might be interested in disclosing but isn’t sure].*

Let’s talk about the possibility of telling someone that you are taking PrEP. It is your choice to let someone know whether you are taking PrEP or not. Oftentimes, it may not be possible to keep it from others, especially people you live with or spend a lot of time with.

How do you feel about telling people that you are taking PrEP?

Is there someone you want to disclosure your PrEP to?

What could be some of the benefits of disclosing PrEP? What about the costs?

[*option to complete pro/con table with participant].*

[ACTIVITY B: SAFE TALK]

If you are struggling with how to tell someone that you are taking PrEP, preparing to have the conversation can sometimes help to make it a bit easier. There is an acronym, T.A.L.K., that can help guide you through the process of planning to disclose your PrEP use. TALK stands for “Timing Assertive Communication Location Know What to Say”.

**TIMING** Choose an appropriate time to talk with your person. If the person that you need to talk with has a busy lifestyle, then it might be easier for you to set a meeting time. This way, each person’s attention can be focused on the issue.

**ASSERTIVE COMMUNICATION** Clearly tell the person how you feel and what you want or need by being honest and direct. Think carefully about your relationship and pay attention to others’ responses. Depending on the specific person, you might have to address issues differently. Remember to use “I” statements, take deep breaths, keep a reasonable tone, and actively listen to the other person.

**LOCATION** Choose a quiet place where you cannot be interrupted or overheard by others.

**KNOWING WHAT TO SAY** Think about what you want to say in advance by sorting out your own feelings about the issue before talking with the other person. You might find that making a list or writing a letter of your thoughts and feelings will help you focus.

You have control over whether you tell people, who you tell, and how you tell them. Think about what is best for you and make sure you are ready. We can come up with a plan to disclose together, if you think that might be helpful [*use the guide in Appendix 2].*

[ACTIVITY C ROLE PLAY PrEP DISCLOSURE]

Now we are going to have you practice telling someone you are on PrEP by doing some role-playing, even if you aren’t ready to tell someone yet. Choose someone who you may want to tell about PrEP in the future. Let me know who it is and provide me with some details about where the conversation is taking place. The more details you provide, the better. I will then pretend to be the person and react as I think the person would.

*[Allow time for the participant to come up with an idea. If they are struggling then you can provide an example like: maybe you are interested in disclosing to your wife, but you are afraid she might say something like “But I am faithful to you, why do you need to be taking PrEP?” or “PrEP can make us sick”, or “you must have HIV”.]*

*[Once the participant has selected a person for disclosure, conduct the role playing activity with the person the participant selected for disclosure, and then use the questions below to guide a debrief about the role play].*

*[Possible questions to ask the participant]*

What was the most challenging thing about this role-play?

What was easier or harder than you thought it would be?

What surprised you going through this role-play?

What is one thing that you liked about what occurred in this role play?

What is one thing you would want to change when disclosing in real life?

[ACTIVITY D: MAKE A DISCLOSURE ACTION PLAN]

***Refer to Appendix 2:*** *“Action Plan: Disclosure” handout.*

You have said that you are interested in telling someone that you are taking PrEP. Let’s develop an action plan to outline what steps you will take.

Think about the specific person whom you would like to disclose your PrEP use to. Use this worksheet to think through the reasons why you want to disclose to that person. Then use this form to plan out the process. Bring together all of the elements of disclosure we talked about today. Decide when you would like to tell them, where you will have the talk, what you will say, and how you will do it. Finally, think about what the potential costs and benefits of disclosing to this person would be. Remember when thinking about costs and benefits, think primarily about how disclosing your PrEP use to them will affect you and your life, including your ability to stay on PrEP.

**PART 3: GOALS/ HOMEWORK FOR NEXT SESSION**

Think about what you’ve learned during this session about disclosure and whether it would benefit you or not to disclose your PrEP use to someone. Come prepared to discuss your feelings on disclosure next time we meet.

If you have made up your mind that you would like to disclose to someone – try and use the action plan, we developed today or make a new one for whomever you want to disclose to. Think about who, where, how, what, and when you may disclose. You can bring this back in next time we meet, or we can just discuss how your conversations went

**PART 4: SUMMARY OF MODULE**

Have the participant discuss what some of the good and bad parts of disclosure may be for them.

Three activities to choose from *[You don’t have to have done all three*!]

1. Discussion of “SAFE TALK” and some best practices for disclosure.
2. Role-playing disclosure
3. Creation of Disclosure Action Plan
4. Assignment of homework

**Appendix 1: Pros/benefits and cons/costs to Disclosing PrEP use**

|  | **Pros/Benefits** | **Cons/Costs** |
| --- | --- | --- |
| **Disclosing your PrEP status** |  |  |
| **Not disclosing your PrEP status** |  |  |

**Appendix 2: Disclosure Action Plan**

**ACTION PLAN: DISCLOSURE**

Think about one specific person to whom you would like to disclose your PrEP use. Let’s use this worksheet to think through the reasons why you might want to disclose to that person. Then use this form to plan out the process.

List all the reasons **why** you want to disclose to _____________________[*name of person]*

**What** will I say?

**Where will I say it?**

**When will I have this conversation?**

**How will I do it?**

**Potential costs?**

**Potential benefits?**

### **MODULE E: SEXUAL HEALTH (STI PREVENTION)**

*[NOTES FOR SYSTEMS NAVIGATOR]*

*[It may be helpful to review the participant’s folder before this module with particular attention on what the participant’s motivation for taking PrEP is. This may make it helpful to remind them about why they may want to avoid STIs. For example, if they said they want to stay on PrEP to protect their wife from HIV, or to stay healthy themselves, you can use these same motivations as reasons it can be important to protect themselves against STIs.]*

*[The goals and objectives for the Sexual Health module are]:*

1. *[Review information on sexual risk reduction*]
2. *[Learn about different levels of STI risk and options for reducing risk.]*

**PART 1: INTRODUCTION**

Today we are going to talk about the different levels of risk associated with different sex behaviors. Talking about sex is sensitive and can be embarrassing for some people. However, I want to remind you that we only need to talk about what you think will be most helpful for you. If at any point you want to move on we can do so. I also want to remind you that everything we discuss here today will remain confidential.

**PART 2: ACTIVITIES**

[ACTIVITY 1: HEALTHY SEXUALITY]

As part of our discussion today, we will explore a different aspect of HIV and other STI prevention: sexuality.

Can you tell me what you think sexuality means? How would you define sexuality?

*[Give enough time for the client to respond]*

Sexuality can mean who you are attracted to, your sexual feelings about other people, the type of sex activities that you do, or even how you feel about sex.

Some of our opinions and beliefs about sex are influenced by our society, community, friends, and family. Let’s spend some time talking about various views of sexuality among men and women.

1. How do you and your community view women and their sexuality?
2. How do you feel (what do you think) about that?
3. How do you and your community view men and their sexuality?
4. How do you feel (what do you think) about that?

Thank you for all your thoughtful answers.

Healthy sexuality can also include:

- Having the sexual knowledge and expertise to feel comfortable with yourself.
- Having the confidence to express yourself in your sexual relationships.
- Being comfortable with how you view your own sexuality and sexual behavior.
- Being knowledgeable and responsible regarding safer sex for you and your partner or partners.
- Being comfortable with your own body and body image.

[ACTIVITY 2: STI RISK]

Now, let’s talk about that we mean when we say sexual risk. Even though you are taking PrEP, which can help protect you from HIV, you still need to consider protecting yourself from other sexually transmitted infections. Sometimes these are called “STIs” for short. Remember, PrEP only works to protect against HIV if taken correctly, and it does not protect against other STIs.

Which STIs have you heard of before?

*[let the participant respond before you offer names of the STIs]*

Some of the most common sexually transmitted infections include:

- Syphilis
- Gonorrhea
- Chlamydia
- Herpes

*What do you know about these STIs?*

*[let the participant respond before you offer information about. Remember to be kind and to continue with motivational interviewing, gently correcting any misinformation that the participant might share with you].*

STIs can present with a variety of symptoms, from a painful ulcer to a painless rash, or discharge from your penis and painful urination. You can have an STI and not have any symptoms – this doesn’t mean the infection is any less serious and it can still be transmitted to other persons even without symptoms. That also means that you can still become infected from someone even if they don’t show any symptoms.

Most STIs are treated with medicine and can be cured – even if there are no symptoms, if a person has tested positive for an STI, they would still require treatment. If an STI goes without treatment, it can cause longer term complications for you and your partners, including scarring, systemic illness (i.e. effecting things other than your genitals), and in some cases, infertility or the inability to have children.

Do you have any questions about why PrEP doesn’t protect you from STIs?

[ACTIVITY 3: SEXUAL RISK ]

Remember, I am not here to judge you, so it is helpful if you can be honest so we can talk about your STI risk.

*[You don’t need to say this next part if you already explained it in Activity 2]*

Even though you are taking PrEP, which can help protect you from HIV, you still need to consider protecting yourself from other sexually transmitted infections. Sometimes these are called “STIs” for short. Remember, PrEP only works to protect against HIV if taken correctly, and it does not protect against other STIs.

When we talk about risk, we are talking about the risk (or chance) that someone may get an STI from a particular behavior. Talking about sex is sensitive and can be embarrassing for some people. Like I mentioned earlier, what is discussed here today will remain confidential.

There are different levels or risk associated with different sex behaviors.

- What do you think your current level of risk for an STI is?
- Why do you think that is so?
- What kind of behaviors do you think you are engaging in that put you at a higher risk for an STI?
- What about behaviors that might lower your risk for an STI?

Here is some information about safer sex options that you can provide to the participant if you think it might be helpful:

- Vaginal sex without a condom is a very high-risk behavior for HIV and other STIs. Do you know why that is?
- Having anal sex without a condom also a very risky type of sex.  Why do you think this is?
- A safer option can be to have vaginal or anal sex with a condom.  Because a condom is a barrier, if you use it properly from start to finish, it greatly reduces transmission and infection of STIs, including HIV. Many people find using lubricants makes sex with a condom more pleasurable.  Have you ever tried to use lubricants?
- Oral sex is a safer alternative – though you can still transmit STIs with oral sex if there is no barrier and if there is any blood from sores or cuts, it can still transmit HIV.
- Another safe alternative is non-penetrating sex activity – this is touching your partner and having them touch you. Keep in mind that some STIs can be spread just through skin-skin-contact, especially herpes and syphilis, and you want to make sure you and your partner don’t have open cuts, sores, or ulcers.

*[Questions you can ask the participant]*

- Do you have any questions about any of the behaviors and their levels of risk?
- Do you think you are still at the same level of risk as when I asked you before? Were you surprised by any activities that are more or less risky?
- In what ways do you think taking PrEP might impact your sexual activity? How might it change your desire or willingness to use condoms?
- Do you think PrEP makes sex “less risky”? Why or how (or why not)?
- Another discussion point when it comes to safer sex is thinking about who you are having sex with – are they having sex with other people as well? Have they been tested for HIV? In general, do you talk to your partners about HIV testing history prior to having sex?
- Does your behavior change depending on who you are having sex with (e.g. not using a condom with a main partner versus using a condom with a casual partner)?

*[if the participant is participating in risky behaviors]*

- What do you think your risk level is?
- Is there anything about your risk that you might want to change?

Is there any information today that was not clear?

[ACTIVITY 4: SAFE TALK]

Part of healthy sexuality also means being open and honest with your sexual partners about your safer sex decisions.   You may decide that you want to talk to your girlfriend, wife or partner about using PrEP at some point. This might seem a bit difficult, but if you prepare yourself, it will be easier. You can use the “Safe TALK” strategy to help you prepare:

**TIMING** Choose an appropriate time to talk with your person. If the person that you need to talk with has a busy lifestyle, then it might be easier for you to set a meeting time. This way, each person’s attention can be focused on the issue.

**ASSERTIVE COMMUNICATION** Clearly tell the person how you feel and what you want or need by being honest and direct. Think carefully about your relationship and pay attention to others’ responses. Depending on the specific person, you might have to address issues differently. Remember to use “I” statements, take deep breaths, keep a reasonable tone, and actively listen to the other person.

**LOCATION** Choose a quiet place where you cannot be interrupted or overheard by others.

**KNOWING WHAT TO SAY** Think about what you want to say in advance by sorting out your own feelings about the issue before talking with the other person. You might find that making a list or writing a letter of your thoughts and feelings will help you focus.

Tell your partner some of the things you have learned about STIs and HIV. It’s also important to negotiate and listen to your partner. Keep in mind that it’s not only your right, but also your RESPONSIBILITY to make decisions that you will help you stay healthy. It’s very important to know what you will say in response to your partner‘s questions, complaints, or efforts to change your mind. You can anticipate their reaction, which will help you be more prepared.

[ACTIVITY 5: HEALTHY SEXUALITY ROLE PLAY]

Sometimes it can be helpful to role play healthy sexual behaviors, such as using a condom. Can you think of a situation where it might be helpful to practice a healthy sexual behavior?

[*If they struggle to come up with an example, you can provide the following]*

What if your partner says…

- I am faithful to you, we don’t need to use a condom.
- Condoms are not effective.
- You are on PrEP, shouldn’t that protect you and I from all STIs?

How would you respond to these statements by your partner? Let’s practice.

[ACTIVITY 6: CORRECT CONDOM USE]

Condoms, when used correctly, are an effective approach to preventing pregnancy and most STIs. For condoms to work they must be worn throughout the sexual contact – not just at “the end.” In addition, the condom needs to be put on correctly to reduce the risk of it tearing or breaking. Here in the clinic we have a model that we use to demonstrate how to correctly use a condom. It has been helpful for other clients to observe the demonstration of me correctly placing the condom on the model. Would it be helpful for you if I demonstrate?

**PART 3: GOALS FOR NEXT SESSION**

*[Ask the participant to try some of the sexual risk reduction strategies before the next session. Ask them to make a note of any challenges they faced in making these changes before your next session so that you can discuss the changes with them when they return.*]

Do you think you need to change anything about how you are currently having sex? What are some ways you think you can reduce your sexual risk?

What challenges do you think you will face in making these changes? How will you address these challenges? *[Give suggestions as appropriate]*

**PART 4: SUMMARY OF MODULE**

Today we spent some time talking about healthy sexuality, STI risk and how it differs from your risk for HIV while taking PrEP and how to communicate about all these topics with your sexual partner.

I know it was a lot of information. Do you have any questions for me about the material we covered?

We then set a goal for you to [*recap the goal to the participant]* between now and your next PrEP visit. I am looking forward to your next visit to hear how you made progress toward this goal.

### **MODULE F: CIRCUMCISION**

*[NOTES FOR SYSTEMS NAVIGATOR]*

*[The goals and objectives for the circumcision module are:]*

- *[To provide the participant with information about medical male circumcision, including dispelling any rumors they may have heard about the procedure.]*
- *[To explain the benefits of circumcision regarding healthy sexuality and HIV/STI prevention.]*

**PART 1: INTRODUCTION**

I would like to talk to you about medical male circumcision. What have you heard about circumcision in the past?

- Medical male circumcision is a procedure where the foreskin of the penis is surgically removed under sterile conditions. Men who have not previously been circumcised are eligible to undergo circumcision here at Bwaila.
- Male circumcision can be very helpful for men and boys to stay healthy.

What are some benefits of circumcision that you have heard of in the past?

- Circumcision can help you:
- Have better hygiene (penile).
- Have less chance of acquiring HIV.
- Be more protected from acquiring other sexually transmitted infections.
- Be protected from developing penile cancer.
- Be protected from phimosis and paraphimosis.
- Protect your partners from cervical cancer.
- Protect against urinary tract infections (young men).

Even though circumcision is usually very safe, there can be some risks associated with it. What are some risks associated with circumcision that you have heard before*?*

*[note, if the participant shares many myths at this point in the session, feel free to move right to Activity 3 “myths and misconceptions].*

- Medical male circumcision has its disadvantages, but healthcare providers try their best to ensure that circumcision is done with minimal risks. Still, there are a few risks that can occur:
  - Pain on incision site – healthcare providers ensure that the procedure is pain free and will give you pain medication to alleviate pain after the procedure.
  - Excessive bleeding – healthcare providers ensure that bleeding has stopped/been controlled before you leave the health facility.
  - Infection of the incision site – you are required to inform your provider or report to the nearest health facility to receive medical attention if you notice any signs of infection.

**PART 2: ACTIVITIES**

[ACTIVITY 1: MYTHS AND MISCONCEPTIONS ABOUT MEDICAL MALE CIRCUMCISION]

What else have you heard about circumcision?

What have you heard from your friends or family or in the community about what can happen when you become circumcised?

- Medical male circumcision is a contraceptive method – circumcised men cannot impregnate a woman.
- Medical male circumcision is the government’s strategy for population control.
- Medical male circumcision makes men impotent for life.
- Circumcision makes men to become unfaithful to their partners.
- Medical male circumcision causes penile cancer.
- Medical male circumcision reduces a man’s libido.
- A circumcised man can NEVER acquire HIV or other sexually transmitted infections.
- The government is selling men’s foreskin to other countries for profit.
- Men’s foreskin is being sold to fishermen as bait for catching big fish.
- Once someone is circumcised, they become Muslim.
- Once someone is circumcised, they become of the Yao tribe.

[ACTIVITY 3: MAKE A PLAN FOR CIRCUMCISION]

Now that you have learned more about medical male circumcision, if you are interested in undergoing the procedure, I can help you make a plan to come to the clinic. Would it be helpful to schedule your circumcision appointment around your next PrEP visit?

[*Note, you can assist the participant to make the appointment when they leave the clinic, by reminding them at their next visit, etc.]*

[ACTIVITY 4: ROLE PLAY CIRCUMCISION DISUCSSIONS

Sometimes it can be helpful to practice scenarios around circumcision, so that you are free and comfortable once you are in them for real. Some patients find this strategy helpful, particularly around practicing discussing circumcision with a provider, or informing their wife they would like to undergo circumcision. Would practicing a scenario around discussing circumcision be helpful for you? It can be either of the ones we suggested, or another one that you have thought of on your own.

**Option 1: Meeting your provider at the clinic to schedule your circumcision appointment.**

Even though we have discussed a bit about circumcision, sometimes people still

have questions about the procedure, or they think of new questions once they have left the clinic. Sometimes people can be nervous about talking to a provider once they reach the clinic for their circumcision appointment. Would it be helpful to role play what a first appointment asking about circumcision, might look like? Or maybe we can pretend that you are at coming to the clinic on the day you are getting circumcised? Which scenario do you think would be more helpful to practice? I can pretend to be the patient first and you can be the provider. Then we can switch, and you can pretend to be a patient asking the healthcare provider about circumcision.

**Option 2: Informing your wife or partner** **that you want to get circumcised.**

Even though we have discussed a bit about the benefits of circumcision for your health, sometimes it can be difficult to discuss your decision to undergo circumcision with your wife/partner. Would it be helpful to role play informing your wife/partner that you would like to get circumcised? I can pretend to be you first and you can be your wife/partner. Then we can switch, and you can pretend to be you and I can pretend to be your wife.

[ACTIVITY 5: SAFE TALK]

Now that we have role played telling your wife/partner you would like to get circumcised, it might be helpful to use the SAFE TALK strategy to plan for your disclosure of your plan to get circumcised, or to inform them that you have gone through with it.

**TIMING** Choose an appropriate time to talk with your person. If the person that you need to talk with has a busy lifestyle, then it might be easier for you to set a meeting time. This way, each person’s attention can be focused on the issue.

**ASSERTIVE COMMUNICATION** Clearly tell the person how you feel and what you want or need by being honest and direct. Think carefully about your relationship and pay attention to others’ responses. Depending on the specific person, you might have to address issues differently. Remember to use “I” statements, take deep breaths, keep a reasonable tone, and actively listen to the other person.

**LOCATION** Choose a quiet place where you cannot be interrupted or overheard by others.

**KNOWING WHAT TO SAY** Think about what you want to say in advance by sorting out your own feelings about the issue before talking with the other person. You might find that making a list or writing a letter of your thoughts and feelings will help you focus.

Tell your partner some of the things you have learned about STIs and HIV. It’s also important to negotiate and listen to your partner. Keep in mind that it’s not only your right, but also your RESPONSIBILITY to make decisions that you will help you stay healthy. It’s very important to know what you will say in response to your partner‘s questions, complaints, or efforts to change your mind. You can anticipate their reaction, which will help you be more prepared.

[ACTIVITY 6: PROS and CONS]

**Refer to Appendix 1: Pros and cons table.**

[*If the participant is still unsure about whether they want to go through with circumcision, you can complete a pros and cons table with them].*

It seems like you are still unsure about the benefits and the costs associated with circumcision. Some patients at the clinic have found the strategy of using a “pros” and “cons” table to be helpful when they are thinking through this kind of decision. Would it be

**PART 3: HOMEWORK/GOALS FOR NEXT SESSION**

*[Some options for goals to set with the participant might include:]*

- Schedule an appointment to learn more about medical male circumcision or to have the procedure completed.
- Ask a friend who has been circumcised what their experience was like.
- Think of other pros and cons to circumcision to add to the participant’s pros and cons table.

**PART 4: SUMMARY OF MODULE**

Today we talked about medical male circumcision, what it is and some of the common misconceptions about it. We discussed the benefits for your health and the health of your sexual partners if you choose to undergo circumcision. We set a goal for you to *[repeat goal to the participant]* before your next visit. What other questions do you have about circumcision for me?

**Appendix 1: Pros and cons table**

|  | **Pros /Benefits** | **Cons / Costs** |
| --- | --- | --- |
| **Getting circumcised** |  |  |
| **Not getting circumcised** |  |  |

**MODULE G: SUBSTANCE USE (OTHER THAN ALCOHOL)**

*[NOTES FOR SYSTEMS NAVIGATOR]*

*[The goals and objectives for the Substance Use module are]:*

- *[Contextualize drug use and explain risks for disease spread and drug use.]*
- *[Assess current drug use and potential consequences of this drug use.]*
- *[Assess potential desire to change.]*
- *[Set SMART goal for overall health/drug use to discuss at the next session*.*]*

As a reminder, it is not your job to encourage the participant to stop all of their drug use if that is not a goal the participant has for themselves. If it seems like the participant is only aware of the possibility of stopping their use completely or using drugs in the same way they have been, you can remind them that it is also possible to *reduce* their drug use if they are unable to stop it completely.

**PART 1: INTRODUCTION**

Today I would like to talk to you a bit about substances and drug use. Some people might feel hesitant to talk openly about their drug use because some drugs are illegal and, in some places, drug use can be stigmatized. However, I want to reiterate that everything you share with me is confidential, and that I am not here to judge you. My job is only to help you to be as healthy as you can be.

Can you describe for me what you think of when I say “substances”? What are some substances you have heard of, in the past?

Substances are any kind of drug (like alcohol, marijuana, cocaine, etc.) that can impair your cognition and influence your behavior. Alcohol is definitely an important substance, but today we are going to be focusing on non-alcohol substances.

What do you think of when you hear the term “drug use”?

Some people use substances/drugs infrequently (for example, once per month), while others can use the substances often or even every day. People can also use different kinds of substances, which can have different impacts on your mood and/or your behavior. It is important to understand how the use of substances can influence your risk of contracting an STI and/or other infectious diseases. Substance use can also influence your ability to stick to a plan when it comes to taking PrEP to prevent HIV.

**PART 2: ACTIVITIES**

[ACTIVITY 1:  ASSESSING DRUG USE AND LEARNING ABOUT THE PARTICIPANT]

Let’s talk a little bit about your substance/drug use specifically, so I can get a better idea of how I might be able to best help you. Can you tell me a little bit about your current drug use?

*[Prompts can include: what kinds of drugs do you take? How often do you take these drugs? Under which circumstances do you take these drugs?*

How has your pattern of drug use changed over time?

 [ACTIVITY 2: DISCUSSING DRUG USE AND STI RISK]

- Can you tell me about what you know about the link between drug use and STI risk? Or, what you think the link might be?
- What about the link between drug use and sexual behavior?
- How do you think drug use and STI risk are related?

There are two main ways that drug use can influence how you can contract a disease like HIV.

- One way is through injection drug use via a contaminated needle.
- The other is a change in behavior that can result from your impaired mental state.

You may make different decisions about who you have sex, how you have sex, and what you do to protect yourself (including condoms and PrEP use) if you weren’t using drugs.

*[If the person injects drugs you can share the information in the bullet below, otherwise it is ok to skip:]*

- When a person with HIV or HCV uses a needle to inject drugs and then a person without HIV uses the same needle that has been contaminated (with blood or other bodily fluids that can transmit HIV), the person who did not have HIV originally can contract HIV when they inject the needle into their own skin.

[*Move onto these questions]*

- What is something that you enjoy about having sex while using drugs? What is something that you don’t like about it?

Your risk for STIs can increase when you are using drugs because the drugs may cause you to behave differently than you otherwise might. For example, you might forget to take your PrEP pill in good time before you are engaging in risky sex (ex. sex with a sex worker or someone else who’s HIV status you do not know, or someone with a known positive HIV test result who you don’t know is on HIV treatment), or you might be less likely to remember to use a condom during sex with a risky partner.

- Has this ever happened to you, or someone you know before? If so, what happened?
- How did you react afterward? What, if anything, do you wish you might have done differently?

[ACTIVITY 3: MOTIVATIONAL INTERVIEWING EXERCISE]

Now that we have talked a bit about drug use and STI risk, I would like to learn more about you and your goals for your life and your health.  What impact, if any, do you think your drug use might have on your life?

- I would like you to think of what some of your priorities and/or goals are for your life at the moment?
- What are some things that you would like to accomplish? When thinking about this, we would like you to focus on your health, especially STI risk and your drug use.

*[If the participant struggles, you can prompt them with the following:]*

Your goal or priority might be directly to improve your health through addressing your drug use and continuing to choose ways to protect yourself against HIV and other STIs. Or your goal might be to get healthier to improve your relationships with family or friends or your work opportunities*.*

- What are some of the patterns you may have noticed about drug use?
- Are there certain days you use drugs more?
- What about certain people?
- Or when you are in a certain mood?

*[You can also conduct the following exercise if the participant is not interested in reducing drug use. Otherwise you can move onto the homework section.]*

- What do you enjoy about taking drugs?
- What are some of the problems that arise while you are out taking drugs?
- What would make it difficult to stop taking drugs?

**PART 3: HOMEWORK/GOALS FOR NEXT SESSION**

**Appendix 1: SMART GOAL**

For your homework: I would like you to set a SMART goal for improving your health like you outlined in the activity above. This goal could be about setting reminders to take PrEP in good time on nights when you think you might take drugs, finding ways to remind yourself to bring condoms to outings when you think you might take drugs and partake in risky sex, etc.

Example for participant who wants to reduce drug use: “I am going to reduce the number of times per week that I smoke ganga, and make sure neither time is when I am planning to have sex.”

- **Specific**: I have stated clearly the number of times I take ganga now on average and compared it to the number of times I will take it in the future, and when.
- **Measurable:** I am going to reduce the number from once every day to 2 times per week and track when I take the ganga compared to when I have sex.
- **Actionable:** This is a action I can take, because I am in control of how much ganga I take and when I take it.
- **Realistic:** I am not ready to reduce the amount of ganga I take from every day to not at all right now, so this goal is more realistic than if I were to say I was going to stop all at once.
- **Timely:** I am going to begin this goal today, so that by one week from now I should have only taken ganga twice.

*[Note for the navigator: continue to use motivational interviewing]*

Now, let’s complete an example SMART goal together. Let’s pretend there is a participant who doesn’t want to reduce their drug use but wants to increase their protection against HIV while using drugs. What would their SMART goal look like? [*note, you don’t actually have to fill in the goals in the lines. This is just an exercise to help the participant.]*

- **Specific?**
- **Measurable?**
- **Actionable?**
- **Realistic?**
- **Timely?**

Now I want us to come up with a SMART goal for you and your substance use together, especially as it relates to your health concerning your risk for STIs.

[*Complete the activity in Appendix 1].*

**PART 4: SUMMARY OF MODULE**

Today we talked a bit about your drug use, including what you like and dislike about it, as well as how STI risk can increase with certain types of drug use. We talked about SMART goals and set a SMART goal for your health related to your drug use. I am excited to hear about how you felt after you set this goal, and if it was easy or hard to make progress toward it, at our next navigation session.

Do you have questions for me before we wrap up for today?

**Appendix 1: Creating a SMART goal for you and your substance use**

My SMART goal is to: ­­­­­­_____________________________________________________

**Specific:**

**Measurable:**

**Actionable?**

**Realistic?**

**Timely?**

### **MODULE H: ALCOHOL USE**

*[NOTES FOR SYSTEMS NAVIGATOR:*

*[As a reminder, our goal is to use motivational interviewing to understand if the participant is aware of the impacts their drinking might have on their sexual risk, and if they are interested in changing their drinking habits to improve their health.]*

*[There may be substantial health hazards to completely stopping alcohol use if a participant is a dependent user. If the participant drinks daily or almost daily and drinks more than 2 drinks a day they are likely to be alcohol dependent. Inform them that it can be dangerous if they quit immediately and completely. It is advisable for them to reduce alcohol consumption by 10% each day for 10 days.]*

*[Objectives for the Alcohol Use module are]:*

1. *[Provide the participant information about alcohol use.]*
2. *[Provide the participant with options for reducing alcohol use.]*
3. *[Set homework for next session.]*

**PART 1: INTRODUCTION**

In this module, we are going to focus on alcohol use. We will talk about your alcohol use, discuss the risks associated with high-risk drinking, and review strategies to help stop or cut back on drinking.

Some people take alcohol infrequently (for example, once per month), while others can take alcohol often, including every day. The amount of alcohol you take can have different impacts on your mood and/or your behavior. It is important to understand how the use of alcohol can influence your risk of contracting an STI and/or other infectious diseases. Alcohol use can also influence your ability to stick to a plan when it comes to taking PrEP to prevent HIV.

Let’s talk a little bit about your alcohol use specifically, so I can get a better idea of how I might be able to best help you.

- Can you tell me a little bit about your current alcohol use?
- In general, how often do you consume alcoholic beverages?
- In general, what types of drinks do you have?
- On average, how many drinks per day would you have?
- Describe a typical situation when you decide to drink.

How has your pattern of drinking changed over time?

- Can you tell me about what you know about the link between alcohol use and STI risk? Or, what you think the link might be?
- What about the link between alcohol use and sexual behavior? How do you think alcohol use and STI risk are related?

The main ways that alcohol use can influence how you can contract a disease like HIV is through a change in behavior that can result from your impaired mental state. You may make different decisions about who you have sex, how you have sex, and what you do to protect yourself (including condoms and PrEP use) if you weren’t using drugs.

Your risk for STIs can increase when you are taking alcohol because the alcohol may cause you to behave differently than you otherwise might. For example, you might forget to take your PrEP pill in good time before you are engaging in risky sex (ex. sex with a sex worker or someone else who’s HIV status you do not know, or someone with a known positive HIV test result who you don’t know is on HIV treatment), or you might be less likely to remember to use a condom during sex with a risky partner.

- Has this ever happened to you, or someone you know before?
- If so, what happened? How did you react afterward?
- What, if anything, do you wish you might have done differently?

**PART 2: ACTIVITIES**

[ACTIVITY A: LOW-RISK DRINKING AND EFFECTS OF HIGH-RISK DRINKING]

Thank you for sharing that information with me. I now want to discuss information with you about low-risk drinking. For people who are at risk of acquiring HIV, it may be helpful to reduce alcohol use or to quit drinking altogether. The reasons that this is particularly important for you is that drinking can make it harder to take PrEP as recommended. Alcohol use can also cause problems in your relationships and reduce the amount of support that you have from your family and friends.

However, if you do like to take alcohol, it’s safe to take PrEP before, after, and on days when you do so. It is important to take extra steps to make sure you take PrEP consistently every day when you are out “partying” so that you do not forget your PrEP at home or forget to take it.

[ACTIVITY B: USE “WHAT’S A STANDARD DRINK” ]

**Refer to Appendix 1: Standard Drink Chart**

It is important for you to determine how much alcohol is in each beverage you usually drink, so you can reduce the amount you drink.

Most bottles and cans of beer have about the same amount of alcohol as a glass of wine or one shot of distilled spirits. When you think about how much you drink, be sure to count standard drinks.

[*Review Standard Drink Chart in Appendix 1 to ensure the participant understands what constitutes a standard drink].*

Having 3 or more drinks on one occasion creates risks of “accidents” involving injuries, problems in relationships and at work, and medical problems such as hangovers, sleeplessness, and stomach problems. Drinking more than 2 drinks per day over extended periods may cause cancer, liver disease, depression, and dependence on alcohol (alcoholism). If you have been drinking above these limits, you risk causing harm to yourself and others.

[ACTIVITY C: DISCUSS THE “EFFECTS OF HIGH-RISK DRINKING” IMAGE WITH PARTICIPANT (Appendix 2) ]

[*If the participant says that they drink excessively – for a male, “excessive” is typically more than two drinks a day or more than 14 drinks in a week]*

**Refer to Appendix 2: Effects of High-Risk Drinking Chart**

- What do you know about excessive drinking?
- Describe for me what a typical drinking is like for you. Are there other people around? Do they drink the same amount as you? More or less?
- What do you like about drinking a lot? What are some of the things you do not like about it?
- Do you know about any of the health effects of high-risk drinking? If so, what have you heard?

[*Go to appendix 2 and review the risks of high-risk drinking chart with participant].*

- Had you heard any of this information before?
- What information was new for you? What was surprising for you to learn?
- How does hearing these effects of high-risk drinking make you feel?

Fortunately, most people can stop or reduce their drinking if they decide to do so and work hard at changing their drinking habits.

[ACTIVITY D: ASK THE PARTICIPANT HOW TO PROCEED] (****suggested)***

**Are you interested in controlling or reducing your alcohol use?**

- *[If yes, continue to Activity E]*
- *[If no, conduct MI exercise below.]*

*[MI Exercise: conduct if participant is not interested in reducing alcohol consumption]*

Now that we have talked a bit about alcohol use and STI risk, I would like to learn more about you and your goals for your life and your health.

- What impact, if any, do you think your alcohol use might have on your life?

I would like you to think of some of your priorities and/or goals are for your life at the moment.

- What are some things that you would like to accomplish? When thinking about this, we would like you to focus on your health, especially STI risk and your alcohol use.

*[If the participant struggles, you can prompt them with the following]:*

Your goal or priority might be directly to improve your health through addressing your alcohol use and continuing to choose ways to protect yourself against HIV and other STIs. Or your goal might be to get healthier to improve your relationships with family or friends or your work opportunities.

- What are some of the patterns you may have noticed about your alcohol use? Are there certain days you use alcohol more? What about when you are around certain people? Or when you are in a certain mood?
- What do you enjoy about drinking?
- What are some of the problems that arise while you are out drinking?
- What would make it difficult to stop drinking?

*[Assess with participant if they are interested in doing anything to reduce drinking or addressing some of the barriers to changing their alcohol use]*

[ACTIVITY E: STOPPING OR CUTTING BACK]

When people successfully change their habits they usually follow a simple plan. If possible, try to get somebody to help you. Perhaps a friend or a relative, a health worker, member of your religious community, or someone you’ve disclosed your participation in the trial or PrEP use to would be willing and able to help you work out a plan and stick to it. The reason for getting somebody else to help is simply that two heads are better than one. Also, they will be able to provide some support. Of course, many people change their habits without help from others. If you are unable to get somebody else to help, then work out a plan by yourself.

Some people on PrEP should also stop drinking. This is a priority if:

- You drink daily and it would be difficult to just drink a small amount.
- You have tried to cut down before but have not been successful, or
- You suffer from morning shakes during a heavy drinking period, or
- You have high blood pressure, you are pregnant, you have liver disease, or
- You are taking medicine that reacts with alcohol.

If you are unwilling or unable to stop drinking, we have discussed some strategies to reduce your alcohol use. Based upon recent research on the effects of alcohol, here is a list of benefits that you can reasonably expect if you cut down on your drinking. Let’s read through them and choose three that seem to be the best reasons to you.

Choose the ones that make you want to cut down on your drinking.

If I drink within low-risk limits:

- I will live longer--probably between five and ten years.
- I will sleep better.
- I will save a lot of money.
- My relationships will improve.
- I will be less likely to get into trouble with the police.
- The possibility that I will die of liver disease will be dramatically reduced (12 times less likely).
- It will be less likely that I will die in a car accident (3 times less likely).

ACTIVITY F: REFUSAL SKILLS

Even if you are committed to changing your drinking, "social pressure" to drink from friends or others can make it hard to cut back or quit.

The first step is to become aware of the two different types of social pressure to drink alcohol—direct and indirect.

Direct social pressure: is when someone offers you a drink or an opportunity to drink.

Indirect social pressure: is when you feel tempted to drink just by being around others who are drinking—even if no one offers you a drink.

Take a moment to think about situations where you feel direct or indirect pressure to drink or to drink too much. [PAUSE]

Knowing what type of situations you may face is the first step to developing strategies to say “no”. For some situations, your best strategy may be avoiding them altogether (which we can discuss below). If you feel guilty about avoiding an event or turning down an invitation, remind yourself that you are not necessarily talking about "forever." When you have confidence in your refusal skills, you may decide to ease gradually into situations you now choose to avoid. In the meantime, you can stay connected with friends or family by suggesting alternate activities that don't involve drinking.

**Know your "no"**

When you know alcohol will be present, it's important to have some resistance strategies lined up in advance. If you expect to be offered a drink, you'll need to be ready to deliver a convincing "no thanks." Your goal is to be clear and firm, yet friendly and respectful. Avoid long explanations and vague excuses, as they tend to prolong the discussion and provide more of an opportunity to give in. Here are some other points to keep in mind:

- Don't hesitate, as that will give you the chance to think of reasons to go along
- Look directly at the person and make eye contact
- Keep your response short, clear, and simple

The person offering you a drink may not know you are trying to cut down or stop, and his or her level of insistence may vary. It's a good idea to plan a series of responses in case the person persists, from a simple refusal to a more assertive reply. Consider a sequence like this:

- No, thank you.
- No, thanks, I don't want to.
- You know, I'm (cutting back/not drinking) now (to get healthier/to take care of myself/because my doctor said to). I'd really appreciate it if you'd help me out.

You can also try the "broken record" strategy. Each time the person makes a statement, you can simply repeat the same short, clear response.

**Script and practice your "no"**

Many people are surprised at how hard it can be to say no the first few times. You can build confidence by scripting and practicing your lines. First, tell me of a situation where a person may be offering you a drink.  Now, let’s think of how you’ll respond

*[ROLE-PLAYING: It may be helpful to do role playing here. Pretend to be the person offering the drink and have the participant practice out loud their refusal strategy.  Let the participant know that practice will help them gain confidence and feel better about refusing.]*

ACTIVITY G: ALTERNATIVES TO DRINKING

Your desire to drink heavily probably changes according to your moods, the people you are with, and whether or not alcohol is easily available. Think about the last time you drank too much and try to work out what things contributed to your drinking. What situations will make you want to drink heavily in the future?

Some examples may include:

- Situations in which other people are drinking and I am expected to drink.
- Feeling bored and depressed, especially on weekends.
- After a family argument.
- When drinking with my friends.
- When feeling lonely at home.

Many people drink because they are bored. If boredom contributes to your drinking beyond low-risk limits, let’s think of as many activities as we can that might hold your interest and then select 2 of them to try.

- What types of things have you enjoyed learning in the past? (e.g., sports, crafts, languages)
- What types of trips have you enjoyed in the past? (e.g., to the ocean, to the mountains, to the country)
- What types of things do you think you could enjoy if you had no worries about failing? (e.g., painting, dancing)
- What have you enjoyed doing alone? (e.g., long walks, playing a musical instrument, sewing)
- What have you enjoyed doing with others? (e.g., talking on the telephone, playing a game, having tea)
- What have you enjoyed doing that costs no money? (e.g., playing with your children)
- What have you enjoyed doing that costs very little (e.g., going to a park)
- What activities have you enjoyed at different times? (e.g., in the morning, on your day off work, in the spring, in autumn)
- What 2 activities should you try the next time you think about drinking?

**PART 3: HOMEWORK FOR NEXT SESSION**

- How are some ways you think you can reduce your alcohol use? What do you think is the best way you can reduce your alcohol from the strategies we have discussed? *[Probe: suggest some strategies just discussed]*
- What challenges do you think you will face in making these changes? How will you address these challenges? *[Give suggestions]*
- Can you create a plan to stop/cut back your drinking, based on the goals you identified in our discussion?
- Can you identify 2 strategies to use the next time you feel the desire to drink more than you desire/ or are faced with being offered a drink at all (if you hope to stop completely)?

**PART 4: SUMMARY OF MODULE**

The point of this activity was to discuss the risks associated with high-risk drinking and review strategies to help stop or cut back on drinking.

A few key points to remember:

- Remember that every time you are tempted to drink too much and are able to resist, you are breaking your habit.
- Whenever you feel very uncomfortable, distressed or miserable, keep telling yourself that it will pass. If you crave a drink, pretend that the craving is like a sore throat that you have to put up with until it goes away.
- If you have a helper, tell that person honestly how much you had to drink each day and when you have been successful or have drunk too much.

Finally, it is likely that you will have some bad days on which you drink too much. When that happens, DON'T GIVE IN. Remember that people who HAVE learned to drink at low-risk levels had many bad days before they were finally successful. It will get easier in time.

Is there any information in this module that was not clear? Do you have any additional questions?

**Appendix 1: Standard Drink Chart**

1 standard drink =

| 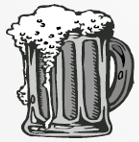 | 1 can of ordinary beer (e.g. 330 ml at 5%) |
| --- | --- |
|  | or |
| 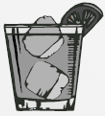 | A single shot of spirits (whiskey, gin, vodka, etc.) (e.g. 40 ml at 40%) |
|  | or |
| 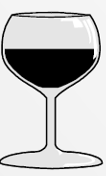 | A glass of wine or small glass of sherry  (e.g. 140 ml at 12% or 90 ml at 18%) |
|  | or |
| 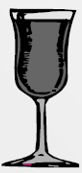 | A small glass of liqueur or aperitif  (e.g. 70 ml at 25%) |
|  | or |
| 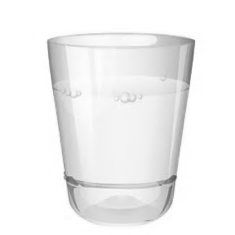 | A cup of traditional or home brew  (e.g. 85 ml, >20% alcohol) |

**Appendix 2: Effects of High-Risk Drinking Chart**


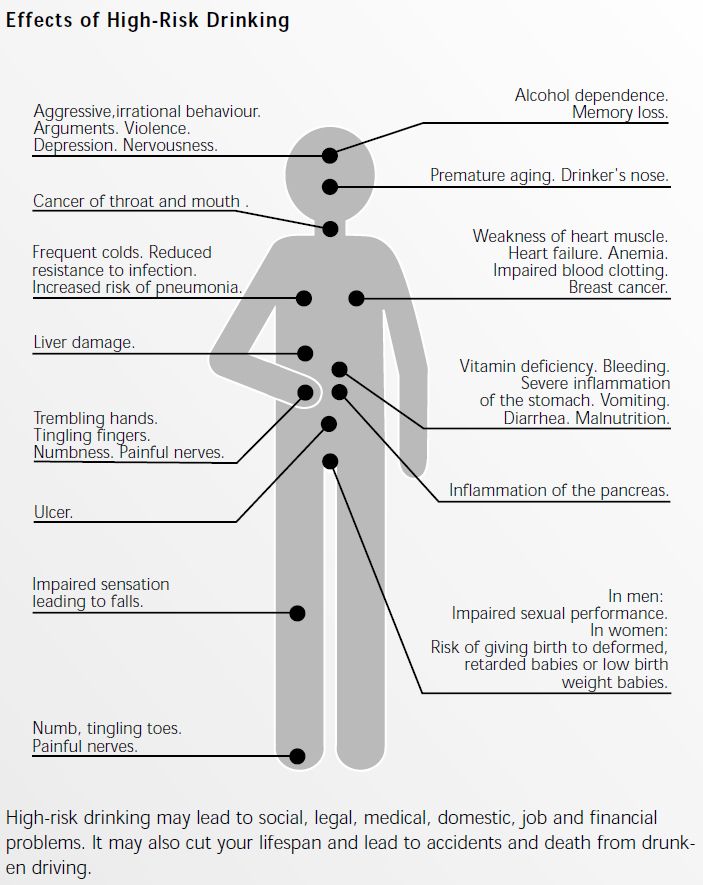


### **MODULE I: PSYCHOSOCIAL DISTRESS AND SUPPORT**

*[NOTES FOR SYSTEMS NAVIGATOR]*

*The goals and objectives for the Psychosocial Distress and Support module are:*

- *[Define, identify and cope with stressors in the participant’s life.]*
- *[Describe social support to mitigate stress and do a related activity.]*
- *[Set homework for next session.]*

**PART 1: INTRODUCTION**

We’ve been talking a lot these last few sessions about adherence and also disclosure of PrEP use. Sharing something personal about yourself with another person can be stressful. For the rest of the session today, we will talk about stress and emotions. We will also talk about ways to cope with the stressors of life.

**PART 2: ACTIVITIES**

[ACTIVITY 1: WHAT STRESSES YOU]

**Refer to Appendix 1: What stresses you?**

What is stress?

*[allow participant time to reply]*

Stress is the EMOTIONAL and PHYSICAL reactions you experience because of changes and demands in your life. It is the feeling you get and the way your body reacts to these changes. Stress can come from both POSITIVE and NEGATIVE situations. For example, we associate stress with events such as being sick, or worrying about your grades or your finances. But stress can also come from positive events, such as weddings, celebrating holidays, having a baby or starting a new relationship. Stressors can also be MIXED – both positive and negative. A woman can be excited about having a new boyfriend, but she may be stressed about whether he is the right man for her. I would like you to take a few minutes to think of some of the things that cause you to feel stressed.

*[allow participant time to think]*

Now I would like to talk through some of the stressors in your life with you. We can talk about 1, or multiple.

[*Complete appendix 1 with the participant]*

ACTIVITY 2: COPING BY ADDRESSING OUR STRESSORS]

What are some ways that you currently cope with these stressors in your life?

[*allow participant time to reply]*

Is it ok if I share with you a strategy that has worked for some people in the clinic, dealing with their stress? It is called the “5 steps method”.

**The 5 steps method:**

1. Define the problem
2. Think about solutions to the problem
3. Choose the best option and try it
4. Reflect on what happened: is your problem solved? Do you need to try a new solution?
5. Try again – if the result is not what you hoped for, you can select another option and try again to solve it.

Let’s go back to your list of stressors. Do you want to select one and we can apply these steps to the stressor together?

*[Allow time for participant to apply steps to one of his stressors on his own, and then you can complete it together if he needs help.]*

ACTIVITY 3: COPING WITH STRESS BY RELAXING YOUR BODY AND EMOTIONS

Now we are going to focus on different ways to reduce stress when we notice it. What are some ways you know of to reduce stress?

*[allow participant time to reply]*

Is it ok with you if I share some examples of strategies to relax your body and your emotions that have worked for other patients in our clinic?

**Ways to relax your body:**

- Deep Breathing: While sitting, lying down or standing, close your eyes and breathe in slowly. Let your breath out for a count of 4 seconds. Take 10 of these super-relaxers any time you feel tense.
- Stretching: Practice simple stretches such as the neck stretch. Stretch your neck by gently rolling your head in a half circle, starting at one side, then dropping your chin to your chest, then to the other side. Be careful not to roll your head back.
- Exercise: All kinds of physical activity – walking, running, playing sports, dancing – help to reduce stress.
- Take a Break: Ask family members to allow you at least 30 minutes of time alone.
- Eat Well: Reduce caffeine (in coffee, tea, soda and chocolate) and alcohol intake. Take steps to eat healthy.

**Ways to relax your emotions**

- Talk: Take time to talk with a friend, partner or child. Express feelings you might have been holding in.
- Laugh: Go see a funny movie, watch a funny video, or spend time with a friend who makes you laugh.
- Cry: Crying can be as good a release as laughing. If you haven‘t cried in a long time, try listening to sad music or watching a sad movie.
- Read: A good book is a great escape.
- Do something you love: When you enjoy yourself, whether you are dancing, going to the park, or seeing friends, you relax your emotions.
- Do something you are good at: When you build mastery in a task, you develop confidence, which can in turn positively affect your outlook and/or put you in a good mood.
- Write or draw: Another way to reduce stress is to write things down (like a to-do list) or to draw pictures about how you feel. When you are stressed or angry and have no one to talk to or are not comfortable with talking, try writing or drawing your feelings.

[ACTIVITY 5: COPING WITH STRESS USING SOCIAL SUPPORT]

**Refer to Appendix 2: Who is on your mini-bus?**

If you could fill a min-bus with the people in your life who are most important, who would be on that bus?

*[Navigator writes the name of each person (represented by the circle) with a person/group that is most important in their lives such as family, friends, teachers, partners, etc.*

*Ask participants to include only people (i.e., no pets, inanimate objects, etc.)]*

*[After you have listed out the names of all the people on the mini-bus, ask the participant the following questions.]*

- Who are the people/groups on your bus?
- Why are they important to you?
- Are there some that have more influence than others?
- Are these people/groups you can count on when you are in trouble or in need?
- Do they help you make good decisions? Always? Most of the time? Sometimes? Never? Do you feel good about the decisions they help you make?

Thank you for sharing with me who are the most important people to you. We will continue to refer back to these groups in future sessions. In the meantime, I hope that you are able to reach out to these people when you need them. If you are struggling with asking them for help, we can practice role-playing, or using another one of our strategies to reach out to them.

**PART 3: HOMEWORK/GOALS FOR NEXT SESSION**

[*Select one that seems the most appropriate for your participant].*

- Identify times when you have felt both positive and negative (or mixed) stressors at the same time. How did you cope?
- Other than how the people on your bus may help or hinder you in your decision-making, think about reasons why they are important to you and, thus, were able to grab a seat on your bus.
- If you are in need of some support and the people on your mini-bus don’t yet know that, perhaps we could set of goal of you reaching out to one of them?

**PART 4: SUMMARY OF MODULE**

Today we talked about stress, coping with stress, and social support. Do you have any questions about anything in this module for now?

**Appendix 1: What stresses you?**

*[Note: the participant does not need to provide 3 stressors. They can provide 1, 4, etc. This is just a template to help them talk about what is stressing them.]*

|  | **What is the stressor?** | **What do you feel? Why do you think it stresses you?** |
| --- | --- | --- |
| **Stressor 1** |  |  |
| **Stressor 2** |  |  |
| **Stressor 3** |  |  |

**Appendix 2: Who is on your mini bus?**


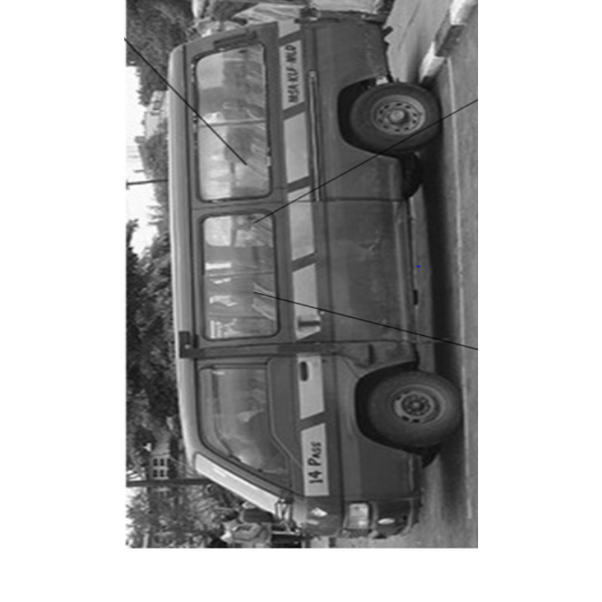


**MODULE J: WRAP-UP**

*[NOTES FOR SYSTEMS NAVIGATOR]*

*[The goal for the wrap-up module is to provide the participant a reminder of the journey you have been on together, and to set them up for success now that your navigation sessions are ending. An important part of this exercise is to remind them that they have the tools to be successful in reducing their STI risk and adhering to PrEP even without you as they move forward. It will be important to review the participant folder in detail before the session.]*

**PART 1: INTRODUCTION AND RECAP OF ACTIVITIES**

Today is our last day working together with me as your Systems Navigator. I have very much enjoyed our time together and would like to take a look back on the long journey we have taken together.

[*Take this time to go back through the participant folder/session notes and remind them of where they started and how far they have come. Reference some of the activities you have completed together, and take note of important things that were shared with you over the course of your time working with the participant].*

**PART 2: WRAP-UP AND ENCOURAGEMENT**

[*Feel free to tailor the closing speech to whatever feels most personal and appropriate for the participant you are working with. These are just sample prompts to help you think through what you might want to incorporate.]*

- As you can see, we have done a lot together during our time here in the Njira study. I have really enjoyed getting to work with you as your navigator and have been so excited to see the journey you have been on.
- Thank you for trusting me with your sensitive information. I know at times we talked about very personal matters, and I really appreciated you being so willing to share this information with me.
- We have worked together to build many skills and practice several strategies to improve your sexual health and adherence to PrEP. Some of these strategies included Safe TALK, role playing and listing out the pros and cons to a decision. We also practices keeping a calendar for your PrEP doses and refill appointments, strategies to manage psychosocial distress, reduce your risk of STI acquisition during substance use, negotiating condom use and inquiring about circumcision. You should be confident in using these skills on your own, even though our time together is ending.

Before we end today, I want to provide you with some time to ask me any remaining questions that you might have. [*Leave time for the participant to respond]*.

As a reminder, you can always come back to the clinic to ask questions about healthy sexual practices, STI risk, PrEP and any other health issues you might be having. The clinic staff is here to help you, and you are equipped with the skills to ask questions about all of the topics we have covered.

Thank you very much for working with me over the past several months.

# **Chapter 9: DOCUMENTATION**

### **Overview**

There are four forms of documentation for the systems navigation intervention:

1. Session checklist: to be completed by navigators, the checklist contains all the activities to be completed by a Navigator during a navigation session.
2. Session notes: a form for navigators to complete during the session and to take notes specific to the participant they are counseling. These are kept in the participant folder for the navigator to refer again to in advance of their next session.
3. Systems Navigator Contact Case Report Form: to be completed by the navigators at the end of any contact with a participant. Tracks information such as time spent engaging with the participant and the nature of the interaction.
4. Weekly Intervention Debrief Form: This is to be physically filled out by the study coordinator, using information provided by the navigators during a weekly debrief meeting. Navigators should reference their personal journals to contribute to this discussion as well as their memory of activities from the previous week.

There are three additional tools to assist navigators in tracking their activities and to fulfill protocol objectives:

1. Personal journal: each navigator is provided an independent journal to capture reflections about their experiences overall, trends, lessons learned, etc. This is not meant to be a place to write down specific things about any one participant, but rather to help facilitate quality improvement throughout the course of the intervention. Navigators use the reflections in their notebooks to contribute to the weekly debrief sessions. No confidential or identifying information should be captured in journals.
2. Calendar for PrEP visits and reminders: navigators receive a calendar to write down the PID for participants who have upcoming PrEP visits as well as the dates when they should be issuing PrEP visit reminders by phone call or text message (if the participant has opted into this).
3. Laboratory requisition form: the final piece of documentation that navigators interact with is the requisition form for the point of care STI tests to be run on the urine specimen collected by the navigator.

*Examples of the first four documents are provided in the subsequent pages.*

### **Navigation Session Checklist**

**PID** 
**Date:**

**Navigator ID:**

| **Check if done** | **Task** |
| --- | --- |
| *Tasks completed during the participant visit/encounter* | |
|  | Collect urine sample (not apply for enrollment visit) |
|  | Complete lab requisition form/deliver specimen to lab |
|  | Confirm the locator information is still accurate |
|  | Update tracing preferences as needed |
|  | Assess ongoing HIV risk |
|  | Deliver navigation session based on participant needs |
|  | Confirm date of next PrEP visit *after* patient has met with PrEP nurse |
|  | Confirm preference for PrEP visit reminder |
|  | STI result delivered |
|  | If positive STI result, referred to care (write N/A if the STI result was negative) |
|  | If participant also within target window for study visit, can refer to CVDH (write N/A if not within target window) |
| *Tasks completed after the participant visit/encounter* | |
|  | Review session notes (confirm you’ve documented everything you need for the CRF) |
|  | Complete the relevant visit CRF sections |
|  | Document next PrEP visit (and preference for reminder) in **PrEP visit tracking log** |
|  | Use diary to reflect on content, quality, and concerns raised during session |

**STI/HIV Risk Questions**

1. Is the participant currently having sex? If so, with about how many partners?
2. How often does the participant use condoms while having sex?
3. Does the participant have any partners with a known HIV status?
4. Does the participant have any partners for which the HIV status isn’t known?

### **Navigation Session Notes**

**Next PrEP visit date:**

**Wants reminder?**

Topics to note down:

- HIV Risk/Risk Perception
- PrEP modality
- Motivations for taking/ adhering to PrEP
- Barriers to PrEP
- Notable events in participant’s life/anything else important to note for future sessions:
- Goal set for the next session

|  |
| --- |
|  |
|  |
|  |
|  |
|  |
|  |
|  |
|  |
|  |
|  |
|  |
|  |
|  |
|  |
|  |
|  |

|  |
| --- |
|  |
|  |
|  |
|  |
|  |
|  |
|  |
|  |
|  |
|  |
|  |
|  |
|  |
|  |
|  |
|  |
|  |
|  |
|  |
|  |
|  |

### **Systems Navigator Contact Case Report Form**

**Participant ID :**

**Navigator ID :**

**Date of contact** (DD/MM/YYYY):

**Section A: Purpose of contact**

1. What was the purpose of this contact?
   1. Navigation session
      1. If yes, complete section B (Navigation session)
   2. Tracing for missed PrEP visit
      1. If yes, complete section C (Tracing for missed PrEP visit)
   3. PrEP visit reminder/interim engagement
      1. If yes, complete section D (PrEP visit reminder/interim engagement)
   4. Notification of STI test result
      1. If yes, complete section E (STI Test Result notification)
   5. Participant initiated contact
      1. If yes, complete section F (Participant initiated contact)

**Section B: Navigation session**

1. Is the participant willing to be traced in-person?
   1. Yes
   2. No
2. Visit length
   1. 0-14 minutes
   2. 15-29 minutes
   3. 30-44 minutes
   4. 45-60 minutes
   5. More than 1 hour
3. Did the participant report any HIV risk behaviors (condomless sex with someone whose HIV status they do not know or is known positive but with unknown ART use) in the past 3 months?
   1. Yes
   2. No
   3. Did not disclose
4. Modules that were conducted today (select all that apply)
   1. Module A: Introduction and study overview
   2. Module B: PrEP Overview and Adherence
   3. Module C: Barriers to PrEP Adherence and Problem Solving
   4. Module D: PrEP Disclosure
   5. Module E: Sexual Health & STI Prevention
   6. Module F: Circumcision
   7. Module H: Substance use (other than alcohol)
   8. Module I: Alcohol use
   9. Module J: Psychosocial support and distress
   10. Other/non-module: spe’cify general content (free response)
5. Was a urine specimen collected today?
   1. Yes ->Q7
   2. No -> Q6
6. If not, why was no urine specimen collected? *(selection one then END FORM)*
   1. Insufficient time during navigation session
   2. Participant declined (for reason other than insufficient time)
   3. STI test completed within last 14 days
   4. Other

1. N. Gonorrhea – urine:
   1. Not detected/Negative
   2. Positive/Reactive
   3. Invalid
   4. Indeterminate
2. C. trachomatis – urine:
   1. Not detected/Negative
   2. Positive/Reactive
   3. Invalid
   4. Indeterminate
3. Were the STI test results returned to the participant today?
   1. Yes -> Q11
   2. No -> Q10
4. Why were the results not returned to the participant?
   1. The STI results were not available today
   2. The STI results were available today, but the participant left before they could be returned
   3. Other (specify)
5. Was the participant referred for treatment?
   1. Yes
   2. No
   3. Not applicable
6. Did the participant agree to receive treatment?
   1. Yes
   2. No
   3. Not applicable
7. Did you discuss any systems navigation content after delivering the STI test results?
   1. Yes
      1. What did you discuss?
         1. Barriers to PrEP use
         2. Strategies for adherence
         3. STI and HIV risk
         4. Other (specify)
   2. No
   3. Not applicable

[END FORM]

**Section C: Tracing for missed PrEP Visit**

1. How did you trace the participant?
   1. On the phone (select one from below, then skip to Question 4)
      1. Phone call
      2. SMS (including WhatsApp)
   2. In person (select one from below, then proceed to Question 2)
      1. At home
      2. At work
      3. At the market
      4. Other (specify)
2. How many minutes did you spend *in transport/attempting to find*?
   1. 1-15
   2. 16-30
   3. 31-45 minutes
   4. 46-60 minutes
   5. More than 60 minutes
3. What did you use for transport?
   1. Project vehicle
   2. Motorbike
4. How many minutes did you spend *interacting with* the participant?
   1. 0 (patient not found/no contact made) à **if no contact, end of form**
   2. 1-5 (could include time spent exchanging texts/on phone)
   3. 6-15
   4. 16-30
   5. 31-45
   6. 46-60 minutes
   7. More than 60 minutes
5. Did the participant provide a reason for missing their PrEP visit? (check all that apply)
   1. No money for transport
   2. Forgot
   3. Busy with work
   4. Has decided to discontinue PrEP (à skip to Question 7)
   5. Other (specify)
6. Is the participant still taking PrEP?
   1. Yes
   2. No
   3. Did not say
7. Did the participant agree to report to the clinic for resumption/continuation of PrEP?
8. Yes (à skip to Question 9)
9. No
10. (Only complete if tracing in person) Did navigator offer the PrEP restart kit?
11. Yes
    - 1. (if YES) Did the participant accept the HIV self-test kit?
         1. Yes
         2. No
         3. Self-test kit not available
      2. (if YES) Did the participant accept the PrEP pills?
         1. Yes
         2. No
         3. PrEP pills not available
12. No
    - 1. (If NO) why did you not offer the restart kit?
         1. Location not discreet enough to offer the restart kit.
         2. Navigator forgot the kit and/or forgot to offer it.
         3. Other (specify)
13. Did you discuss any systems navigation content?
    1. Yes
       1. (If YES) What did you discuss? (select all)
          1. Barriers to PrEP use
          2. Strategies for adherence
          3. STI and HIV risk
          4. Other (specify)
    2. No

[END FORM]

**Section D: PrEP visit reminder/interim engagement**

1. How did you trace the participant?
2. On the phone (select one from below, then skip to Question 4)
   - 1. Phone call
     2. SMS (including WhatsApp)
3. In person (select one from below, then proceed to Question 2)
   - 1. At home
     2. At work
     3. At the market
     4. Other (specify)
4. How many minutes did you spend *in transport/attempting to find the participant*?
5. 1-15
6. 16-30
   1. 31-45 minutes
   2. 46-60 minutes
   3. More than 60 minutes
7. What did you use for transport?
8. Project vehicle
9. Motorbike
10. How many minutes did you spend *interacting with* the participant?
11. 0 (patient not found/no contact made) à **if no contact, end of form**
12. 1-5 (could include time spent exchanging texts/on phone)
13. 6-15
14. 16-30
15. 31-45
16. 46-60 minutes
17. More than 60 minutes
18. Did you discuss any systems navigation content?
    1. Yes
       1. (If YES) What did you discuss? (select all)
          1. Barriers to PrEP use
          2. Strategies for adherence
          3. STI and HIV risk
          4. Other (specify)
    2. No

[END FORM]

**Section E. STI test result notification** *(to be filled in only if results not returned on same day as navigation session)*

1. How did you attempt to contact the participant to notify them of their STI test results?
2. On the phone (-> select one from below, then skip to Question 4)
   - 1. Phone call
     2. SMS (including WhatsApp)
3. In person (-> select one from below, then proceed to Question 2)
   - 1. At home
     2. At work
     3. At the market
     4. Other (specify)
4. How many minutes did you spend *in transport/ attempting to find the participant*?
   1. 1-15
   2. 16-30
   3. 31-45 minutes
   4. 46-60 minutes
   5. More than 60 minutes
5. What did you use for transport?
   1. Project vehicle
   2. Motorbike
6. How many minutes did you spend *interacting with* the participant?
   1. 0 (patient not found/no contact made) à **if no contact, end of form**
   2. 1-5 (could include time spent exchanging texts/on phone)
   3. 6-15
   4. 16-30
   5. 31-45
   6. 46-60 minutes
   7. More than 60 minutes
7. Was the participant referred for treatment?
   1. Yes
   2. No
   3. Not applicable
8. Did the participant agree to receive treatment?
   1. Yes
   2. No
   3. Not applicable
9. Did you discuss any systems navigation content after delivering the test results?
   1. Yes
      1. What did you discuss?
         1. Barriers to PrEP use
         2. Strategies for adherence
         3. STI and HIV risk
         4. Other (specify)
   2. No

[END FORM]

**Section F: Participant initiated contact**

1. How did the participant contact you?
   1. Phone call
   2. SMS (including WhatsApp)
   3. At the clinic
   4. Other (specify)

1. How many minutes did you spend *interacting with* the participant?
   1. Less than 1 minute
   2. 1-5 (could include time spent exchanging texts/on phone)
   3. 6-15
   4. 16-30
   5. 31-45
   6. 46-60 minutes
   7. More than 60 minutes

1. What was the purpose of their contact?
   1. Rescheduling PrEP visit
   2. Following up for STI test results
   3. General sexual health counselling
   4. Other (specify)

1. Did you discuss any systems navigation content?
2. Yes
   - 1. (If YES) What did you discuss? (select all)
        1. Barriers to PrEP use
        2. Strategies for adherence
        3. STI and HIV risk
        4. Other (specify)
3. No

**[END FORM]**

### **Weekly Intervention Debrief Form**

**Date:**

**Attendees:**

**Instructions**: *Navigators will use notes from their notebooks to answer the questions below. Matthews will help to facilitate but can also provide commentary based upon his assessments at the clinic.*

1. How are participants in the session? Do they seem comfortable and free to respond? Are some of the participants reserved?
2. In the prior week through your interactions with participants, supporters, and the health system, what additional information did you learn about barriers and facilitators to PrEP and drug treatment?
3. Are their important topics that are not covered in the sessions?
4. Which intervention techniques (like motivational interviewing) or modules (like PrEP 101) seem to be particularly helpful?
5. What additional text or topics could be helpful?
6. What are some challenges that you have faced?
