# Supplementary material for: Improving HIV Prevention Among Heterosexual Men Seeking Sexually Transmitted Infection Services in Malawi: Protocol for a Type I Effectiveness-Implementation Hybrid Randomized Controlled Trial of Systems Navigator–Delivered Integrated Prevention Package (HPTN 112-NJIRA Study)
Source: JMIR Res Protoc. 2025 Jun 18;14:e72981. doi: 10.2196/72981 (PMC12223453; doi:10.2196/72981)
Supplement: Multimedia Appendix 2 [file resprot_v14i1e72981_app2.docx]

**Appendix 1: In-depth Interview Guide for PrEP Patients**

*[Read or paraphrase to participant]*

Thank you for agreeing to talk with me today. My name is [NAME]. I am part of a research team seeking to improve HIV prevention among men in Malawi, specifically through use of pre-exposure prophylaxis supported through a navigator. As you may know, pre-exposure prophylaxis (or PrEP) can help reduce the risk of an HIV-uninfected person becoming infected with HIV, if the PrEP medication is taken as prescribed.

We want your honest opinions – there are no right or wrong answers, what we want to know is what you think and believe. If there are any questions that make you feel uncomfortable, feel free to tell me and we can skip or come back to those questions.

Anything that you say to me will be kept confidential. Our discussion will be audio recorded and then transcribed. When it is written out, all information that could identify you to someone reading the words (anything said with names or locations or that kind of thing) are completely removed. You may opt not to have the interview recorded if you wish.

The material from our interview is saved with your identification number but not your name. The research team members reviewing this text will not link who you are to that ID and will not tell anyone on site about your specific feedback. There are other people doing interviews as well, and we will put all those documents together and look at main topics people talked about. We will not identify or share anything you say as a comment linked to your name or ID number.

Do you have any questions before we begin?

1. Can you please begin by telling me about when you first heard about this study and what made you decide to join it?

1. Can you tell me about when you first heard about PrEP?

1. Can you tell me more about the kind of PrEP you are on now (or the kind you have taken most recently)?
2. In what ways has your PrEP use been impacted by these changes?

1. When you were coming for STI services at this clinic and starting PrEP had you ever heard of different kinds of PrEP?

1. What questions did you have for the PrEP nurse when you were leaving the clinic with your PrEP?

1. Can you tell me about how it has been to receive PrEP care at the Bwaila STI clinic?

1. Have you ever stopped taking PrEP, or considered stopping PrEP use? Why or why not?

1. What helps you remember to take your PrEP and refill your PrEP prescriptions (or come for injections at the clinic if you are taking CAB-LA)?

1. Have you told anyone that you are taking PrEP?

1. Before enrolling into this study, what did you think of people who use PrEP?
2. Before you started this study, did you think PrEP was more for men or women?

1. What do you think might be some challenges with taking PrEP that are different for men than for women? By “taking PrEP” I mean both taking the medication (a pill or an injection) but also coming back into the clinic for the regular check-ups/check-ins.

1. Can you describe what your ideal environment would be to receive PrEP?

1. Some persons believe that “*Real men don’t use condoms”, or that “Real men have many sexual partners.”* What have you heard about such things? Has your thinking about this changed at all during your time in the study?

1. What are some of the things that put you at risk of acquiring HIV?

1. You initially came into the clinic because you had symptoms of an STI. Does having an STI, or symptoms of an STI, change your perceived risk for HIV in any way? Why or why not?

1. What if you were told that you had an STI based on a urine or blood test but were without any symptoms?

1. Do you feel like your risk of contracting an STI is different now than when you first started on the study? If so, why and in what way?

1. Do you feel like your sexual activities have changed at all since starting PrEP?

1. As you may recall, this study is designed such that people are randomly assigned to either have a navigator, or to remain in standard PrEP care, without a navigator. When you learned that you were assigned to the arm that would not work with a navigator, how did you feel?
2. Is there anything else you would like to share with me about your experience in the study, or in general?

1. Do you have any questions for me?

*[END OF INTERVIEW FOR CONTROL PARTICPANT]*

1. As you may recall, this study is designed such that people are randomly assigned to either have a navigator, or to remain in standard PrEP care, without a navigator. When you learned that you were assigned to the arm that would work with a navigator, how did you feel?

1. Navigators were selected based on their experience working in sexual health and HIV prevention. They were also chosen to be male, and approximately the same age as many men initiating PrEP at the Bwaila clinic. What other characteristics do you think are important in a navigator?

1. What were your expectations for working with the navigator?

1. Where did you talk with your navigator during your PrEP follow-up visits?

1. How did you feel when interacting with your navigator?

1. What did you talk about with your navigator?
2. What were some of the challenges of working with your navigator?

1. How often did you meet with the same navigator? How important was it to you that your navigator was the same person throughout your sessions?

1. The navigators were trained to help you develop your own goals for remaining free from HIV. In what ways do you feel like you were empowered to set and reflect on those goals?

1. Can you reflect on one of the more memorable or meaningful interactions you have had with a navigator? Perhaps something you learned or that you think influenced your health behavior when it comes to sexual health?

1. In general, would you say your mental health is poor, fair, good, very good, or excellent?

1. Can you tell me about some situations where you may drink alcohol or use weed or any other drugs when having sex?

1. How did your navigator influence your decision to stop or stay on PrEP? Did your engagement with a navigator have any influence on how you interacted with the PrEP nurse (for example, what questions you asked them)?

1. What did you think about having STI testing done during your PrEP care?

1. Did you ever receive reminders from your navigator for your upcoming scheduled PrEP visits?

1. Did you provide your navigator permission to trace you in person if you missed a PrEP visit?

1. Were you ever offered a PrEP re-start kit from your navigator? [as a reminder, this is an HIV-self test and, if you need them, a package of PrEP pills]

1. Is there anything else you would like to share about your experience working the navigator, your experience in the study or anything else?

1. Do you have any questions for me?

[END OF INTERVIEW FOR INTERVENTION PARTICIPANT]

**Appendix 2: In-depth Interview Guide for Key Stakeholders**

*[Read or paraphrase to participant]*

Thank you for agreeing to talk with me today. My name is [NAME]. I am part of a research team seeking to improve HIV prevention among men in Malawi, specifically through use of pre-exposure prophylaxis supported through a navigator. As you may know, pre-exposure prophylaxis (or PrEP) can help reduce the risk of an HIV-uninfected person becoming infected with HIV, if the PrEP medication is taken as prescribed. You have been asked to participate in this research given your expertise and familiarity and/or involvement with HIV prevention programming and/or policy in Malawi.

We want your honest opinions – there are no right or wrong answers, what we want to know is what you think and believe. If there are any questions that make you feel uncomfortable, feel free to tell me and we can skip or come back to those questions.

Anything that you say to me will be kept confidential. Our discussion will be audio recorded and then transcribed. When it is written out, all information that could identify you to someone reading the words (anything said with names or locations or that kind of thing) are completely removed. You may opt not to have the interview recorded if you wish. The material from our interview is saved with your identification number but not your name. The research team members reviewing this text will not link who you are to that ID and will not tell anyone on site about your specific feedback. There are other people doing interviews as well, and we will put all those documents together and look at main topics people talked about. We will not identify or share anything you say as a comment linked to your name or ID number.

Do you have any questions before we begin?

1. Can you please begin by telling me about your professional role and activities?
2. *What specifically is your role as it relates to HIV prevention policy or program development, implementation and evaluation?*

1. Can you please tell me what your familiarity with pre-exposure prophylaxis (PrEP) for HIV prevention is?
2. Are there populations that you feel particularly need PrEP but are not adequately accessing PrEP services in Malawi?

1. What do you feel like the role of integrated service delivery within STI clinics is?

1. How would you compare the risk of contracting HIV among STI clinic patients to other “key populations”, such as adolescent girls and young women, people who buy or sell sex, men who have sex with men, etc?

1. How do you counsel patients who present with an STI about their risk of HIV?
2. What has been your experience of persons coming to the STI clinic for management of STI syndromes and requesting PrEP?

1. What are some of the ways you have counseled, or would think about counseling, someone considering initiating PrEP?
2. What have you heard of alternatives to daily, oral PrEP for men?

1. When you are considering prescribing a PrEP modality for men other than daily oral PrEP, how do you decide to prescribe one PrEP option over another?

1. What did you think about event-driven PrEP when you first heard of it?

1. What did you think about CAB-LA when you first heard of it?

1. Thinking specifically about your clients currently on PrEP, how likely do you think they are to want to switch from daily oral to either event-driven oral or CAB-LA?

1. If a patient on daily oral PrEP is struggling with adherence, what would make you consider offering one of the other PrEP modalities?

1. In what ways do you feel that the needs of men differ from the needs of women when it comes to PrEP service delivery?
2. What types of venues or locales are ideal to engage men in HIV prevention services?

1. How have heterosexual men responded to the option of event-driven PrEP?

1. Event-driven PrEP was added into the guidelines, what motivated including event-driven PrEP in the revised PrEP guidelines for men?

1. If a male client is struggling to adhere to daily oral PrEP, what sort of messages or approaches to counseling do you focus on?

1. What are some of the unique barriers to PrEP uptake and persistent use among heterosexual men in Malawi?

1. Stigma can be a barrier to uptake and receipt of many services, including PrEP. In what way do you think stigma may be relevant for heterosexual men who are offered PrEP?
2. If you were to evaluate a program for men receiving PrEP, what metrics would you look at within a specific clinic to indicate that the program has been successful?

1. PrEP persistence is the challenge we are trying to address in our intervention. How do you define persistence as an outcome?

1. How important is PrEP persistence as an outcome of programming to you in general? How important is PrEP persistence, specifically for men? How does PrEP persistence compare to PrEP uptake as the outcome of interest in policies and programs?

As you may know, the Njira study is currently evaluating use of systems navigators as a strategy to improve PrEP persistence among heterosexual men who are seeking STI services and who are initiated on PrEP. A navigator can be thought of like a coach or a guide – using motivational interviewing to help a person set and meet personal goals, and helping with things like visit reminders or tracing for missed visits. The Njira intervention also offers what we are calling a “PrEP restart kit” for men who choose to stop PrEP – essentially this is an HIV self-test and a small packet of pills such that, if or when they resume behaviors that put them at risk of acquiring HIV, they can quickly and safely restart oral PrEP (before exposure), and subsequently re-engage in PrEP care.

1. Given this description of systems navigation, how likely do you think systems navigation as an intervention is, to address the unique barriers facing men with regard PrEP adherence?

1. Given this description, how or does systems navigation align with the PrEP priorities in Malawi? Why or why not?

1. Can you tell me what your first impression was when you heard about the fact that this intervention is specifically engaging heterosexual men?

1. How does the prioritization of men align within the existing culture at Bwaila STI clinic? What about the Bwaila clinic mission or priority areas*?*

1. Can you tell me what your experience or exposure to the systems navigators that are working with the Njira study has been?

1. How well do you think the Systems Navigators integrated into PrEP service delivery for study participants?

1. How well integrated did you feel into PrEP service delivery at the clinic?

1. In general, how did participants relate to you?

1. What was it like keeping track of all of your activities (including PrEP visit reminders, follow up for missing PrEP visits, conducting navigation sessions and STI testing, recruitment, offering self-test kits)?

1. What about the current Systems Navigation intervention do you feel worked particularly well?

1. What would success look like to you for full integration of systems navigators into the Bwaila PrEP clinic?

1. On a scale of 1 to 5 with 5 being “strongly agree” and 1 being “not agree at all” how much do you agree (or disagree) with the following statements:

| Systems navigation as part of PrEP care meets my approval | 1               2              3              4              5 |
| --- | --- |
| Systems navigation as part of PrEP care is appealing to me | 1               2              3              4              5 |
| I like systems navigation as part of PrEP care for men | 1               2              3              4              5 |
| I welcome systems navigation as part of PrEP care for men | 1               2              3              4              5 |

1. One of the roles of a navigator is to help remind PrEP users of upcoming appointments or to trace them if they miss an appointment. Are either of these tasks currently incorporated into PrEP care at Bwaila?
2. In order to increase persistence, one of the roles of a navigator is to help remind PrEP users of upcoming appointments or to trace them if they miss an appointment. How does this strategy align with other existing PrEP policies and programs in Malawi?

1. Another part of the systems navigation intervention was a PrEP “re-start kit” that included an HIV self-test kit and PrEP pills (for patients on daily, oral and event-driven PrEP who choose to disengage from care). If PrEP re-start kits were to be implemented in the Bwaila PrEP clinic, what are the main benefits you think may come from these PrEP re-start kits?

1. Another part of the systems navigation intervention was a PrEP “re-start kit” that included an HIV self-test kit and PrEP pills (for patients on daily, oral and event-driven PrEP who choose to disengage from care). If PrEP re-start kits were to be implemented in the Malawi PrEP program, what are the main benefits you think may come from these PrEP re-start kits?
2. Navigators also provide testing for gonorrhea and chlamydia to participants they are working with. What are some of the barriers to incorporating this kind of etiologic testing into the Bwaila STI clinic?
3. Navigators also provide testing for gonorrhea and chlamydia to participants they are working with. What are some of the barriers to incorporating this kind of etiologic testing into PrEP clinics in Malawi?
4. The primary outcome of the Njira study is looking at persistent use of PrEP, defined as a combination of a participant remaining engaged in PrEP care and adherent to their prescribed PrEP modality. Are there other outcomes that you would want to know about to demonstrate the benefit or impact of systems navigators to inform PrEP program implementation in Malawi?

1. The study also evaluates the cost of this program – how would knowing the total cost of operating this program at a given clinic be important to any decision making? What about other metrics of cost, such as a comparator of, say, dollar per additional man persistent on PrEP?

1. Right now, the navigators supported through the Njira study are intentionally selected as persons who resemble the target population in terms of age and gender. Navigators also have some experience in counseling, such as certification in HTS, but are not nurses and more closely resemble lay providers. Are there similar programs that have been used for PrEP care in Malawi to date?

1. You were selected as a navigator because of your identification as a heterosexual male and with some experience in HIV and STI-related counseling. Did you feel like the training you received was enough to conduct your job as a systems navigator?
2. If systems navigators were to be incorporated into the Bwaila PrEP clinic, what kind of local partnerships would be key for buy-in?

1. Before we end, I would like you to think back to the time that PrEP services were disrupted for men at Bwaila STI clinic (in February of this year, 2025). What was the impact of this PrEP disruption?
2. If systems navigators were to be incorporated in clinics throughout Malawi, what kind of local partnerships would be key for buy-in?

1. Is there anything else you would like to add related to PrEP programming in general or use of systems navigators for heterosexual men in Malawi?

1. Do you have any questions for me?

[END INTERVIEW]
