# Supplementary material for: Improving HIV Prevention Among Heterosexual Men Seeking Sexually Transmitted Infection Services in Malawi: Protocol for a Type I Effectiveness-Implementation Hybrid Randomized Controlled Trial of Systems Navigator–Delivered Integrated Prevention Package (HPTN 112-NJIRA Study)
Source: JMIR Res Protoc. 2025 Jun 18;14:e72981. doi: 10.2196/72981 (PMC12223453; doi:10.2196/72981)
Supplement: Multimedia Appendix 3 [file resprot_v14i1e72981_app3.docx]

**SUPPLEMENTAL FILE S2: INFORMED CONSENT FORMS**

**University of North Carolina at Chapel Hill**
**Consent to Participate in a Research Study**
**Adult Participants**
______________________________________________________________________________

**Consent Form Version Date:** June 24 2024
**IRB Study # 23-2337**

**NHSRC Protocol #23/10/4208**

**Title of Study:** HPTN 112: Improving HIV prevention among heterosexual cisgender men seeking STI services in Malawi: examining the benefits, acceptability, and associated costs of a systems-navigator- delivered integrated prevention package

**Principal Investigator (US): Sarah Rutstein, MD, PhD**

**Principal Investigator (Malawi): Mitch Matoga, MBBS, MS**
**Principal Investigator Department:** Department of Medicine

**Principal Investigator Phone number (US):** (919) 966-2537
**Principal Investigator Email Addresses:** srutstein@unc.edu; mmatoga@unclilongwe.org

**Funding Source and/or Sponsor:** Division of AIDS (DAIDS), United States (US) National Institute of Allergy and Infectious Diseases (NIAID), US National Institutes of Health (NIH)

**Study Contact Telephone Number**: +265 1 755 056
**Study Contact Email**: mmatoga@unclilongwe.org

______________________________________________________________________________

**CONCISE SUMMARY:** This is a research study. Taking part in this research study is voluntary (your choice). You do not have to participate, and you can leave the study at any time. No matter what you decide, any other care that you get at this site will not change.

This study is testing whether adding a systems navigator (similar to a coach or guide) to pre-exposure prophylaxis (PrEP) care, a medicine that can help prevent HIV, improves PrEP use among heterosexual men, compared to the current standard PrEP care.

The study will take about 15-months total. If you choose to enroll in the study, you will be followed on the study for at least 6 months and up to 12 months. You will be asked to give blood and urine specimens for HIV, STI, and other tests. You do not need to remain on PrEP to continue participation in this study.

There are very limited risks involved with this study, including (but not limited to) risk of discomfort, dizziness/faintness, and/or bruising, swelling and/or infection from your blood being taken. You may also feel feelings of embarrassment or worry when answering questions about your own behaviors and/or receiving HIV counseling, or become worried while waiting for your HIV test results. Finally, there is the potential that some of your information may become known to others, even though the study will do everything possible to prevent that.

There may be no direct benefits to you for being in this study, but others may benefit in the future from information learned from this study. Specifically, information on how to improve HIV prevention services for heterosexual men. In addition, you will receive HIV and STI counseling and testing as part of the study process and will be referred for treatment as needed.

More information about this study is described in the rest of this form. We will help you understand the information and answer all your questions. You should feel that you understand the study before deciding whether you will participate. If you agree to join the study, you will be asked to sign your name or make your mark on this form. We will offer you a copy of this form to keep.

**What are some general things you should know about research studies?**

You are being asked to take part in a research study. To join the study is voluntary. You may choose not to participate, or you may withdraw your consent to be in the study, for any reason, without penalty.

Research studies are designed to obtain new knowledge. This new information may help people in the future. You may not receive any direct benefit from being in the research study. There also may be risks to being in research studies. Deciding not to be in the study or leaving the study before it is done will not affect your relationship with the researcher or the health care provider.  If you are a patient with an illness, you do not have to be in the research study in order to receive health care.

Details about this study are discussed below. It is important that you understand this information so that you can make an informed choice about being in this research study.

You will be given a copy of this consent form. You should ask the researchers named above, or staff members who may assist them, any questions you have about this study at any time.

**What is the purpose of this study?**

The main purpose of this research study is to understand if it is helpful to use a systems navigator (coach or guide) to support the use of PrEP to prevent HIV. The overall goal of this study is to collect data that will inform future research, helping men from STI clinics reduce risk of getting HIV by taking PrEP.

This study is for adult and adolescent cisgender men who:

- - are age 15 years or older,
  - are seeking services at Bwaila STI Clinic, and
  - have tested HIV negative

**Are there any reasons you should not be in this study?**

You should not be in this study if you are taking part in another study of drugs or medical devices. You are asked to tell the study staff about any other studies you are taking part in or thinking of taking part in. This is very important for your safety.

**How many people will take part in this study?**

About 200 HIV-seronegative heterosexual men will participate in this study from Lilongwe, Malawi.

**How long will your part in this study last?**

Participants who choose to join the study will be in the study for at least 6 months and up to 12 months total.

**What will happen if you take part in the study?**

During this study, you will be in one of 2 study groups. Researchers will "randomize" you into one of the study groups described here. 2 out of every 3 persons enrolled will be assigned to the intervention group and 1 out of every 3 persons enrolled will be assigned to the standard of care group. Random assignment means that you are put into a study group by chance, like flipping a coin. Neither you nor the study staff can choose your study group. The table below indicates what services each group will receive.

| Standard of Care Group | Standard PrEP services, including HIV testing and risk reduction counseling |
| --- | --- |
| Intervention Group | Standard PrEP services, including HIV testing and risk reduction counseling. You will also be assigned a systems navigator (coach), respond to a brief questionnaire about your sexual activities, and be tested for STIs at any PrEP visit you attend. This coach may also attempt to contact or trace you for any missed PrEP visit (~7 days late), using information you provide regarding your preferred tracing time, method (phone or in-person), and location. |

PrEP visits: If you continue on PrEP, the timing of PrEP visits will be decided by the PrEP nurse at the clinic, according to the Malawi guidelines for PrEP. These visits typically occur every 2-3 months, depending on the kind of PrEP you are taking.

This study is using a Standard of Care Group (also known as a “Control Group”) because we do not yet know whether the intervention being tested in this study improves PrEP use. This kind of intervention has not previously been tested in PrEP care among men in Malawi. Using a Standard of Care Group will allow researchers to assess whether providing the additional intervention resources improves PrEP use. That information can be used to make recommendations to health care providers and leaders about what services should be offered in the future.

All participants will complete the study visit procedures outlined below, regardless of which group they are assigned.

Study visits: The study has at least 3 visits (including the enrollment visit today). Besides this first visit, you will be asked to come back for a visit ~3 months and ~6 months from now. If you enroll early in the study, you may also be asked to come back for study visits ~9 months and ~12 months from now. At each visit, you will be asked a series of questions about your behaviors and be tested for HIV and other STIs. You may also be asked to participate in a one-on-one interview. You should attend these study visits even if you have decided to stop taking PrEP.

***Enrolling in the Study***

If you decide to take part in the study, the Enrollment visit will last about 1-2 hours. During the Enrollment Visit, we will:

- Obtain full written informed consent for the study
- Ask you where you live and how to contact you
- Ask you to answer questions in a survey about your sexual activities and overall health
- Ask you about your previous and current PrEP use
- Provide brief HIV risk reduction counseling
- Conduct a symptom-driven physical exam
- Collect ~10 to 20 mL (about 2 to 4 teaspoons) of blood to make sure you do not have a very early infection with HIV that may not be detected by the standard HIV tests you’ve received in clinic. If you have not been tested for syphilis (another STI) at any recent PrEP visits, this blood will also be used to test you for syphilis.
- Collect ~ 10mL of your urine to test for STI testing (Chlamydia and Gonorrhea)
- Store blood (plasma, dried blood spots) and urine samples for study-related testing.
- If you are assigned to the intervention group, study staff will connect you to your coach to discuss next steps in your PrEP care

***Additional Visits: 2 to 4 additional visits over the next 6 to 12 months.***

If you decide to join the study, after your Enrollment Visit, you will be asked to come to this location for follow up visits. Participants will be followed for at least 6 months and up to 12 months depending on the time of enrollment.

Each visit will last about 1 hour.

During these follow-up visit(s), we will:

- Ask you where you live and how to contact you, only if the information you gave before has changed.
- Ask you to answer questions in a survey about your sexual activities and overall health
- Ask you questions about if you are still using PrEP, why, or why not
- Provide HIV risk education counseling
- Conduct a symptom-driven physical exam
- Collect ~10 to 20 mL (about 2 to 4 teaspoons) of blood. Your blood will be used to test you for HIV. If you have not been tested for syphilis at any recent PrEP visits, this blood will also be used to test you for syphilis
- Collect ~ 10mL of your urine to test for STI testing (Chlamydia and Gonorrhea) if you have not been tested for these infections at any recent PrEP visits
- Collect ~ 5 strands of hair, plucking 5 strands of hair from the back of your head with tweezers, so that we can look at medication in the hair root. It will be chosen from a discrete location and be hidden as much as possible. Hair samples will only be collected if you have sufficient hair (1.0cm) for collection.
- Store blood (plasma, dried blood spots), urine samples, and hair samples for study-related testing

At your final study visit, we will talk with you about the end of the study and when the results of the study will be available. You may also be asked to participate in a one-on-one interview around the time of your final study visit. Approximately 20 participants will be asked to participate in this type of interview. This would add 30-60 minutes to your visit time, and you would be reimbursed for this additional time. The interviewer will ask you questions about your understanding about HIV prevention, including PrEP, your experience being part of this study, and any challenges you experienced engaging in HIV prevention. If you are assigned to the intervention group, you will also be asked about your experience working with the PrEP coach. The interview will be audiotaped so that your responses can be transcribed for analysis. During transcription, any identifying names or places in the interview will be removed from the transcript. After transcription, the recording of the interview will be destroyed. Analysis will be conducted to look for common themes in all interviews conducted. You can decline participation in this interview.

If you test positive for HIV at any point in the study, you will be referred for HIV care and encouraged to start treatment for HIV immediately and you will have an additional approximately 6-months of study follow-up time from the time you test positive. During this time, you will have blood collected twice – once at 3 months and once at 6 months – to make sure that your body is responding appropriately to HIV treatment. During these visits, we will draw 10-20 mL (about 2-4 teaspoons) of blood to check the amount of HIV in your body. We will ask you about any medications you are taking, including for HIV treatment. We will also ask you questions about personal perceptions. If you have not started HIV treatment yet, we will encourage you to start. We will also update your contact information. This could happen at a final visit if you want to stop the study early.

**If you stop taking PrEP, we will ask you to stay in the study*.***

If you permanently stop taking PrEP during the study for any reason, we will ask you to continue to come for your regular study visits, but you will no longer have to undergo certain procedures, like answering questions about taking PrEP, etc. Similarly, if you or miss doses of PrEP or miss PrEP visits, we will ask you to continue to come for your regular study visits as scheduled.

**If you get HIV during the study, we will help you get care and support.**

We will test your blood for HIV during this study. If you get HIV while you are in the study, you will stop taking PrEP, and we will help you find the care and support you need.

**Use of stored samples**

In addition to the laboratory testing performed at each study visit, further study-related testing may be performed on blood, urine, and hair samples. This will include testing related to HIV and other infections, including testing for anti-HIV medications and for quality control testing (to confirm results obtained in laboratories). If you are found to have a very early phase of HIV or get infected with HIV during the study, some blood may also be used to learn more about HIV viruses, the body’s response to HIV infection, and how HIV is spread in the community. The samples used for this testing will be labeled with your study number and will be tested at special laboratory facilities that may be located in the US and other countries outside of Malawi. The laboratory doing the testing will not know who you are. Only approved researchers will have access to your samples. Results of this specialized testing will not be returned to the study site or you. Your samples will not be sold or directly used to produce commercial products or for commercial gain. No host genetic testing will be tested from these samples. Host testing means genetic testing derived from you.

**What are the possible benefits from being in this study?**

There may be no direct benefits to participants in this study, however, participants and others may benefit in the future from information learned from this study. Specifically, information learned in this study may lead to improved HIV prevention services for heterosexual men in Malawi and the African region. In addition, participants will receive HIV and STI counseling and testing as part of the study process. Participants also will be referred for treatment if needed. Participants who choose to continue PrEP may benefit from the use of PrEP medicines which are known to protect against getting HIV if taken as directed.

**If you choose not to be in the study, what other treatment options do you have?**

Your participation is voluntary. You do not have to take part in any of the tests or procedures in the study. You should also know that:

- If you decide not to join the study, you will not lose your regular medical care.
- If you join this study and later decide to leave, you will not lose your regular medical care.
- You do not have to join the study to receive HIV prevention medications.
- If you decide not to join the study, you will still be able to join another study at a later time if there is one available and you qualify.

**Can I change my mind about participating in this study?**

Yes, you can change your mind at any time. Your participation in this study is completely up to you (voluntary). Your decision to leave the study will not lead to any penalty, or loss of benefits or rights that you would normally have otherwise.

**What are the possible risks or discomforts involved from being in this study?**

It is not expected that this study will expose you to unreasonable risk. Blood draws may lead to discomfort, feelings of dizziness or faintness, and/or bruising, swelling, and/or infection. There is no foreseeable risk with the donation of hair. You may become embarrassed, worried, or anxious when completing their HIV risk assessment and/or receiving HIV counseling. You also may become worried or anxious while waiting for their HIV test results. Trained counselors will be available to help you deal with these feelings. Although the site will make every effort to protect your privacy and confidentiality, it is possible that your involvement in the study could become known to others, and that social harms may result. Social harms could occur if you are perceived as having HIV or at increased likelihood of getting HIV. Examples of social harms are when you are treated unfairly or have problems being accepted by your families and/or communities (i.e., because you could become known as vulnerable to HIV).

**What if we learn about new findings or information during the study?**

You will be told any new information learned during this study that might affect your willingness to stay in the study. For example, if information becomes available that shows that the PrEP coach may have bad effects, you will be told about this. You will also be told when the results of the study may be available, and how to learn about them.

**How will information about you be protected?**

Every effort will be made to keep your personal information confidential, but we cannot guarantee absolute confidentiality. To keep your information private, your samples will be labeled with a code that can only be traced back to the study clinic. The results of any tests done on these samples will not be included in your health records. Your name, where you live, and other personal information will be protected by the study clinic. You will be identified by a code, and personal information from your records will not be released without your written permission. Any publication of this study will not use your name or identify you personally. Your personal information may be disclosed if required by law.

Clinic staff will have access to your study records. Your records may also be reviewed, under guidelines of the US Federal Privacy Act, by:

The Malawi National Health Science Research Committee (NHSRC)

The University of North Carolina at Chapel Hill (UNC) Institutional Review Board (IRB)

The sponsor of the study (US National Institutes of Health [NIH]) and its contractors.

The US Office for Human Research Protections (OHRP)

Other local, US, or international regulatory authorities/entities

The HPTN (HIV Prevention Trials Network) that is conducting this study

The study staff will also use your personal information, if needed, to verify that you are not taking part in any other research studies. This includes other studies conducted by Bwaila STI Clinic and studies conducted by other researchers that study staff know about.

Malawi regulations require study staff to report the names of people who get HIV to the local health authority. Outreach workers from the health authority may then contact you about informing your partners, since they also should be tested. If you do not want to inform your partners yourself, the outreach workers will offer to contact them, according to the confidentiality guidelines of the Malawi Ministry of Health.

A description of this clinical trial will be available on <http://www.ClinicalTrials.gov>. This Web site will not include information that can identify you. At most, the Web site will include a summary of the study results. You can search this Web site at any time.

**What is a Certificate of Confidentiality?**

Most people outside the research team will not see your name on your research information. This includes people who try to get your information using a court order in the United States. One exception is if you agree that we can give out research information with your name on it or for research projects that have been approved under applicable rules. Other exceptions are for information that is required to be reported under law, such as information about certain harmful diseases that can be spread from one person to another. Personnel of a government agency sponsoring the study may also be provided information about your involvement in the research study.

**What will happen if you are injured by this research?**

If you get sick or injured during the study, contact us immediately.

It is unlikely that you will be injured as a result of study participation. If you are injured, the Bwaila STI Clinic will give you immediate necessary treatment for your injuries. You will not have to pay for this treatment. You will be told where you can get additional treatment for your injuries. There is no program to pay money or give other forms of compensation for such injuries either through this institution or the US NIH. You do not give up any legal rights by signing this consent form.

If you think you have been injured from taking part in this study, call the Principal Investigator at the phone number provided on this consent form. They will let you know what you should do.

**What if you want to stop before your part in the study is complete?**

You can withdraw from this study at any time, without penalty.  The investigators also have the right to stop your participation at any time. This could be because you have had an unexpected reaction, or have failed to follow instructions, or because the entire study has been stopped. If you decide to withdraw, we will ask you to come in for a final visit for collection and storage of blood and urine and STI testing and referral

**Will you receive anything for being in this study?**

You will receive the equivalent of approximately 12USD for your time, effort, and travel to and from the clinic at each scheduled study visit.

**Will it cost you anything to be in this study?**

There will be no cost to you for study related visits, physical examinations, laboratory tests, or other procedures.

**Who is sponsoring this study?**

This research is funded by Family Health International (FHI 360) through a grant from the US National Institutes of Health (NIH). This means that the research team is being paid by the sponsor for doing the study. The researchers do not, however, have a direct financial interest with the sponsor or in the final results of the study.

**What if you have questions about this study?**

If you ever have any questions about the study, want to report a social harm, or if you have a research- related injury, you should contact Dr. Mitch Matoga at +265 999 511 726.

If you have questions about your rights as a research participant, you should contact the Head of Secretariat at the National Health Science Research Committee, Dr. Evelyn Chitsa Banda, at +265 999 93 69 37 or email at chitsabandaeve@yahoo.com.

By mail:

The Head of Secretariat

Malawi National Health Science Research Committee (NHSRC)

Ministry of Health

Phone: +265 726 422

or by email: [mohdoccentre@gmail.com](mailto:mohdoccentre@gmail.com)

**What if you have questions about your rights as a research participant?**

All research on human volunteers is reviewed by a committee that works to protect your rights and welfare. If you have questions or concerns about your rights as a research subject, or if you would like to obtain information or offer input, you may contact the Institutional Review Board at 919-966-3113 or by email to [IRB_subjects@unc.edu.](mailto:IRB_subjects@unc.edu)

**Consent SIGNATURE PAGE**

If you have read this consent form, or had it read and explained to you, you understand the information, and you voluntarily agree to join the study, please sign your name or make your mark below.

____ I agree to take part in this study.

____ I do not agree to take part in this study.

Hair specimen collection (follow-up visits only):

____ I agree to have hair collected.

____ I do not agree to have hair collected.

If selected for an interview:

____ If selected, I agree to participate in an interview where I will be asked questions about this research, and the interview will be recorded.

____ If selected, I do not agree to participate in an interview where I will be asked questions about this research.

**PART A: LITERATE PARTICIPANT**

***Participant is literate***:

________________________________ _____________________________________

Participant Name (print) Participant Signature and Date

________________________________ _____________________________________

Study Staff Conducting Consent Discussion (print) Study Staff Signature and Date

**PART B : ILLITERATE PARTICIPANT**

***Participant is illiterate:***

The **impartial witness must write participant’s name and date of consent** below**.**

Please thumbrint here if you will allow us to audio record interviews :

Mark or Thumbprint of participant if unable to sign

***Yes : No :***

Mark or Thumbprint of participant if unable to sign

Mark or Thumbprint of participant if unable to sign

Participant Mark or Thumbprint

Participant Name (print) Date

Participant Name and Date Written by………………………………….on…...………

_____________________________ ____________________________ ___________

Study Staff Conducting Consent Study Staff Signature Date

Discussion (print)

_____________________________ _____________________________ ___________

Impartial Witness Name Impartial Witness Signature Date

(print)

**University of North Carolina at Chapel Hill
Assent to Participate in a Research Study
Adolescent Participants age 15-17**
______________________________________________________________________________

**Consent Form Version Date:** 24 June 2024

**IRB Study # 23-2337**

**NHSRC Protocol #23/10/4208**

**Title of Study:** HPTN 112: Improving HIV prevention among heterosexual cisgender men seeking STI services in Malawi: examining the benefits, acceptability, and associated costs of a systems-navigator- delivered integrated prevention package

**Principal Investigator (US): Sarah Rutstein, MD, PhD**

**Principal Investigator (Malawi): Mitch Matoga, MBBS, MS**
**Principal Investigator Department:** Department of Medicine

**Principal Investigator Phone number (US):** (919) 966-2537
**Principal Investigator Email Addresses:** srutstein@unc.edu; mmatoga@unclilongwe.org

**Funding Source and/or Sponsor:** Division of AIDS (DAIDS), United States (US) National Institute of Allergy and Infectious Diseases (NIAID), US National Institutes of Health (NIH)

**Study Contact Telephone Number**: +265 1 755 056
**Study Contact Email**: mmatoga@unclilongwe.org

______________________________________________________________________________

**CONCISE SUMMARY:** This is a research study. Taking part in this research study is voluntary (your choice). You do not have to be in the study, and you can leave the study at any time. No matter what you decide, any other care that you get here will not change.

**This study is about systems navigators for PrEP for HIV (Human Immunodeficiency Virus).**

- PrEP is short for Pre-Exposure Prophylaxis.
- Pre-exposure means before coming into contact with HIV. Prophylaxis is the way people prevent a disease from infecting them.
- A Systems Navigator is like a coach or guide to help with access and use of PrEP.
- With PrEP for HIV, medications are used to prevent people from getting HIV if they are exposed to it.

The study will take about 15 months total. If you choose to be in the study, you will be followed on the study for at least 6 months and up to 12 months. You will be asked to give blood and urine specimens for HIV, sexually transmitted infections (STI), and other tests. You do not need to remain on PrEP to continue being in this study.

There are very few risks with this study, including (but not limited to) risk of feeling uncomfortable, dizziness/faintness, and/or bruising, swelling and/or infection from your blood being taken. You may also feel feelings of embarrassment or worry when answering questions about your own behaviors and/or getting HIV counseling, or become worried while waiting for your HIV test results. Finally, it is possible that some of your information may become known to others, even though the study will do everything possible to prevent that.

There may be no direct benefits to you for being in this study, but others may benefit in the future from information learned from this study. Information on how to improve HIV prevention services for men. In addition, you will receive HIV and STI counseling and testing as part of the study process and will be referred for care as needed.

More information about this study is found in the rest of this form. We will help you understand the information and answer all your questions. You should feel that you understand the study before deciding whether you will participate. If you agree to join the study, you will be asked to sign your name or make your mark on this form. Your parent or guardian will also need to sign a form saying that it is ok for you to join the study. We will offer you a copy of this form to keep.

**What are some basic things you should know about research studies?**

You are being asked to take part in a research study. To join the study is your choice. You may choose not to be in the study, or you may leave the study at any time, for any reason, without punishment.

Research studies are designed to obtain new information. This new information may help people in the future. You may not receive any direct benefit from being in the research study. There also may be risks to being in research studies. Deciding not to be in the study or leaving the study before it is done will not affect your relationship with the researcher or the health care provider.  If you are a patient with an illness, you do not have to be in the research study in order to receive health care.

More information about this study is discussed below. It is important that you understand this information so that you can make the best choice for you about being in this research study.

You will be given a copy of this consent form. You should ask the researchers named above, or staff members who may assist them, any questions you have about this study at any time.

**Why are we doing this study?**

The main reason for this research study is to understand if it is helpful to use a systems navigator (coach or guide) to help in the use of PrEP to keep someone from getting HIV. The overall goal of this study is to gather information that will help us in future research, helping men from STI clinics lower their chances of getting HIV by taking PrEP.

This study is for adult and adolescent men who:

- - are age 15 years or older,
  - are seeking services at Bwaila STI Clinic, and
  - have tested HIV negative

**Are there any reasons you should not be in this study?**

You should not be in this study if you are taking part in another study of drugs or medical medical tools or equipment. You are asked to tell the study staff about any other studies you are in or thinking of being in. This is very important for your safety.

**How many people will take part in this study?**

About 200 men who do not have HIV will participate in this study from Lilongwe, Malawi.

**How long will your part in this study last?**

Men who choose to join the study will be in the study for at least 6 months and up to 12 months total.

**What will happen if you take part in the study?**

During this study, you will be in one of 2 study groups. Researchers will "randomize" you into one of the study groups described below. 2 out of every 3 persons in the study will be part of the group with the coach/guide (intervention group) and 1 out of every 3 persons in the study will be part of the regular care (standard of care group). Random assignment means that you are put into a study group by chance, like flipping a coin. Neither you nor the study staff can choose your study group. The table below indicates what services each group will receive.

| Standard of Care Group | Regular PrEP services, including HIV testing and counseling to help you lower your chance of getting HIV |
| --- | --- |
| Intervention Group | Regular PrEP services, including HIV testing and counseling to help you lower your chance of getting HIV. You will also be assigned a systems navigator (coach), answer a brief questionnaire about your sexual activities, and be tested for STIs at any PrEP visit you attend. This coach may also attempt to contact or trace you for any missed PrEP visit (~7 days late), using information you provide regarding your desired tracing time, type (phone or in-person), and location. |

PrEP visits: If you stay on PrEP, the timing of PrEP visits will be decided by the PrEP nurse at the clinic, according to the Malawi directions for PrEP. These visits usually occur every 2-3 months, depending on the kind of PrEP you are taking.

This study is using a Standard of Care Group (also known as a “Control Group”) because we do not yet know whether adding the coach for PrEP care, which is the intervention in this study, improves PrEP use. This kind of intervention has not been tested in PrEP care among men in Malawi before. Using a Standard of Care Group will allow researchers to tell whether providing the extra intervention resources improves PrEP use. That information can be used to improve instructions for health care providers and leaders about what services should be offered in the future.

All people in this study will finish the study visit activities given below, no matter which group they are in.

Study visits: The study has at least 3 visits (including this enrollment or first visit today). Besides this first visit, you will be asked to come back for a visit ~3 months and ~6 months from now. If you enroll early in the study, you may also be asked to come back for study visits ~9 months and ~12 months from now. At each visit, you will be asked a series of questions about your behaviors and be tested for HIV and other STIs. You may also be asked to participate in an interview with one other person asking you questions. You should come to these study visits even if you have decided to stop taking PrEP.

***Enrolling in the Study***

If you decide to be in the study, the Enrollment (first) visit will last about 1-2 hours. During the Enrollment Visit, we will:

- Ask you if you agree to be in the study and ask you to sign this form
- Ask you where you live and how to contact you
- Ask you to answer questions in a survey about your sexual activities and overall health
- Ask you about your PrEP use now and in the past
- Provide short counseling to help keep you from getting HIV
- Conduct an exam of your body if you have any issues
- Collect ~10 to 20 mL (about 2 to 4 teaspoons) of blood to make sure you do not have a very early infection with HIV that we might not see with the regular HIV tests you’ve gotten in clinic. If you have not been tested for syphilis (another STI) at any recent PrEP visits, this blood will also be used to test you for syphilis.
- Collect ~ 10mL (about 2 teaspoons) of your urine to test for STI testing (Chlamydia and Gonorrhea)
- Store blood (plasma, which is part of your blood, and dried blood spots) and urine for study testing
- If you are in the intervention (coach) group, study staff will connect you to your coach to talk about next steps in your PrEP care

***Additional Visits: 2 to 4 more visits over the next 6 to 12 months.***

If you decide to join the study, after your Enrollment Visit, you will be asked to come to this location for more visits (called follow-up visits). You will be followed for at least 6 months and up to 12 months depending on when you join the study.

Each visit will last about 1 hour.

During these follow-up visit(s), we will:

- Ask you where you live and how to contact you, only if the information you gave before has changed
- Ask you to answer questions in a survey about your sexual activities and overall health
- Ask you questions about if you are still using PrEP, why, or why not
- Provide counseling to help keep you from getting HIV
- Conduct an exam of your body if you have any issues
- Collect ~10 to 20 mL (about 2 to 4 teaspoons) of blood. Your blood will be used to test you for HIV. If you have not been tested for syphilis (another STI) at any recent PrEP visits, this blood will also be used to test you for syphilis
- Collect ~ 10mL (about 2 teaspoons) of your urine to test for STI testing (Chlamydia and Gonorrhea) if you have not been tested for these infections at any recent PrEP visits
- Collect ~ 5 strands of hair, plucking 5 strands of hair from the back of your head with tweezers, so that we can look at medication in the hair root. It will be chosen from a discrete location and be hidden as much as possible. Hair samples will only be collected if you have sufficient hair (1.0cm) for collection.
- Store blood (plasma, which is part of your blood, and dried blood spots), urine, and hair samples for study testing

At your final study visit, we will talk with you about the end of the study and when the results of the study will be known. You may also be asked to be in an interview with one other person asking you questions around the time of your final study visit. About 20 people in this study will be asked to be in this type of interview. This would add 30-60 minutes to your visit time, and you would be given money for this additional time. The interviewer will ask you questions about your understanding about HIV prevention, including PrEP, your experience being part of this study, and any problems you had with HIV prevention. If you are assigned to the intervention (coach) group, you will also be asked about your experience working with the PrEP coach. The interview will be audiotaped (recorded) so that your answers can be written down in a report for review later. When we write down your answers, any names or places that you say in the interview will be taken out of the written report. After the written report is done, the recording of the interview will be deleted and removed from all storage. A full understanding of the written report will take place to look for common ideas in all interviews we have for this study. You can say no to doing this interview.

If you test positive for HIV at any point in the study, you will be referred for HIV care and encouraged to start treatment for HIV immediately and you will have about 6-months more of study follow-up time from the time you test positive. During this time, you will have blood collected twice – once at 3 months and once at 6 months – to make sure that your HIV treatment is working. During these visits, we will draw 10-20 mL (about 2-4 teaspoons) of blood to check the amount of HIV in your body. We will ask you about any medicines you are taking, including for HIV treatment. We will also ask you questions about personal perceptions. If you have not started HIV treatment yet, we will encourage you to start. We will also update your contact information. This could happen at a final visit if you want to stop the study early.

**If you stop taking PrEP, we will ask you to stay in the study*.***

If you stop taking PrEP for good during the study for any reason, we will ask you to continue to come for your regular study visits, but you will no longer have to do certain tasks, like answering questions about taking PrEP. Also, if you miss doses of PrEP or miss PrEP visits, we will ask you to continue to come for your regular study visits as scheduled.

**If you get HIV during the study, we will help you get care and support.**

We will test your blood for HIV during this study. If you get HIV while you are in the study, you will stop taking PrEP, and we will help you find the care and support you need.

**Use of stored (kept) samples**

In addition to the laboratory testing done at each study visit, more study testing may be performed on your blood, urine, and hair samples. This will include testing related to HIV and other infections, including testing for anti-HIV medicines and for testing to confirm answers found from your blood in the laboratory). If you are found to have the very beginning of HIV or get infected with HIV during the study, some blood may also be used to learn more about HIV viruses, how the body reacts to HIV infection, and how HIV is spread in the community. The samples used for this testing will be labeled with your study number and will be tested at special laboratories that may be located in the US and other countries outside of Malawi. The laboratory doing the testing will not know who you are. Only approved scientists will have access to your samples. Results of this special testing will not be returned to this clinic or you. Your samples will not be sold or directly used to make products for money or used to make money in general. No host genetic testing will be tested from these samples. Host testing means genetic testing derived from you. Genetic testing is when scientists look closely at your DNA (genes).

**What are the possible benefits from being in this study?**

There may be no direct benefits to participants in this study, however, participants and others may benefit in the future from information learned from this study. Information learned in this study may lead to better HIV prevention services for men in Malawi and Africa. In addition, participants will receive HIV and STI counseling and testing as part of the study. Participants also will be sent for treatment if needed. Participants who choose to stay on PrEP may benefit from the use of PrEP medicines which are known to protect against getting HIV if taken the right way.

**If you choose not to be in the study, what other treatment options do you have?**

Your participation is your choice. You do not have to take part in any of the tests or activities in the study. You should also know that:

- If you decide not to join the study, you will not lose your regular medical care.
- If you join this study and later decide to leave, you will not lose your regular medical care.
- You do not have to join the study to receive HIV prevention medications like PrEP.
- If you decide not to join the study, you will still be able to join another study at a later time if there is one available and you qualify.

**Can I change my mind about participating in this study?**

Yes, you can change your mind at any time. Your participation in this study is completely up to you (voluntary). Your decision to leave the study will not lead to any punishment or loss of benefits or rights that you would normally have otherwise.

**What are the possible risks or discomforts involved from being in this study?**

We do not think that this study will expose you to too much risk. Blood being taken may lead to feeling uncomfortable, feelings of dizziness or faintness, and/or bruising, swelling, and/or infection. There is no foreseeable risk with the donation of hair. You may become embarrassed, worried, or nervous when answering questions about your chances of getting HIV and/or getting HIV counseling. You also may become worried or nervous while waiting for your HIV test results. Trained counselors will be available to help you deal with these feelings. Although the site will make every effort to keep your information safe, it is possible that your being in the study could become known to others, and that social harms may result. Social harms could occur if you are thought to have HIV or thought to have more of a chance of getting HIV. Examples of social harms are when you are treated unfairly or have problems being accepted by your families and/or communities (i.e., because you could be thought to have more of a chance to get HIV).

**What if we learn about new findings or information during the study?**

You will be told any new information learned during this study that might change whether you stay in the study or not. For example, if information becomes available that shows that the PrEP coach may have bad effects, you will be told about this. You will also be told when the results of the study may be available, and how to learn about them.

**How will information about you be kept safe?**

We won’t tell anyone else that you are in the study. We won’t share anything we find out about you with anyone, even your parent or guardian, without talking to you first. We will keep your personal information safe, but we cannot be absolutely sure it will be kept safe. We use a code number instead of your name on all the study forms. The results of any tests done on your samples will not be included in your health records. Your name, where you live, and other personal information will be protected by the study clinic. Any written report from this study will not use your name or say who you are. Your personal information may be given to others if needed by law.

Clinic staff will have access to your study records. Your records may also be reviewed, under rules of the US Federal Privacy Act, by:

The Malawi National Health Science Research Committee (NHSRC)

The University of North Carolina at Chapel Hill (UNC) Institutional Review Board (IRB)

The sponsor of the study (US National Institutes of Health [NIH]) and its workers

The US Office for Human Research Protections (OHRP)

Other local, US, or international regulatory authorities/entities

The HPTN (HIV Prevention Trials Network) that is conducting this study

The study staff will also use your personal information, if needed, to make sure that you are not taking part in any other research studies. This includes other studies run by Bwaila STI Clinic and studies run by other scientists that study staff know about.

Malawi rules make sure that study staff report the names of people who get HIV to the local health authority. If you get HIV, outreach workers from the health authority may then contact you about telling your partners you have HIV, since they also should be tested. If you do not want to tell your partners yourself, the outreach workers will offer to contact them, according to the privacy rules of the Malawi Ministry of Health.

A description of this clinical trial will be available on <http://www.ClinicalTrials.gov>. This Web site will not include information about you. At most, the Web site will include an overall view of the study results. You can search this Web site at any time.

**What is a Certificate of Confidentiality?**

Most people outside the research team will not see your name on your research information. This includes people who try to get your information using a court order in the United States. One exception is if you agree that we can give out research information with your name on it or for research projects that have been approved under applicable rules. Other exceptions are for information that is required to be reported under law, such as information about certain harmful diseases that can be spread from one person to another. Personnel of a government agency sponsoring the study may also be provided information about your involvement in the research study.

**What will happen if you are injured by this research?**

If you get sick or injured during the study, contact us immediately.

It is not likely that you will be injured because of study participation. If you are injured, the Bwaila STI Clinic will give you immediate needed treatment for your injuries. You will not have to pay for this treatment. You will be told where you can get more treatment for your injuries.

There is no program to pay money or give other forms of payment for such injuries either through this clinic or the US NIH. You do not give up any legal rights by signing this consent form.

If you think you have been injured from taking part in this study, call the Principal Investigator at the phone number provided on this consent form. They will let you know what you should do.

**What if you want to stop before your part in the study is complete?**

You can leave this study at any time, without punishment.  The scientists also have the right to stop your participation at any time. This could be because you have had a surprising reaction, or have not followed instructions, or because the whole study has been stopped. If you decide to leave the study, we will ask you to come in for a final visit for collection and storage of blood and urine and STI testing and referral.

**Will you receive anything for being in this study?**

You will receive the money that is the same as about 12 US dollars for your time, effort, and travel to and from the clinic at each study visit.

**Will it cost you anything to be in this study?**

There will be no cost to you for study related visits, physical tests, laboratory tests, or other tasks.

**Who is sponsoring this study?**

This research is funded by Family Health International (FHI 360) through a grant from the US National Institutes of Health (NIH). This means that the research team is being paid by the sponsor for doing the study. The researchers do not, however, have a direct money-related interest with the sponsor or in the final results of the study.

**What if you have questions about this study?**

If you ever have any questions about the study, want to report a social harm, or if you have a research- related injury, you should contact Dr. Mitch Matoga at +265 999 511 726.

If you have questions about your rights as a research participant, you should contact the Head of Secretariat at the National Health Science Research Committee, Dr. Evelyn Chitsa Banda at +265 999 93 69 37 or email at chitsabandaeve@yahoo.com.

By mail:

The Head of Secretariat

Malawi National Health Science Research Committee (NHSRC)

Ministry of Health

Phone: +265 726 422

or by email: [mohdoccentre@gmail.com](mailto:mohdoccentre@gmail.com)

**What if you have questions about your rights as a research participant?**

All research on human volunteers is reviewed by a group of people who work to protect your rights and safety. If you have questions or concerns about your rights as a research subject, or if you would like to get or offer information, you may contact the Institutional Review Board at 919-966-3113 or by email to [IRB_subjects@unc.edu.](mailto:IRB_subjects@unc.edu)

**Consent SIGNATURE PAGE**

If you have read this consent form, or had it read and explained to you, you understand the information, and you voluntarily agree to join the study, please sign your name or make your mark below.

____ I agree to take part in this study.

____ I do not agree to take part in this study.

Hair specimen collection (follow-up visits only):

____ I agree to have hair collected.

____ I do not agree to have hair collected.

If selected for an interview:

____ If selected, I agree to participate in an interview where I will be asked questions about this research, and the interview will be audiotaped.

____ If selected, I do not agree to participate in an interview where I will be asked questions about this research.

**PART A: LITERATE PARTICIPANT**

***Participant is literate***:

________________________________ _____________________________________

Participant Name (print) Participant Signature and Date

________________________________ _____________________________________

Study Staff Conducting Consent Discussion (print) Study Staff Signature and Date

**PART B : ILLITERATE PARTICIPANT**

***Participant is illiterate:***

The study staff must complete this section, ONLY if an impartial witness is available.

The **impartial witness must write participant’s name and date of consent** below**.**

Please thumbrint here if you will allow us to audio record interviews :

Mark or Thumbprint of participant if unable to sign

***Yes : No :***

Mark or Thumbprint of participant if unable to sign

Mark or Thumbprint of participant if unable to sign

Participant Mark or Thumbprint

Participant Name (print) Date

Participant Name and Date Written by………………………………….on…...………

_____________________________ ____________________________ ___________

Study Staff Conducting Consent Study Staff Signature Date

Discussion (print)

_____________________________ _____________________________ ___________

Impartial Witness Name Impartial Witness Signature Date

(print)

**University of North Carolina at Chapel Hill
Parental Permission for a Minor Child (Age 15-17) to Participate in a Research Study**

**_____________________________________________________________________________**

**Consent Form Version Date:** 24 June 2024
**IRB Study # 23-2337**

**NHSRC Protocol #23/10/4208**

**Title of Study:** HPTN 112: Improving HIV prevention among heterosexual cisgender men seeking STI services in Malawi: examining the benefits, acceptability, and associated costs of a systems-navigator- delivered integrated prevention package

**Principal Investigator (US): Sarah Rutstein, MD, PhD**

**Principal Investigator (Malawi): Mitch Matoga, MBBS, MS**
**Principal Investigator Department:** Department of Medicine

**Principal Investigator Phone number (US):** (919) 966-2537
**Principal Investigator Email Addresses:** srutstein@unc.edu; mmatoga@unclilongwe.org

**Funding Source and/or Sponsor:** Division of AIDS (DAIDS), United States (US) National Institute of Allergy and Infectious Diseases (NIAID), US National Institutes of Health (NIH)

**Study Contact Telephone Number**: +265 1 755 056
**Study Contact Email**: mmatoga@unclilongwe.org

______________________________________________________________________________

**CONCISE SUMMARY:** This is a research study. Your child taking part in this research study is voluntary. You and your child do not have to participate, and can leave the study at any time. No matter what you and your child decide, any other care that your child gets at this site will not change.

This study is testing whether adding a systems navigator (similar to a coach or guide) to pre-exposure prophylaxis (PrEP) care, a medicine that can help prevent HIV, improves PrEP use among heterosexual men, compared to the current standard PrEP care.

The study will take about 15-months total. If you and your child choose to enroll in the study, your child will be followed on the study for at least 6 months and up to 12 months. Your child will be asked to give blood and urine specimens for HIV, STI, and other tests. Your child does not need to remain on PrEP to continue participation in this study.

There are very limited risks involved with this study, including (but not limited to) risk of discomfort, dizziness/faintness, and/or bruising, swelling and/or infection from your child’s blood being taken. Your child may also feel feelings of embarrassment or worry when answering questions about their own behaviors and/or receiving HIV counseling or become worried while waiting for their HIV test results. Finally, there is the potential that some of your child’s information may become known to others, even though the study will do everything possible to prevent that.

There may be no direct benefits to your child for being in this study, but others may benefit in the future from information learned from this study. Specifically, information on how to improve HIV prevention services for heterosexual men. In addition, your child will receive HIV and STI counseling and testing as part of the study process and will be referred for treatment as needed.

More information about this study is described in the rest of this form. We will help you understand the information and answer all your questions. You should feel that you understand the study before deciding whether your child will participate. If you agree to your child joining the study, you will be asked to sign your name or make your mark on this form. We will offer you a copy of this form to keep.

**What are some general things you and your child should know about research studies?**

You are being asked to allow your child to take part in a research study. To join the study is voluntary. You may decide to not allow your child to participate, or you may withdraw your permission for your child to be in the study, for any reasons, without penalty. Even if you give your permission, your child can decide not to be in the study or to leave the study early.

Research studies are designed to obtain new knowledge. This new information may help people in the future. Your child may not receive any direct benefit from being in the research study. There also may be risks to being in research studies. Deciding not to be in the study or leaving the study before it is done will not affect your or your child's relationship with the researcher or the health care provider.  If your child is a patient with an illness, your child does not have to be in the research study in order to receive health care.

Details about this study are discussed below. It is important that you and your child understand this information so that you and your child can make an informed choice about being in this research study.

You will be given a copy of this consent form. You and your child should ask the researchers named above, or staff members who may assist them, any questions you have about this study at any time.

**What is the purpose of this study?**

The main purpose of this research study is to understand if it is helpful to use a systems navigator (coach or guide) to support the use of PrEP to prevent HIV. The overall goal of this study is to collect data that will inform future research, helping men from STI clinics reduce risk of getting HIV by taking PrEP.

This study is for adult and adolescent cisgender men who:

- - are age 15 years or older,
  - are seeking services at Bwaila STI Clinic, and
  - have tested HIV negative

**Are there any reasons your child should not be in this study?**

Your child should not be in this study if they are taking part in another study of drugs or medical devices. Your child will be asked to tell the study staff about any other studies they are taking part in or thinking of taking part in. This is very important for their safety.

**How many people will take part in this study?**

About 200 HIV-seronegative heterosexual men will participate in this study from Lilongwe, Malawi.

**How long will your child’s part in this study last?**

Participants who choose to join the study will be in the study for at least 6 months and up to 12 months total.

**What will happen if your child take part in the study?**

During this study, your child will be in one of 2 study groups. Researchers will "randomize" your child into one of the study groups described here. 2 out of every 3 persons enrolled will be assigned to the intervention group and 1 out of every 3 persons enrolled will be assigned to the standard of care group. Random assignment means that your child is put into a study group by chance, like flipping a coin. Neither your child nor the study staff can choose your child’s study group. The table below indicates what services each group will receive.

| Standard of Care Group | Standard PrEP services, including HIV testing and risk reduction counseling |
| --- | --- |
| Intervention Group | Standard PrEP services, including HIV testing and risk reduction counseling. Your child will also be assigned a systems navigator (coach), respond to a brief questionnaire about your sexual activities, and be tested for STIs at any PrEP visit you attend. This coach may also attempt to contact or trace your child for any missed PrEP visit (~7 days late), using information your child provides regarding their preferred tracing time, method (phone or in-person), and location. |

PrEP visits: If your child continues on PrEP, the timing of PrEP visits will be decided by the PrEP nurse at the clinic, according to the Malawi guidelines for PrEP. These visits typically occur every 2-3 months, depending on the kind of PrEP they are taking.

This study is using a Standard of Care Group (also known as a “Control Group”) because we do not yet know whether the intervention being tested in this study improves PrEP use. This kind of intervention has not previously been tested in PrEP care among men in Malawi. Using a Standard of Care Group will allow researchers to assess whether providing the additional intervention resources improves PrEP use. That information can be used to make recommendations to health care providers and leaders about what services should be offered in the future.

All participants will complete the study visit procedures outlined below, regardless of which group they are assigned.

Study visits: The study has at least 3 visits (including the enrollment visit today). Besides this first visit, your child will be asked to come back for a visit ~3 months and ~6 months from now. If your child enrolls early in the study, your child may also be asked to come back for study visits ~9 months and ~12 months from now. At each visit, your child will be asked a series of questions about their behaviors and be tested for HIV and other STIs. Your child may also be asked to participate in a one-on-one interview. Your child should attend these study visits even if they have decided to stop taking PrEP.

***Enrolling in the Study***

If you and your child decide to take part in the study, the Enrollment visit will last about 1-2 hours. During the Enrollment Visit, we will:

- Obtain full written informed consent for the study
- Ask your child where they live and how to contact them
- Ask your child to answer questions in a survey about their sexual activities and overall health
- Ask your child about their previous and current PrEP use
- Provide brief HIV risk reduction counseling
- Conduct a symptom-driven physical exam
- Collect ~10 to 20 mL (about 2 to 4 teaspoons) of blood to make sure your child does have a very early infection with HIV that may not be detected by the standard HIV tests they have received in clinic. If your child has not been tested for syphilis (another STI) at any recent PrEP visits, this blood will also be used to test you for syphilis.
- Collect ~ 10mL of your child’s urine to test for STI testing (Chlamydia and Gonorrhea)
- Store blood (plasma, dried blood spots) and urine samples for study-related testing
- If your child is assigned to the intervention group, study staff will connect your child to their coach to discuss next steps in their PrEP care

***Additional Visits: 2 to 4 additional visits over the next 6 to 12 months.***

If you and you child decide to join the study, after their Enrollment Visit, your child will be asked to come to this location for follow up visits. Participants will be followed for at least 6 months and up to 12 months depending on the time of enrollment.

Each visit will last about 1 hour.

During these follow-up visit(s), we will:

- Ask your child where they live and how to contact them, only if the information your child gave before has changed.
- Ask your child to answer questions in a survey about their sexual activities and overall health
- Ask your child questions about if they are still using PrEP, why, or why not
- Provide HIV risk education counseling
- Conduct a symptom-driven physical exam
- Collect ~10 to 20 mL (about 2 to 4 teaspoons) of blood. Your child’s blood will be used to test them for HIV. If your child has not been tested for syphilis at any recent PrEP visits, this blood will also be used to test your child for syphilis
- Collect ~ 10mL of your child’s urine to test for STI testing (Chlamydia and Gonorrhea) if your child has not been tested for these infections at any recent PrEP visits
- Collect ~ 5 strands of hair, plucking 5 strands of hair from the back of your child’s head with tweezers, so that we can look at medication in the hair root. It will be chosen from a discrete location and be hidden as much as possible. Hair samples will only be collected if your child has sufficient hair (1.0cm) for collection.
- Store blood (plasma, dried blood spots), urine, and hair samples for study-related testing

At your child’s final study visit, we will talk with your child about the end of the study and when the results of the study will be available. Your child may also be asked to participate in a one-on-one interview around the time of their final study visit. Approximately 20 participants will be asked to participate in this type of interview. This would add 30-60 minutes to your child’s visit time, and your child would be reimbursed for this additional time. The interviewer will ask questions about your child’s understanding about HIV prevention, including PrEP, their experience being part of this study, and any challenges they experienced engaging in HIV prevention. If your child is assigned to the intervention group, your child will also be asked about their experience working with the PrEP coach. The interview will be audiotaped so that your child’s responses can be transcribed for analysis. During transcription, any identifying names or places in the interview will be removed from the transcript. After transcription, the recording of the interview will be destroyed. Analysis will be conducted to look for common themes in all interviews conducted. You and your child can decline participation in this interview.

If your child tests positive for HIV at any point in the study, your child will be referred for HIV care and encouraged to start treatment for HIV immediately and your child will have an additional approximately 6-months of study follow-up time from the time your child tests positive. During this time, your child will have blood collected twice – once at 3 months and once at 6 months – to make sure that your child’s body is responding appropriately to HIV treatment. During these visits, we will draw 10-20 mL (about 2-4 teaspoons) of blood to check the amount of HIV in your child’s body. We will ask your child about any medications they are taking, including for HIV treatment. We will also ask them questions about personal perceptions**.** If your child has not started HIV treatment yet, we will encourage them to start. We will also update your child’s contact information. This could happen at a final visit if your child wants to stop the study early.

**If your child stops taking PrEP, we will ask your child to stay in the study*.***

If your child permanently stops taking PrEP during the study for any reason, we will ask your child to continue to come for their regular study visits, but your child will no longer have to undergo certain procedures, like answering questions about taking PrEP, etc. Similarly, if your child miss doses of PrEP or miss PrEP visits, we will ask your child to continue to come for their regular study visits as scheduled.

**If your child gets HIV during the study, we will help your child get care and support.**

We will test your child’s blood for HIV during this study. If your child gets HIV while they are in the study, your child will stop taking PrEP, and we will help your child find the care and support they need.

**Use of stored samples**

In addition to the laboratory testing performed at each study visit, further study-related testing may be performed on blood, urine, and hair samples. This will include testing related to HIV and other infections, including testing for anti-HIV medications and for quality control testing (to confirm results obtained in laboratories). If your child is found to have a very early phase of HIV or gets infected with HIV during the study, some blood may also be used to learn more about HIV viruses, the body’s response to HIV infection, and how HIV is spread in the community. The samples used for this testing will be labeled with your child’s study number and will be tested at special laboratory facilities that may be located in the US and other countries outside of Malawi. The laboratory doing the testing will not know who your child is. Only approved researchers will have access to their samples. Results of this specialized testing will not be returned to the study site or your child. Your child’s samples will not be sold or directly used to produce commercial products or for commercial gain. No host genetic testing will be tested from these samples. Host testing means genetic testing derived from your child.

**What are the possible benefits from being in this study?**

There may be no direct benefits to participants in this study, however, participants and others may benefit in the future from information learned from this study. Specifically, information learned in this study may lead to improved HIV prevention services for heterosexual men in Malawi and the African region. In addition, participants will receive HIV and STI counseling and testing as part of the study process. Participants also will be referred for treatment if needed. Participants who choose to continue PrEP may benefit from the use of PrEP medicines which are known to protect against getting HIV if taken as directed.

**If your child chooses not to be in the study, what other treatment options does your child have?**

Your child’s participation is voluntary. Your child does not have to take part in any of the tests or procedures in the study. Your child should also know that:

- If they decide not to join the study, they will not lose their regular medical care.
- If they join this study and later decide to leave, they will not lose theirregular medical care.
- Your child does not have to join the study to receive HIV prevention medications.
- If you and your child decide not to join the study, your child will still be able to join another study at a later time if there is one available and your child qualifies.

**Can I change my mind about my child participating in this study?**

Yes, you and your child can change your minds at any time. Your child’s participation in this study is completely voluntary. You and your child’s decision to leave the study will not lead to any penalty, or loss of benefits or rights that you and your child would normally have otherwise.

**What are the possible risks or discomforts involved from being in this study?**

It is not expected that this study will expose your child to unreasonable risk. Blood draws may lead to discomfort, feelings of dizziness or faintness, and/or bruising, swelling, and/or infection. There is no foreseeable risk with the donation of hair. Your child may become embarrassed, worried, or anxious when completing their HIV risk assessment and/or receiving HIV counseling. Your child also may become worried or anxious while waiting for their HIV test results. Trained counselors will be available to help your child deal with these feelings. Although the site will make every effort to protect your child’s privacy and confidentiality, it is possible that your child’s involvement in the study could become known to others, and that social harms may result. Social harms could occur if your child is perceived as having HIV or at increased likelihood of getting HIV. Examples of social harms are when your child are treated unfairly or have problems being accepted by their families and/or communities (i.e., because your child could become known as vulnerable to HIV).

**What if we learn about new findings or information during the study?**

You and your child will be told any new information learned during this study that might affect your child’s willingness to stay in the study. For example, if information becomes available that shows that the PrEP coach may have bad effects, you and your child will be told about this. You and your child will also be told when the results of the study may be available, and how to learn about them.

**How will information about your child be protected?**

Every effort will be made to keep your child’s personal information confidential, but we cannot guarantee absolute confidentiality. To keep your child’s information private, their samples will be labeled with a code that can only be traced back to the study clinic. The results of any tests done on these samples will not be included in your child’s health records. Your child’s name, where they live, and other personal information will be protected by the study clinic. Any tracing activities will be done according to you and your child’s stated preferences and will not discuss any information related to the study or study activities. Your child will be identified by a code, and personal information from their records will not be released without their written permission. Any publication of this study will not use your child’s name or identify them personally. Your child’s personal information may be disclosed if required by law.

Clinic staff will have access to your study records. Your child’s records may also be reviewed, under guidelines of the US Federal Privacy Act, by:

The Malawi National Health Science Research Committee (NHSRC)

The University of North Carolina at Chapel Hill (UNC) Institutional Review Board (IRB)

The sponsor of the study (US National Institutes of Health [NIH]) and its contractors.

The US Office for Human Research Protections (OHRP)

Other local, US, or international regulatory authorities/entities

The HPTN (HIV Prevention Trials Network) that is conducting this study

The study staff will also use your child’s personal information, if needed, to verify that your child is not taking part in any other research studies. This includes other studies conducted by Bwaila STI Clinic and studies conducted by other researchers that study staff know about.

Malawi regulations require study staff to report the names of people who get HIV to the local health authority. Outreach workers from the health authority may then contact your child about informing their partners, since they also should be tested. If your child does not want to inform their partners themselves, the outreach workers will offer to contact them, according to the confidentiality guidelines of the Malawi Ministry of Health.

A description of this clinical trial will be available on <http://www.ClinicalTrials.gov>. This Web site will not include information that can identify your child. At most, the Web site will include a summary of the study results. Your child can search this Web site at any time.

**What is a Certificate of Confidentiality?**

Most people outside the research team will not see your name on your research information. This includes people who try to get your information using a court order in the United States. One exception is if you agree that we can give out research information with your name on it or for research projects that have been approved under applicable rules. Other exceptions are for information that is required to be reported under law, such as information about certain harmful diseases that can be spread from one person to another. Personnel of a government agency sponsoring the study may also be provided information about your involvement in the research study.

**What will happen if your child is injured by this research?**

If your child gets sick or injured during the study, contact us immediately.

It is unlikely that your child will be injured as a result of study participation. If your child is injured, the Bwaila STI Clinic will give your child immediate necessary treatment for their injuries. You and your child will not have to pay for this treatment. You and your child will be told where they can get additional treatment for their injuries. There is no program to pay money or give other forms of compensation for such injuries either through this institution or the US NIH. You and your child do not give up any legal rights by signing this consent form.

If your child thinks they have been injured from taking part in this study, call the Principal Investigator at the phone number provided on this consent form. They will let you and your child know what you should do.

**What if you and your child want to stop before your child’s part in the study is complete?**

You can withdraw your child from this study at any time, without penalty. The investigators also have the right to stop your child’s participation at any time. This could be because your child has had an unexpected reaction, or have failed to follow instructions, or because the entire study has been stopped. If you or your child decide to withdraw, we will ask you to come in for a final visit for collection and storage of blood and urine and STI testing and referral.

**Will your child receive anything for being in this study?**

Your child will receive the equivalent of approximately 12USD for their time, effort, and travel to and from the clinic at each scheduled study visit.

**Will it cost you anything for your child to be in this study?**

There will be no cost to you or your child for study related visits, physical examinations, laboratory tests, or other procedures.

**Who is sponsoring this study?**

This research is funded by Family Health International (FHI 360) through a grant from the US National Institutes of Health (NIH). This means that the research team is being paid by the sponsor for doing the study. The researchers do not, however, have a direct financial interest with the sponsor or in the final results of the study.

**What if you or your child have questions about this study?**

If you or your child ever have any questions about the study, want to report a social harm, or if your child has a research- related injury, you or your child should contact Dr. Mitch Matoga at +265 999 511 726.

If you or your child have questions about their rights as a research participant, you should contact the Head of Secretariat at the National Health Science Research Committee, Dr. Evelyn Chitsa Banda at +265 999 93 69 37 or email at chitsabandaeve@yahoo.com.

By mail:

The Head of Secretariat

Malawi National Health Science Research Committee (NHSRC)

Ministry of Health

Phone: +265 726 422

or by email: [mohdoccentre@gmail.com](mailto:mohdoccentre@gmail.com)

**What if you have questions about your child’s rights as a research participant?**

All research on human volunteers is reviewed by a committee that works to protect your child’s rights and welfare. If there are questions or concerns about your child’s rights as a research subject, or if you would like to obtain information or offer input, you may contact the Institutional Review Board at 919-966-3113 or by email to [IRB_subjects@unc.edu.](mailto:IRB_subjects@unc.edu)

**Consent SIGNATURE PAGE**

If you have read this consent form, or had it read and explained to you, you understand the information, and you voluntarily agree to join the study, please sign your name or make your mark below.

____ I agree to have my child take part in this study.

____ I do not agree to have my child take part in this study

Hair specimen collection (follow-up visits only):

____ I agree to have my child’s hair collected.

____ I do not agree to have my child’s hair collected.

If selected for an interview:

____ If selected, I agree to allow my child to participate in an interview where they will be asked questions about this research, and the interview will be recorded.

____ If selected, I do not agree to allow my child to participate in an interview where they will be asked questions about this research.

**PART A: LITERATE PARTICIPANT**

***Participant is literate***:

________________________________ _____________________________________

Participant Name (print) Participant Signature and Date

________________________________ _____________________________________

Study Staff Conducting Consent Discussion (print) Study Staff Signature and Date

**PART B : ILLITERATE PARTICIPANT**

***Participant is illiterate:***

The study staff must complete this section, ONLY if an impartial witness is available.

The **impartial witness must write participant’s name and date of consent** below**.**

Please thumbrint here if you will allow us to audio record interviews :

Mark or Thumbprint of participant if unable to sign

***Yes : No :***

Mark or Thumbprint of participant if unable to sign

Mark or Thumbprint of participant if unable to sign

Participant Mark or Thumbprint

Participant Name (print) Date

Participant Name and Date Written by………………………………….on…...………

_____________________________ ____________________________ ___________

Study Staff Conducting Consent Study Staff Signature Date

Discussion (print)

_____________________________ _____________________________ ___________

Impartial Witness Name Impartial Witness Signature Date

(print)

**University of North Carolina at Chapel Hill**
**Consent to Participate in a Research Study**
**Key Stakeholder Informed Consent for Selected System Navigators and Clinic Staff at Bwaila STI clinic, or Other Stakeholders Participants**
______________________________________________________________________________

**Consent Form Version Date:** June 24 2024
**IRB Study # 23-2337**

**NHSRC Protocol #23/10/4208**

**Title of Study:** HPTN 112: Improving HIV prevention among heterosexual cisgender men seeking STI services in Malawi: examining the benefits, acceptability, and associated costs of a systems-navigator- delivered integrated prevention package

**Principal Investigator (US): Sarah Rutstein, MD, PhD**

**Principal Investigator (Malawi): Mitch Matoga, MBBS, MS**
**Principal Investigator Department:** Department of Medicine

**Principal Investigator Phone number (US):** (919) 966-2537
**Principal Investigator Email Addresses:** srutstein@unc.edu; mmatoga@unclilongwe.org

**Funding Source and/or Sponsor:** Division of AIDS (DAIDS), United States (US) National Institute of Allergy and Infectious Diseases (NIAID), US National Institutes of Health (NIH)

**Study Contact Telephone Number**: +265 1 755 056
**Study Contact Email**: mmatoga@unclilongwe.org

______________________________________________________________________________

**CONCISE SUMMARY**

Things you should know about:

- This interview is part of a larger research study.
- Taking part in this research study is voluntary (your choice). You do not have to participate, and you can leave the study at any time.
- The larger study is testing whether adding a systems navigator (similar to a coach or guide) to pre-exposure prophylaxis (PrEP) care, a medicine that can help prevent HIV, improves PrEP use among heterosexual men, compared to the current standard PrEP care.
- You are being asked to be a part of this interview because we would like to talk with you about your experiences as someone working in areas related to the study, like as a systems navigator, a care provider, a clinic manager, or a policy or program developer.
- There will only be one interview and it will take about an hour.
- There are very limited risks involved with this study, including (but not limited to) risk of discomfort, embarrassment, or worry when answering questions. There is the potential that some of your information may become known to others, even though the study will do everything possible to prevent that.
- There may be no direct benefits to you for being part of this interview, but others may benefit in the future from information learned as part of this interview, specifically, information on how to improve HIV prevention services for heterosexual men.

More information about this interview is described in the rest of this form. We will help you understand the information and answer all your questions. You should feel that you understand the study before deciding whether you will participate. If you agree to join the study, you will be asked to sign your name or make your mark on this form. We will offer you a copy of this form to keep.

**Introduction to Participate**

**What is the key information I should know about this study?**

You have been invited to participate in a research study which includes an interview to discuss your perceptions of and experience with the intervention exploring use of a systems navigator to improve PrEP care for heterosexual men. You are being invited because you are: 1) a provider or supervisor of providers offering STI or HIV care or prevention services; 2) a systems navigator for participants in the study; or 3) involved in the development or implementation of HIV prevention policy or programming at the local or national level in Malawi. This “Key Stakeholder Interview” is an individual discussion about a specific set of topics.

**Why is the study being done?**

We would like to learn about perceived barriers to persistent pre-exposure prophylaxis (PrEP ) use among heterosexual cisgender male STI clinic patients, and potential barriers or facilitators to scaling or sustaining the systems navigation intervention studied during this trial.

**What will happen during the study?**

The interview will be led by an individual that is not directly a member of our research team here at Bwaila clinic but is affiliated with our overall research organization. The individual will not be someone who has a supervisory role for your position.

**How many participants will be in this study?**

Approximately 15 participants will participate in these interviews, conducted approximately 6-12 months after the intervention has first been implemented at the clinic. The information from these interviews will help us better understand how the intervention successfully or unsuccessfully addresses barriers or enhances facilitators of PrEP uptake and persistent PrEP use for men accessing care through the STI clinic. We hope the information we learn will help us promote PrEP use among men from STI clinics, reducing the risk of HIV. The information obtained from these interviews will be combined with the rest of the information that is collected during this research study. Approximately 20 participants from the main study will also be interviewed.

**What other choices do I have?**

Your participation is voluntary. You do not have to take part in this interview. You should also know that:

- If you decide not to join the study, you will not lose your employment or any benefits.
- Your decision to participate or not participate in this study is confidential and we will not inform other people about whether or not you participated.
- If you join this study, you may refuse to answer any of the questions, or stop your participation completely, at any time, for any reason.

**Can I change my mind about participating in this study?**

Yes, you can change your mind at any time. Your participation in this study is completely up to you (voluntary). Your decision to leave the study will not lead to any penalty, or loss of benefits or rights that you would normally have otherwise.

**BEING IN THE STUDY**

**Enrolling in the study.**

If you decide to take part in the study, the interview will last about 1 hour.

During the interview:

- We will obtain full written informed consent for the study.
- The interview will be conducted in a location that assures adequate privacy and confidentiality. The study team will talk with you about this so you know where to go for the interview.
- We will ask you questions that will help us better understand how the intervention successfully or unsuccessfully addresses barriers or enhances facilitators of PrEP uptake and persistent effective use for men accessing care through the STI clinic.

To help assure that we get the best understanding possible from the interview, your answers will be recorded. After the interview is finished, the recording will be typed (called a transcript) and translated by qualified personnel. All identifying information will be removed from the transcript. Your name will not be included on the transcript. The recording will be destroyed after all analysis is completed.

**RISKS OF THE STUDY**

**There may be risks to being in this study.**

As noted, to minimize discomfort and to protect your privacy, the interview will be conducted in a private area that will allow you to speak comfortably without being overheard. The greatest risk may involve your privacy and confidentiality. The steps that the study team has taken to protect your privacy are described below.

**BENEFITS OF THE STUDY**

**There may be no direct benefit to you by participating in the study.**

You may not receive any other direct benefit from participating in this interview; however, the information gathered during this study may help to provide better access to effective PrEP for heterosexual men in Malawi and the African region.

**OTHER INFORMATION ABOUT THE STUDY**

**There is no cost to you to be in this study.**

There will be no cost to you for your participation.

**We will give you about 12USD equivalent for each study visit.**

You will receive the equivalent of approximately 12USD for your time, effort, and any associated travel for this interview.

**We will do our best to protect your private information.**

Every effort will be made to keep your personal information confidential. Your personal information (name, position) will be protected by the study team. Your name, or anything else that might identify you personally, will not be used in any publication of information about this study. Every effort will be made to keep your personal information confidential, but we cannot guarantee absolute confidentiality. Because there are only a few staff at your site working on this study, researchers may know your identity and the information you share.

To keep your information private, your interview will be labeled with a code that can only be traced back to the study clinic. Your name, where you live, and other personal information will be protected by the study clinic. Any publication of this study will not use your name or identify you personally. Your personal information may be disclosed if required by law.

People who may have access to your interview, under guidelines of the US Federal Privacy Act, include:

The Malawi National Health Science Research Committee (NHSRC)

The University of North Carolina at Chapel Hill (UNC) Institutional Review Board (IRB)

The sponsor of the study (US National Institutes of Health [NIH]) and its contractors.

The US Office for Human Research Protections (OHRP)

Other local, US, or international regulatory authorities/entities

The HPTN (HIV Prevention Trials Network) that is conducting this study

Study team members working on this interview portion of the study

A description of the larger clinical trial will be available on <http://www.ClinicalTrials.gov>. This Web site will not include information that can identify you. At most, the Web site will include a summary of the study results. You can search this Web site at any time.

**Contact us at any time if you have questions or problems.**

If you ever have any questions about the study, want to report a social harm, or if you have a research-related injury, you should contact Dr. Mitch Matoga at +265 999 511 726.

If you have questions about your rights as a research participant, you should contact the Head of Secretariat at the National Health Science Research Committee, Dr. Evelyn Chitsa Banda at +265 999 93 69 37 or email at chitsabandaeve@yahoo.com.

By mail (site to complete):

The Head of Secretariat

Malawi National Health Science Research Committee (NHSRC)

Ministry of Health

Phone: +265 726 422

or by email: mohdoccentre@gmail.com

**What if you have questions about your rights as a research participant?**

All research on human volunteers is reviewed by a committee that works to protect your rights and welfare. If you have questions or concerns about your rights as a research subject, or if you would like to obtain information or offer input, you may contact the Institutional Review Board at 919-966-3113 or by email to [IRB_subjects@unc.edu.](mailto:IRB_subjects@unc.edu)

**Consent SIGNATURE PAGE**

If you have read this consent form, or had it read and explained to you, you understand the information, and you voluntarily agree to join the study, please sign your name or make your mark below.

____ I agree to take part in this interview, where I will be asked questions about this research, and the interview will be recorded.

**PART A: LITERATE PARTICIPANT**

***Participant is literate***:

________________________________ _____________________________________

Participant Name (print) Participant Signature and Date

________________________________ _____________________________________

Study Staff Conducting Consent Discussion (print) Study Staff Signature and Date

**PART B : ILLITERATE PARTICIPANT**

***Participant is illiterate:***

The study staff must complete this section, ONLY if an impartial witness is available.

The **impartial witness must write participant’s name and date of consent** below**.**

Please thumbrint here if you will allow us to audio record interviews :

Mark or Thumbprint of participant if unable to sign

***Yes : No :***

Mark or Thumbprint of participant if unable to sign

Mark or Thumbprint of participant if unable to sign

Participant Mark or Thumbprint

Participant Name (print) Date

Participant Name and Date Written by………………………………….on…...………

_____________________________ ____________________________ ___________

Study Staff Conducting Consent Study Staff Signature Date

Discussion (print)

_____________________________ _____________________________ ___________

Impartial Witness Name Impartial Witness Signature Date

(print)
